# Antimicrobial resistance prevalence in bloodstream infection in 29 European countries by age and sex: An observational study

**DOI:** 10.1371/journal.pmed.1004301

**Published:** 2024-03-14

**Authors:** Naomi R. Waterlow, Ben S. Cooper, Julie V. Robotham, Gwenan Mary Knight

**Affiliations:** 1 Centre for Mathematical Modelling of Infectious Diseases, Department of Infectious Disease Epidemiology, EPH, London School of Hygiene and Tropical Medicine, London, United Kingdom; 2 Centre for Tropical Medicine and Global Health, Nuffield Department of Medicine, University of Oxford, Oxford, United Kingdom; 3 NIHR Health Protection Research Unit in Healthcare Associated Infections and Antimicrobial Resistance at University of Oxford in Partnership with the UK Health Security Agency, Oxford, United Kingdom; 4 AMR Centre, London School of Hygiene and Tropical Medicine, London, United Kingdom

## Abstract

**Background:**

Antibiotic usage, contact with high transmission healthcare settings as well as changes in immune system function all vary by a patient’s age and sex. Yet, most analyses of antimicrobial resistance (AMR) ignore demographic indicators and provide only country-level resistance prevalence values. This study aimed to address this knowledge gap by quantifying how resistance prevalence and incidence of bloodstream infection (BSI) varied by age and sex across bacteria and antibiotics in Europe.

**Methods and findings:**

We used patient-level data collected as part of routine surveillance between 2015 and 2019 on BSIs in 29 European countries from the European Antimicrobial Resistance Surveillance Network (EARS-Net). A total of 6,862,577 susceptibility results from isolates with age, sex, and spatial information from 944,520 individuals were used to characterise resistance prevalence patterns for 38 different bacterial species and antibiotic combinations, and 47% of these susceptibility results were from females, with a similar age distribution in both sexes (mean of 66 years old). A total of 349,448 isolates from 2019 with age and sex metadata were used to calculate incidence. We fit Bayesian multilevel regression models by country, laboratory code, sex, age, and year of sample to quantify resistant prevalence and provide estimates of country-, bacteria-, and drug-family effect variation. We explore our results in greater depths for 2 of the most clinically important bacteria–antibiotic combinations (aminopenicillin resistance in *Escherichia coli* and methicillin resistance in *Staphylococcus aureus*) and present a simplifying indicative index of the difference in predicted resistance between old (aged 100) and young (aged 1). At the European level, we find distinct patterns in resistance prevalence by age. Trends often vary more within an antibiotic family, such as fluroquinolones, than within a bacterial species, such as *Pseudomonas aeruginosa*. Clear resistance increases by age for methicillin-resistant *Staphylococcus aureus* (MRSA) contrast with a peak in resistance to several antibiotics at approximately 30 years of age for *P*. *aeruginosa*. For most bacterial species, there was a u-shaped pattern of infection incidence with age, which was higher in males. An important exception was *E*. *coli*, for which there was an elevated incidence in females between the ages of 15 and 40. At the country-level, subnational differences account for a large amount of resistance variation (approximately 38%), and there are a range of functional forms for the associations between age and resistance prevalence. For MRSA, age trends were mostly positive, with 72% (*n* = 21) of countries seeing an increased resistance between males aged 1 and 100 years and a greater change in resistance in males. This compares to age trends for aminopenicillin resistance in *E*. *coli* which were mostly negative (males: 93% (*n* = 27) of countries see decreased resistance between those aged 1 and 100 years) with a smaller change in resistance in females. A change in resistance prevalence between those aged 1 and 100 years ranged up to 0.51 (median, 95% quantile of model simulated prevalence using posterior parameter ranges 0.48, 0.55 in males) for MRSA in one country but varied between 0.16 (95% quantile 0.12, 0.21 in females) to −0.27 (95% quantile −0.4, −0.15 in males) across individual countries for aminopenicillin resistance in *E*. *coli*. Limitations include potential bias due to the nature of routine surveillance and dependency of results on model structure.

**Conclusions:**

In this study, we found that the prevalence of resistance in BSIs in Europe varies substantially by bacteria and antibiotic over the age and sex of the patient shedding new light on gaps in our understanding of AMR epidemiology. Future work is needed to determine the drivers of these associations in order to more effectively target transmission and antibiotic stewardship interventions.

## Abstract 2

Naomi R Waterlow and colleagues leverage data from 29 countries across Europe to determine how antimicrobial resistance varies by age and sex.

## Author summary

**Why was this study done?:**

- Antimicrobial resistance (AMR) is a major global public health threat, but little is known about how the prevalence of resistance varies with age and sex.

**What did the researchers do and find?:**

- We explored patterns of resistance prevalence and incidence by age and sex in routinely collected data from bloodstream infections (BSIs) across Europe for 8 bacterial species.
- We fitted a Bayesian multilevel regression model to quantify the variation nationally and subnationally.
- Distinct patterns in resistance prevalence by age were observed across Europe for different bacteria.
- Sex was often only weakly associated with resistance, except across ages in *Escherichia coli* and *Klebsiella pneumoniae*, and at younger ages for *Acinetobacter* species, where it was higher in males.

**What do these findings mean?:**

- These findings highlight important gaps in our knowledge of the epidemiology of AMR that are difficult to explain through known patterns of antibiotic exposure and healthcare contact.
- Differences in AMR burden by age and sex may be explained by cultural differences between countries as well as variation in pathways to infection between bacteria.
- Our findings suggest that there may be value in considering interventions to reduce AMR burden that take into account important variations in AMR prevalence with age and sex.
- This study is limited by the nature of routine surveillance, the lack of open availability of disaggregated data, and the model structures explored.

## Introduction

Antimicrobial resistance (AMR) is a global public health priority [1]. Understanding how it will be affected by the dramatic demographic shifts that are underway worldwide is a key knowledge gap. The World Health Organisation (WHO) has estimated that 1 in 5 people in the world will be aged 60 years or older by 2050 [2]. Incidence of bacterial infections is known to increase by age [3] and vary by sex [4]. The higher burden of infection in older age groups [5,6], results in higher antibiotic exposure and higher contact with healthcare settings which are known hotspots of resistant bacteria transmission. However, there is not a simplistic increase in resistance in all pathogens by age. Determining how the above interact to drive the dynamics of drug-resistant infections (DRIs) is a vital part of understanding how best to tackle AMR.

Age- and sex-disaggregated data are collected by most routine AMR surveillance schemes. The WHO Global Antimicrobial Resistance and Use Surveillance System (GLASS) requests age- and sex-stratifications from reporting countries [7]. However, this data are not currently openly available at low age-band segregation (i.e., more than 4 broad categories) with sex—for example, not from the WHO GLASS dashboard [8] nor the European Centre for Disease Prevention and Control (ECDC) ATLAS dashboard [9] nor the US Centres for Disease Control and Prevention [10]. The recent WHO reports also have not presented analysis of how resistance prevalence varies by these demographic factors [11]. The dramatic, often exponential, increase in infection incidence with older age has been reported in several places [12–15] as well as the differences by sex [16]. However, how this burden is split into resistant or susceptible infection by patient age and sex is relatively rarely reported. Multiple attempts to predict AMR burden are hampered by a basic lack of surveillance data and yet factors such as sex and age are variables that are nearly always available for analysis.

The importance of age being linked to variation in AMR has been graphically explored before for Europe [17] and more comprehensively in single setting studies (e.g., [18–20]). Complex statistical analysis based on the Global Burden of Disease methods have produced age- and sex-specific estimates of mortality rates by European country attributable to all AMR [21]. However, to our knowledge there has been no comprehensive analysis of the relationship between age and AMR in infection between bacteria across multiple countries. This is despite the wide awareness of age-specific effects for infection that have only been emphasised by the Coronavirus Disease 2019 (COVID-19) pandemic [22].

Despite sex being a well-established risk factor for specific bacterial infections such as urinary tract infections, how prevalence of DRI varies between the sexes (and genders) is vastly underexplored in the literature [23]. This is despite many studies of infections caused by specific bacterial pathogens or syndromes finding a difference in resistance prevalence in infection between the sexes [24–29]. In 2018, the WHO called for countries to take the first step to better considering “gender and equity” in National Action Plans for AMR [30], which have historically lacked such considerations (e.g., in Southeast Asia [31]).

Prevalence of resistance in infection is known to vary between countries [8,11] and subnationally, by factors such as deprivation level [32–34]. This may be linked in part to differences in healthcare structures and antibiotic usage [35,36]. Other national level healthcare structures and cultural differences are likely to have wider impacts on AMR patterns by age and sex. For example, variation in birth rates by age between countries [37], as well as type of birth (vaginal versus cesarean) [38] will impact the type of antibiotic as well as healthcare exposures in women. Determining how these cultural factors interplay with biological factors as we age and across sexes is key to understanding the nuanced interventions required to tackle AMR.

Here, we use a large dataset of routinely collected information on bloodstream infections (BSIs) to explore trends in prevalence of antibiotic resistance and infection by age and sex across Europe.

## Methods

### Ethics statement

This work to analyse routinely collected data was approved by the London School of Hygiene and Tropical Medicine ethics board (ref 28157).

### Data

We analysed the European Antimicrobial Resistance Surveillance Network (EARS-Net) patient level data for 2015 to 2019 reported to European Centre for Disease Prevention and Control (ECDC) by Austria, Belgium, Bulgaria, Cyprus, Czechia, Germany, Denmark, Estonia, Greece, Spain, Finland, France, Croatia, Hungary, Ireland, Iceland, Italy, Luxembourg, Latvia, Malta, the Netherlands, Norway, Poland, Portugal, Romania, Sweden, Slovenia, Slovakia, and the United Kingdom [39, 40]. Countries were anonymised using a random 3-letter code, which is used throughout the paper as data at this level of detail by age and sex is not publicly available, because the required national level permissions are not in place [39]. EARS-Net collects routine clinical antimicrobial susceptibility testing (AST) results, alongside some patient data, including sex and age, from EU/EEA countries (we use the term European throughout). The general quality and comparability of the data is evaluated through a standard annual external exercise [41] with the AST results taken from shared protocols [40,42]. The data consists of AST for the first blood and/or cerebrospinal fluid isolate (<0.7% of this dataset) of every patient with an invasive infection associated with one of the pathogens under surveillance (Section 2 in S1 Appendix). Levels of coverage are discussed and explored in the calculation of incidence (see below and Section 3 in S1 Appendix). In our main analysis, we exclude individuals aged 0, due to their stark difference in immune dynamics, linked in part to waning maternal antibodies [43,44], and healthcare contact patterns of this subset of critically ill children, but run a sensitivity analysis including them.

Individual patient data from EARS-Net was extracted with information on the age and sex of the patient, resistance presence, laboratory code, year of sample, and reporting country. For resistance prevalence calculations, we used the susceptibility test result data for 2015 to 2019 in those aged one or older, with data on age and sex. We analysed missing data both in terms of (a) distribution of age and sex within those not tested for resistance; and (b) resistance prevalence in those without age and sex information. For incidence calculations, we included all isolates with recorded age and sex values, for those aged one or older.

We used the United Nations subregion definitions, except for Cyprus, which was grouped with Southern Europe (instead of being the only Western Asia country). Some susceptibility data grouped results for multiple antibiotics together: “aminopenicillins” are ampicillin or amoxicillin, “3G cephalosporins” are cefotaxime, ceftriaxone or ceftazidime, “fluoroquinolones” are ciprofloxacin, levofloxacin or ofloxacin, “aminoglycosides” are gentamicin or tobramycin, “macrolides” are azithromycin, clarithromycin or erythromycin, “penicillins” are penicillin or oxacillin, “carbapenems” are imipenem/meropenem (Table 1 in ECDC reports [45]). Where we had multiple susceptibility results for individual antibiotic within a drug family (beta-lactam), we grouped antibiotics by AWaRe classifications [46]. We follow the ECDC analysis and assume “sex” rather than “gender” was recorded in the data.

1. *Prevalence of resistance in infection by age and sex*
  Using the cleaned data, we explored variation in patterns in aggregated sex- and age-based resistance prevalence in infection at the European and subregional levels.
2. *Incidence of infection by age*
  Following the methods of Cassini and colleagues [47] (Sections 3 and 4 in S1 Appendix), it was assumed that all eligible invasive isolates are reported by the participating laboratories. The estimated coverage of these laboratories was then used as an inflation factor to calculate the number of BSIs. Data for country coverage was taken from previous EARS-Net reports and the Cassini and colleagues estimates for 2015, 2018, 2019, and 2020. The incidence of infection in each of these years was calculated by dividing the number of isolates from patients in each 5-year age and sex band by the corresponding population sizes from the World Bank DataBank [48], up to a pooling of all those aged 80 or older. We report an estimated incidence for 2019.
3. *Trend analysis for resistance proportion by age*
  Multilevel regression models were fitted to the ECDC data to understand the impact of including age and sex in models of resistance prevalence. We used a Bayesian framework using the *R* package *brms* [49] and ran models using the No U-turn Sampling separately for each bacteria-antibiotic combination, using data from 2015 to 2019. Individual-level data was aggregated to group level by country, laboratory code, sex, age, and year of sample and standardised as appropriate (Section 5 in S1 Appendix). Models were considered converged if the Rhat was <1.1, a sufficient Effective Sample Size for each parameter was reached and we checked for divergent transitions (Section 5 in S1 Appendix). We initially ran 3,000 iterations and extended this to 5,000 for those models that had not reached convergence at this point. Country and laboratory code were included as substantial variation was observed between them under a variance-components model (Section 5 in S1 Appendix). Only the sexes “male” and “female” were included in the analysis and records missing age or sex were dropped.

**Table 1:** Baseline characteristics of data included for exploration of resistance prevalence in BSIs across Europe for 15 antibiotics across 8 bacteria in 29 countries for 2015–2019. The split of the susceptibility results by country is given in Table A2 in S1 Appendix. The definition of an “antibiotic” was linked to the data given so is at different levels (e.g., separate aminoglycosides were included as well as an antibiotic category of “aminoglycoside”). Fluroquinolones resistance was labelled the same across species though there were species-specific definitions. Age given in years. MRSA covers oxacillin and cefotoxin. 3G = third-generation. Pip-taz = piperacillin-tazobactam. SD = standard deviation.

Characteristics		All		Female	Male
**Susceptibility results (*n*(%))**		6,862,577		3,211,521 (47%)	3,651,056 (53%)
**Number of patients**		944,520		444,778 (47%)	538,723 (53%)
**Age (mean, SD)**		66 (19.6)		66 (20.6)	66 (18.7)
**Number of susceptibility results** **Range** **(mean; SD)**	**Country** **(*n* = 29)**	8,391–1,137,670 (236,641; 272,901)		4,268–547,018 (110,742; 128,611)	3,989–590,652 (125,898; 144,594)
	**Bacteria** **(*n* = 8)**	82,085–4,032,238 (857,822; 1,313,543)		35,084–2,122,471 (401,440; 703,758)	47,001–1,909,767 (456,382; 613,016)
	**Antibiotic** **(*n* = 15)**	74,342–1,184,265 (457,505; 359,541)		26,635–545,803 (214,101; 176,634)	41,597–638,462 (243,404; 183,812)
			**Total number of susceptibility results**		
**Bacteria**	**Antibiotic**	**Age (mean)**	**All**	**Female**	**Male**
*Acinetobacter* species	Amikacin	61	15,298	6,491	8,807
	Aminoglycosides	61	22,174	9,506	12,668
	Carbapenems	61	22,329	9,543	12,786
	Fluroquinolones	61	22,284	9,544	12,740
*Enterococcus faecalis*	Aminopenicillins	69	89,517	29,780	59,737
	High-level aminoglycoside	68	57,831	19,731	38,100
	Vancomycin	69	91,995	30,585	61,410
*Enterococcus faecium*	Aminopenicillins	67	59,674	23,444	36,230
	High-level aminoglycoside	67	36,471	14,314	22,157
	Vancomycin	67	61,855	24,252	37,603
*Escherichia coli*	Amikacin	67	349,169	182,658	166,511
	Aminoglycosides	67	618,839	326,059	292,780
	Aminopenicillins	67	532,227	280,669	251,558
	Carbapenems	67	604,618	318,300	286,318
	3G cephalosporins	67	612,331	322,977	289,354
	Ertapenem	67	264,862	140,223	124,639
	Fluoroquinolones	67	619,648	326,594	293,054
	Pip-taz.	67	430,544	224,991	205,553
*Klebsiella pneumoniae*	Amikacin	66	96,924	37,872	59,052
	Aminoglycosides	67	148,410	58,048	90,362
	Carbapenems	67	146,551	57,284	89,267
	3G cephalosporins	67	148,192	57,977	90,215
	Ertapenem	67	67,062	26,248	40,814
	Fluoroquinolone	67	149,122	58,325	90,797
	Pip-taz.	68	108,092	41,909	66,183
*Pseudomonas aeruginosa*	Amikacin	66	59,968	21,797	38,171
	Aminoglycoside	67	76,015	27,245	48,770
	Carbapenem	67	76,055	27,251	48,804
	Ceftazidime	67	74,342	26,635	47,707
	Fluoroquinolone	67	75,944	27,236	48,708
	Pip-taz.	67	73,729	26,430	47,299
*Staphylococcus aureus*	Fluoroquinolone	64	258,605	97,323	161,282
	MRSA	64	286,731	107,916	178,815
	Rifampicin	64	232,585	87,379	145,206
*Streptococcus pneumoniae*	3G cephalosporins	63	56,908	25,704	31,204
	Fluoroquinolone	63	58,662	26,781	31,881
	Macrolide	63	79,731	36,814	42,917
	Penicillins	63	77,283	35,686	41,597

BSI, bloodstream infection; MRSA, methicillin-resistant *Staphylococcus aureus*.

Thus, for each bacteria-antibiotic combination, our data consisted of multiple groupings of individual samples of a bacterium tested for resistance to that antibiotic. Each grouping *i* had a unique combination of country (c), laboratory code (l), sex, age, and year of sample and hence a linked number of samples (*n*) and proportion resistant (*p*).

For each bacteria-antibiotic combination, we ran a multilevel logistic regression model to predict the probability of an isolate being resistant to the antibiotic, assuming a binomial distribution over the number of samples in each grouping. Our model included both age and sex terms (Eqs 1 and 2).


$$y_{i}∼Binomial(n_{i},p_{i})$$ (1)

$$p_{i}=\beta_{0}+\beta_{t}*t_{i}+\beta_{a}*{age}_{i}+\beta_{a^{2}}*{age^{2}}_{i}+\beta_{g}*{sex}_{i}+\beta_{ag}*{age}_{i}*{sex}_{i}+v_{c(i)}+v_{c(i)a(i)}*{age}_{i}+u_{c(i),l(i)}+\epsilon_{i}$$ (2)

Where *y* is the resistance variable, taking a value of 0 or 1, *n* is the number of samples, and *p* the probability of the sample being found to be resistant (NAs were excluded, Section 5 in S1 Appendix). The subscripts *c*, *l*, and *i* denote country, laboratory code, and grouping level. *β*_0_ is the overall intercept, *β*_*t*_ is slope coefficient for time, and *t*_*i*_ is year. *ϵ*_*i*_ is the residual error, *u*_*c*(*i*),*l*(*i*)_ is the level-2 random error on laboratory code, and *v*_*c*(*i*)_ is the level-3 random error on country. *β*_*a*_ is the age effect coefficient, $\beta_{a^{2}}$ is the age squared effect coefficient, and *v*_*c*(*i*)*a*(*i*)_ is the country-level age effect coefficient. *β*_*g*_ is the sex effect coefficient and *β*_*ag*_ is the sex and age interaction coefficient. The sex variable takes a value of 0 or 1, being 1 for males. We chose to include an age^2^ explanatory variable, as previous analysis had identified nonlinear trends with age and antibiotic use (a key driver of resistance) is known to have nonlinear, often quadratic relationship with age [50].

All random errors are assumed to be normally distributed, and we assume the default priors on all covariates in the main analysis from the *brms* package [49], but run a sensitivity analysis with weakly informative regularising priors.

To determine an overall impact of age for each bacteria-antibiotic combination and country, we calculated the difference in the model-predicted proportion resistant between young individuals (aged 1) and older individuals (those aged 100), using the posterior predictions from the model fit. We did this across all posterior samples, from which we calculated the median and 95% quantiles. This simplifying index was chosen to capture and illustrate one aspect of the differences seen (the change between young and old). We explore the robustness of this index to different definitions of young (age 1 to 20) and older (age 50 to 100).

### Sensitivity analysis

We explored further data disaggregation of incidence by patient location when the sample was taken (inpatient versus outpatient and the hospital unit or ward type, e.g., haematology or emergency department). For incidence analysis, we explored varying the inflation factor for the incidence of infection to check robustness of age and sex patterns.

For the modelling analysis, we explored including samples from individuals aged 0, including regularising priors and using a model selection-based approach. These sensitivity analyses were run for MRSA.

## Results

Our analysis was in 3 stages. Firstly, we explored the trends in resistance prevalence by age and sex across Europe. Secondly, we estimated and quantified the incidence of infection for each of the bacterial species by age and sex. Thirdly, we quantified the proportion of those infections that were due to resistant bacteria for different bacteria-antibiotic combinations by age and sex, country and subnational indicator (laboratory) by fitting multilevel models. We exemplify the outputs of the multilevel modelling by exploring results for 2 examples: aminopenicillin resistance in *E*. *coli* and methicillin resistance in *S*. *aureus* chosen for their large contributions (>40%, [51]) to the aetiology of BSIs, high number of samples (Table 1) and associated important resistance in Europe and globally [45,52].

### Data

For the resistance prevalence calculations, we used a total of 6,862,577 susceptibility results (74% of the original available) across 29 European countries for 15 antibiotic groupings in 8 bacteria for 2015 to 2019 (Table 1, Section 2 in S1 Appendix). The average age of the patients with BSIs from whom the samples came was 66 (standard deviation: 19.6), and the majority were male (53%). The age- and sex-distribution was similar across all bacteria and antibiotic groupings. All countries and bacteria were included the analysis despite large variations in the number of susceptibility results reflecting the aetiology of BSIs and population sizes (Table 1, Section 2 in S1 Appendix).

For incidence calculations, we used all isolates with age and sex information taken from patients aged 1 or older (a total of 349,448 isolates in 2019) (Section 3 in S1 Appendix). This was 91% of all isolates, with a range between 5,637 and 154,071 isolates used across Europe in 2019.

### Resistance prevalence: European level

**Fig 1:**
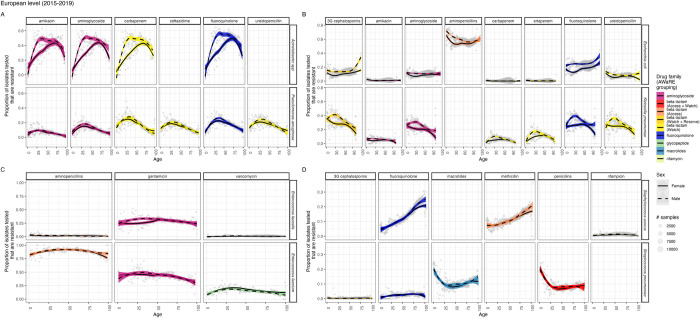
Trends in resistance prevalence in BSIs vary by antibiotic, bacteria, and demographic factors across Europe. The proportion of isolates from BSIs tested (y axis) that are resistant to each antibiotic (panel) within drug families and AWaRe groupings (for beta-lactams) (colour) for each bacteria (row) is shown for all European data over 2015–2019 by age (x axis) for the 4 gram-negative (A+B) and 4 gram-positive bacteria (C+D). Data is shown as points with number of samples indicated by size of point. Shaded areas are 95% confidence intervals around the LOESS fit line by sex (linetype). AWaRe groupings were used here to better distinguish clinically important subsets within the beta-lactam family. Blank panels indicate where no data was available. BSI, bloodstream infection; LOESS, locally estimated scatterplot smoothing.

At the European level, there were clear nonlinear differences in the prevalence of resistance in infection by age and sex for different bacteria-antibiotic combinations (Fig 1). These patterns were robust across subregions of Europe (Section 1 in S2 Appendix). However, prevalence of resistance was generally higher in Southern and Eastern Europe, with stronger age-related trends (e.g., for methicillin resistance in *S*. *aureus* and across *Acinetobacter* species). The age-associated patterns varied more within drug-families than within certain bacteria (patterns within each colour are more different than within each row of Fig 1). For example, patterns of resistance across drug families were highly similar across all antibiotics included for some bacteria such as *Acinetobacter* species (Fig 1A) while there was substantial variation within resistance proportions by age for fluroquinolones (blue data, Fig 1). For some bacterial species, such as *S*. *pneumoniae* (Fig 1D), resistance trends were similar for subsets of antibiotics. Sex has little impact on many of the age-related trends except for *E*. *coli* and *K*. *pneumoniae*, and at younger ages for *Acinetobacter* species (Fig 1B).

### Incidence

**Fig 2:**
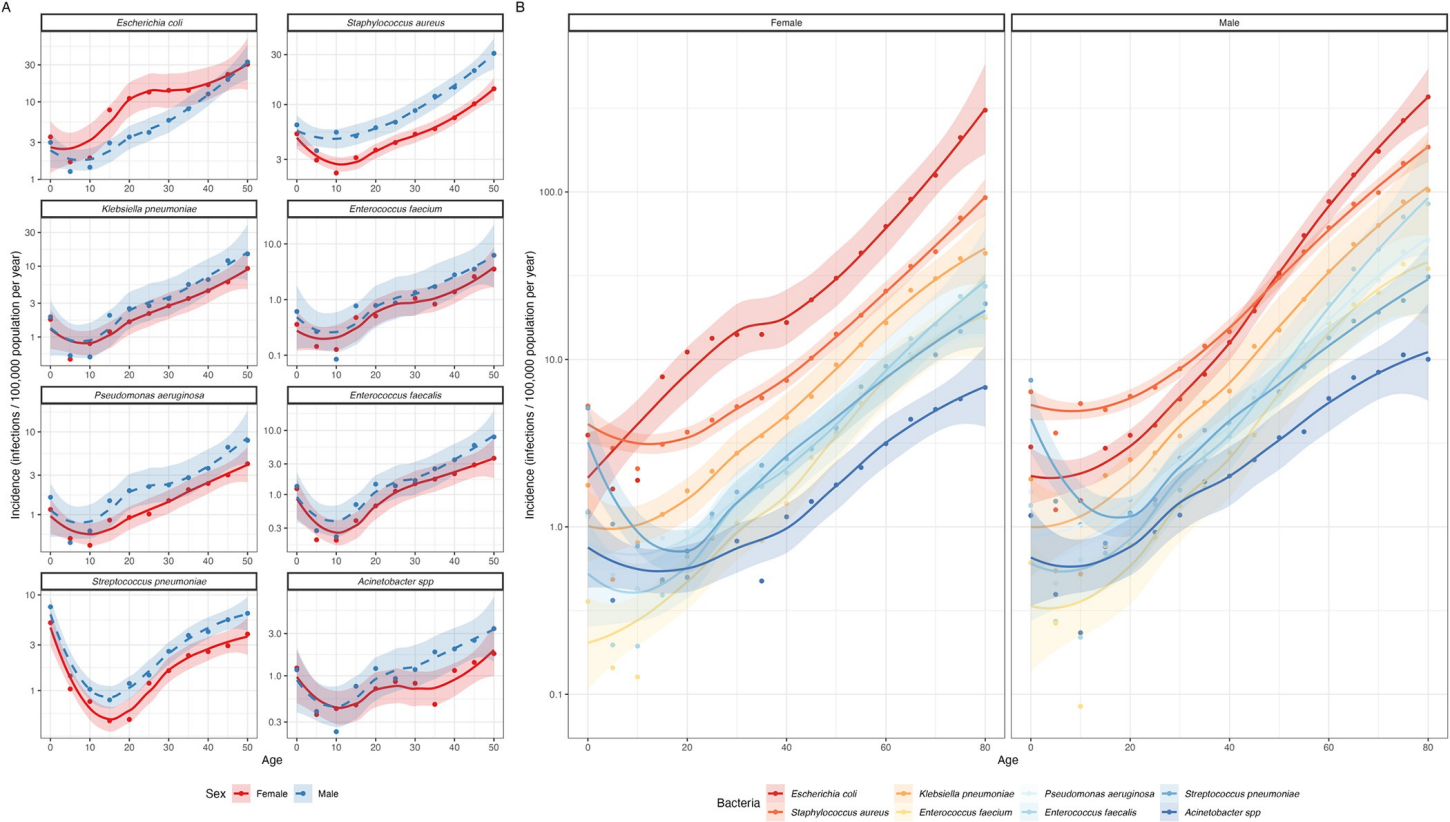
Incidence of BSIs per 100,000 population in 2019 across European countries for 8 bacterial pathogens. To demonstrate trends clearly, the incidence is split to show (A) patterns in the first 50 years of life by sex and bacteria and then (B) shows the lifelong trends split by sex (panel) and bacteria (colour). Shaded areas are 95% confidence intervals using an LOESS fit. Infections in individuals younger than 0 are excluded, and those aged 80 and older are pooled into the 80-yr data point. The y axis is on a log scale (base 10). BSI, bloodstream infection; LOESS, locally estimated scatterplot smoothing.

As expected, across Europe, BSI incidence was u-shaped for most bacteria, substantially increasing with older ages with clear differences between the sexes (Fig 2). Men had a higher incidence of infections from approximately age 35 onwards, except for *E*. *coli* between ages 15 and 40 where women had a higher incidence (Fig 2A) and *S*. *pneumoniae*. These patterns were robust at the country level and over time (Section 2 in S2 Appendix). Differences in infection incidence between the pathogens reflect the overall burden in infection, with ranking incidence rates being (from highest): *E*. *coli*, *S*. *aureus*, *K*. *pneumoniae*, *E*. *faecium*, *P*. *aeruginosa*, *E*. *faecalis*, *S*. *pneumoniae*, *Acinetobacter* species.

The combination of these age-related trends in number of infections (Fig 2) with those in proportion resistant (Fig 1) lead to exponential increases in the number of resistant infections with age among adults (Section 3 in S2 Appendix) for all bacteria-antibiotic combinations.

### Resistance prevalence: Model results

Our logistic model converged for 34 bacteria-antibiotic combinations (89%) (Section 4 in S1 Appendix) and had substantial effects for at least one of age, age^2^, or the interaction between age and sex (Table 2). Sex had less of a clear importance for many bacteria-antibiotic combinations, with at least one of the sex intercept or interaction terms being substantial for 19 of the 34 bacteria-antibiotic combinations. Full results for all bacteria-antibiotic combinations can be found in S3 Appendix.

**Table 2:** Heatmap of the values of the fixed effect parameters for each bacteria-antibiotic model. Orange indicates a positive coefficient and blue indicates a negative coefficient (in both cases, where the 95% credible intervals of the posterior parameter estimate do not cross 0). White indicates the coefficient was neither positive nor negative (i.e., posterior credible intervals cross 0). An equivalent table with the parameter values can be found in the supplement (Section 4 in S2 Appendix); (m) indicates that the parameter is the coefficient for males. Fluoroquinolone resistance definitions varied between species (S1 Appendix). MRSA primarily indicates oxacillin or cefoxitin resistance, but other markers are accepted for oxacillin, if oxacillin was not reported. See protocol for details [40].

Bacteria	Antibiotic	Year	Age	Age^2^	Sex(m)	Age:sex(m)
*Acinetobacter* species	Amikacin					
	Aminoglycosides					
	Carbapenems					
	Fluroquinolones					
*Enterococcus faecalis*	High-level aminoglycoside					
	Vancomycin					
*Enterococcus faecium*	Aminopenicillins					
	High-level aminoglycoside					
*Escherichia coli*	Amikacin					
	Aminoglycosides					
	Aminopenicillins					
	Carbapenems					
	Fluoroquinolones					
	Third-generation cephalosporins					
	piperacillin-tazobactam					
*Klebsiella pneumoniae*	Amikacin					
	Aminoglycosides					
	Carbapenems					
	Ertapenem					
	Fluoroquinolones					
	Third-generation cephalosporins					
	Piperacillin-tazobactam					
*Pseudomonas aeruginosa*	Amikacin					
	Aminoglycosides					
	Carbapenems					
	Ceftazidime					
	Fluoroquinolone					
	Piperacillin-tazobactam					
*Staphylococcus aureus*	Fluoroquinolone					
	MRSA					
	Rifampicin					
*Streptococcus pneumoniae*	Macrolide					
	Penicillins					
	Fluoroquinolone					

MRSA, methicillin-resistant *Staphylococcus aureus*.

The nonlinear trends in resistance prevalence by age were approximated in the model by a combination of linear and quadratic age effects, with variable combinations of associations leading to varying negative and positive coefficients (Table 2). The decomposition of these effects can be seen in Section 4 in S2 Appendix.

### National and subnational variation in resistance prevalence

We found that resistance prevalence varied substantially by country (as expected) but also within country, with subnational differences accounting for a large amount of resistance variation (approximately 38%, Section 5 in S2 Appendix). There were a range of shapes (both convex and concave) for the associations by sex between age and resistance prevalence (S3 Appendix).

**Fig 3:**
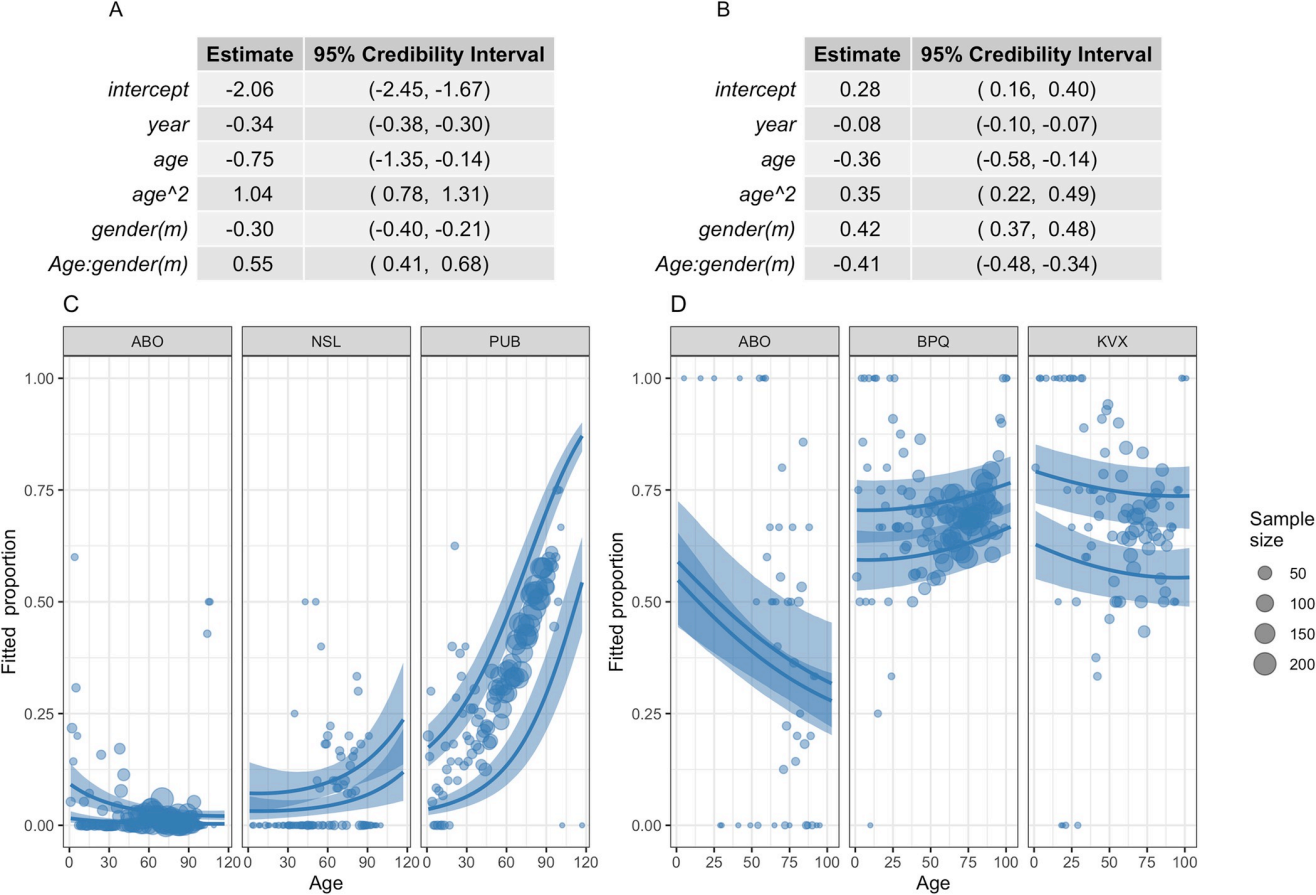
**Model parameters and example model predictions for MRSA (left) and aminopenicillin resistance in *E*. *coli* (right).** (A, B) Model parameters. (m) indicates that the parameter is the coefficient for males. (C, D) Data (points) and model predictions (lines) with 95% credible intervals (ribbons) for males for the most extreme (left and right panels) and the middle country (middle panel) estimated age slope. Each country has 2 lines, depicting the predictions for the most extreme laboratories in the country. Data sample size (shown by dot size) is grouped across years and laboratories. MRSA primarily indicates oxacillin or cefoxitin resistance, but other markers are accepted for oxacillin, if oxacillin was not reported. See protocol for details [41]. Country labels are a random anonymised 3-letter code used for this study only but consistent across all analyses. MRSA, methicillin-resistant *Staphylococcus aureus*.

**Fig 4:**
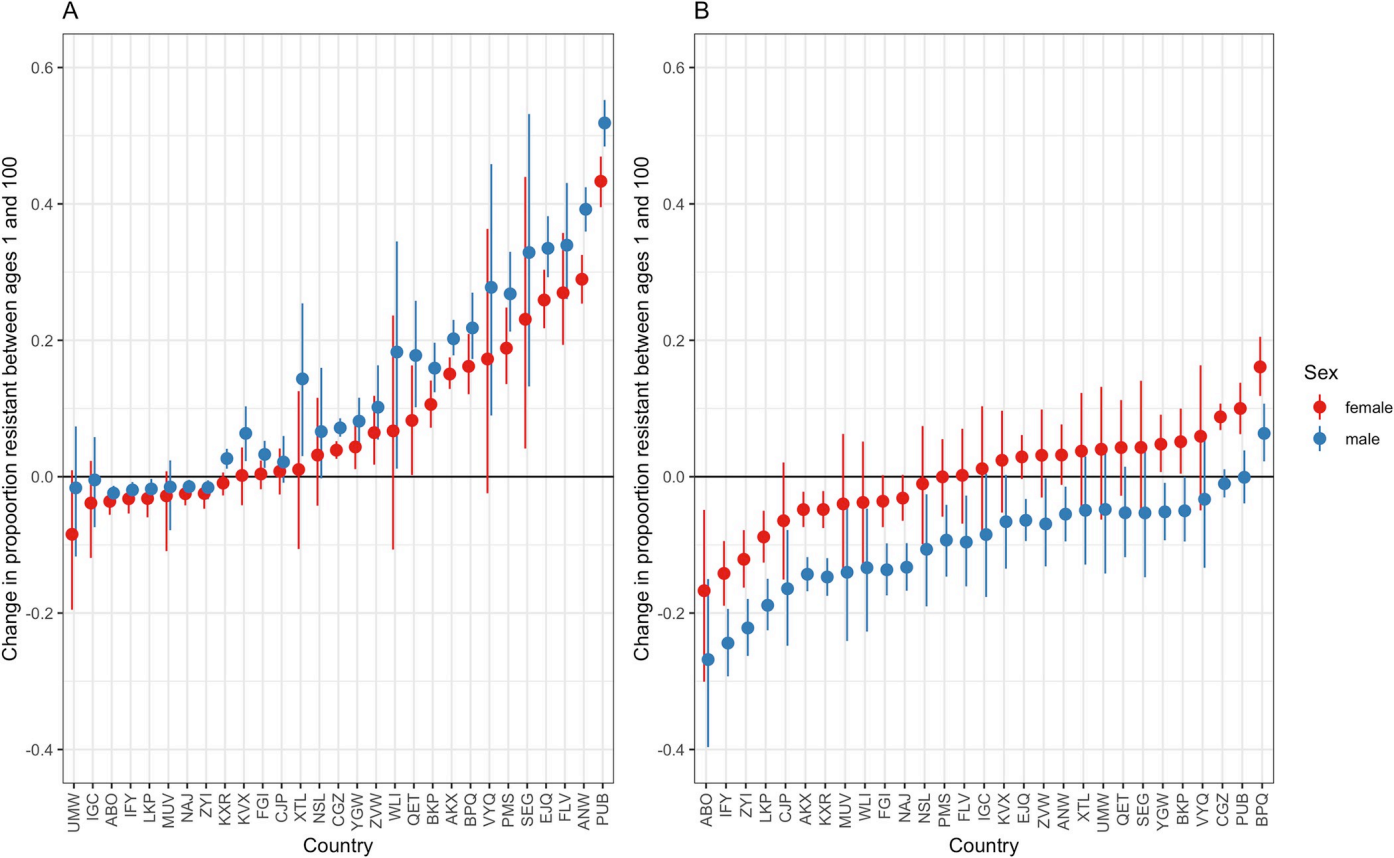
Change in model-predicted proportion resistant between older (age 100) and younger (age 1) patients. This index is shown for each country and sex for MRSA (A) and aminopenicillin resistance in *E*. *coli* (B), with the point indicating the median and the error bars the 95% quantiles across model predictions. Country labels are random anonymised 3-letter code used for this study only but consistent across all analyses. MRSA, methicillin-resistant *Staphylococcus aureus*.

To demonstrate this substantial national and subnational variation, we focus on 2 example bacteria-antibiotic combinations, demonstrating model predictions of subnational (laboratory level) variation for countries across the range of coefficients for age effect (Fig 3) and the country-level differences in resistance between the young and old (Fig 4).

For MRSA, most countries (e.g., for males, 72%, 21/29 countries) have a positive trend with age (driven in part by a high age^2^ coefficient, Fig 3A), while for aminopenicillin resistance in *E*. *coli* the age trend is mostly negative (males, 93%, 27/29 countries), with a lower proportion resistant with age (Figs 3B and 4). Country-level effects (panels, Fig 3C and 3D) as well as laboratory (subnational) effects (lines, Fig 3C and 3D) were highly important in capturing proportion resistant by age.

There were important sex effects in both the intercept and age-slope terms for both MRSA and aminopenicillin-resistance in *E*. *coli* (Fig 3A and 3B), which resulted in clear differences in age-association by sex in these examples (Fig 4).

For MRSA, a higher proportion of samples were predicted to be resistant at older (age 100) than younger (age 1) ages (Fig 4A). The magnitude of this difference varied but reached a maximum difference in proportion of 0.51 (median, 95% quantile 0.48, 0.55, for country PUB) between males aged 1 and 100. For aminopenicillin resistance in *E*. *coli* for many countries, a lower proportion of samples were predicted to be resistant at age 100 than age 1 (Fig 4B), with the magnitude of the age effect varying from 0.16 (median, 95% quantiles 0.12, 0.21, country BPQ female) to −0.27 (95% quantiles −0.4, −0.15, country ABO, male). The trends in this simplifying index by country were robust to comparing resistance prevalence at ages 50 to 100 to age 1, and at ages 1 to 20 to that at age 100 (Section 6 in S2 Appendix).

### Variations in resistance prevalence with age by country, antibiotic, and bacteria

While the 2 bacteria-antibiotic samples chosen above show substantial trends, there are many bacteria-antibiotic combinations where no age or sex trend is seen, or where there is little similarity within a bacteria-antibiotic combination across countries (completely overlapping confidence intervals when looking at the difference between resistance prevalence at ages 1 to 100) (Section 5 in S2 Appendix). Additional bacteria-antibiotic combinations where a >5% change in resistance proportion between old and young (ages 100 versus 1) for multiple countries was seen include third-generation cephalosporin resistance in *E*. *coli* and *K*. *pneumoniae*, fluroquinolone resistance in *E coli*, *K*. *pneumoniae*, and *S*. *aureus*, aminoglycoside resistance in *E*. *coli* and *K*. *pneumoniae*, and carbapenem resistance in *P*. *aeruginosa* (Section 5 in S2 Appendix). The difference in resistance prevalence for the latter is relatively stable at approximately −10% across countries, while others have large variability in magnitude across countries. There is also substantial variation in resistance prevalence between old and young (ages 100 versus 1) between different bacteria-antibiotic combinations within countries.

**Fig 5:**
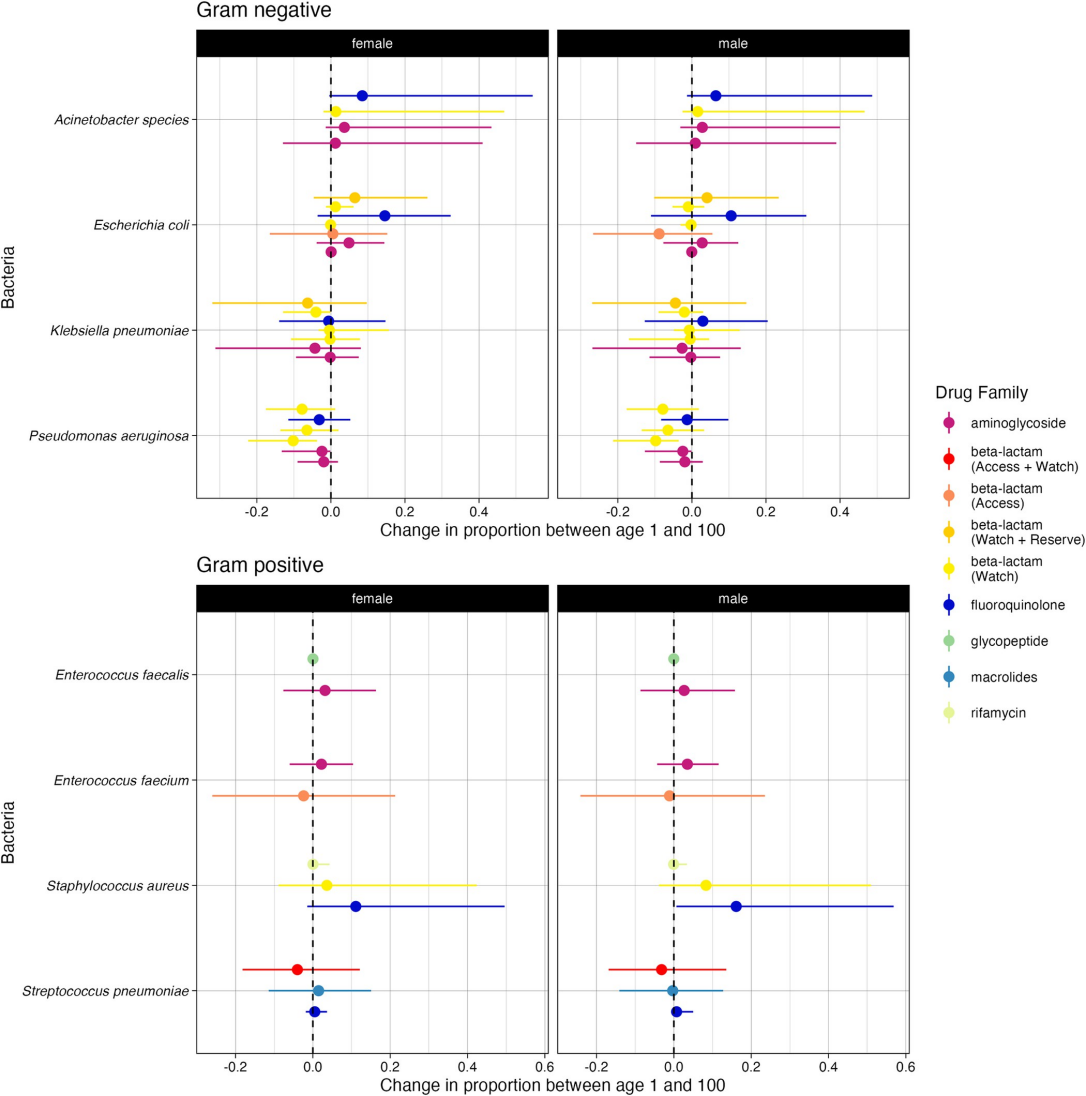
Change in model-predicted proportion of samples resistant between older (age 100) and younger (age 1) patients with BSIs. The index is split by sex (panels) and Gram stain test result. Each point indicates the median index for each individual antibiotic coloured by drug family with the error bars showing the 95% quantiles across model predictions. The dashed line indicates 0 (i.e., no difference in model-predicted difference between resistance prevalence at age 1 vs. 100). The anonymised country-level results can be seen in Section 5 in S2 Appendix. BSI, bloodstream infection.

Within bacterial species by comparing the model-predicted proportion resistant between old and young (ages 100 versus 1), age-related trends were seen across all antibiotics for *Acinetobacter* species (positive, both sexes), *E*. *coli* (positive, female), and *P*. *aeruginosa* (negative, both sexes) (Fig 5), although for these the majority were not significant at the 95% level, and have relatively small impacts. No clear age-related trends were seen in our modelling results within Gram stain groupings (Fig 5), nor within antibiotic families (colours in Fig 5), further emphasising the trends seen at the European level (Fig 1).

### Sensitivity analysis

Analysis of infection incidence by patient type and healthcare location when the sample was taken revealed large differences between countries across Europe likely linked to differences in healthcare systems and reporting protocols (Sections 7 and 8 in S2 Appendix). Hence, we could not here explore resistance prevalence further by these differences. Age and sex patterns in incidence were robust to using the minimum surveillance coverage values (Section 9 in S2 Appendix).

Modelling sensitivity analyses showed little effect from including regularising priors or of including samples from individuals aged 0 (Sections 10 and 11 in S2 Appendix). Our model selection sensitivity analysis showed that even with a different structural approach, the model used in the main analysis was the preferred model, with the age^2^ term being beneficial to model fit (Section 12 in S2 Appendix).

## Discussion

We find distinct patterns by age and sex in resistance prevalence in BSI across Europe. Surprisingly, resistance prevalence does not always increase with age nor is it higher in women, suggesting that antimicrobial exposure is not the sole driver of resistance. Instead, high spatial variation suggests that cultural factors play an important role, and differences between bacteria in terms of their natural history and pathways to infection are important to consider in tacking AMR. While there are limited previous studies looking at age and sex in the context of AMR, these do not provide their estimates of the relationships as output [21] or are limited to explorations in specific settings or bacterial species [18,27,53–55]. The only previous report considering age differences in detail focuses only on 6 bacteria-antibiotic combinations in 5-year age bands, and shows similar trends as we do [17] but did not quantify such differences nor explore them at the national, or subnational, level. The main strengths of our analyses lie in the detailed BSI data used, as we were able to use 1-year age bands, where trends may have been obscured in previous studies by high-level age groupings (e.g., [9,18,21,56]). However, we are limited by the nature of routinely available surveillance data. Also, our aim was to present the trends and hence we use a limited set of model structures. These new findings about differences by age and sex should now be considered in AMR research as they have the potential to yield new insights into AMR epidemiology and may inform the design of control measures.

Focusing on 2 critical pathogens for BSI (*E*. *coli* and *S*. *aureus*, isolated from *>*40% of BSI in a global survey) [51] and associated important resistances in Europe [45] and globally [52], we show substantial subnational and national variation, while demonstrating that there are age- and sex-related trends for specific bacteria-antibiotic combinations. Transmission of MRSA often occurs in healthcare settings [57] and increased contact with such settings with age could explain our observed often positive trend in resistance proportions by age. While for aminopenicillin resistance in *E*. *coli*, the contrasting dominant negative trend in resistance with age could be explained if, with age, more infections were endogenous and community-onset (see below). Exploring the contribution of community- versus hospital-associated infections (which we could not do with this data), as well as combinations of resistance within single isolates could test this hypothesis and explain country-level variation.

While resistance prevalence in many gram-negative bacteria often peaked at younger ages (as has been predicted for multidrug-resistant *M*. *tuberculosis* carriage [58]), we found little commonality in patterns between bacteria or within drug families. This suggests that the link between demographics and AMR is likely to be less driven by biological factors, but instead is driven more by cultural or behavioural factors, as alternatively we might expect similar patterns of resistance within drug families across different bacteria. Contrasting this with the biological factors that can drive increased infection risk by age suggests that there is vital information in comparing and contrasting AMR prevalence in infection spatially by age and sex to improve intervention design and antimicrobial usage. For example, understanding trends in resistance by age could lead to improved understanding of the importance of antimicrobial use variation between ages and countries [59], healthcare contact and infection prevention control practices, and even microbiological sampling that could inform both data analysis for burden and evolution understanding as well as transmission intervention potential. Comparing and contrasting antibiotic combinations used for important syndromes, such as community-acquired pneumonia, with the resistance patterns seen in common causative organisms could point to specific age-related selective pressures and hence targets for antibiotic stewardship.

Summarising the nonlinearity of the resistance prevalence patterns by age was challenging. The level of heterogeneity was reflected in the variation in the multilevel model coefficients estimated (Table 2) and in the country-level model analysis (Fig 3). However, we found that using a simplifying index, comparing model-predicted resistance prevalence in infections in younger versus older individuals (age 1 versus 100, but robust to other age comparisons), revealed broad trends across countries. For example, showing sex-related differences that were often consistent, if varying in magnitude, across countries but were not consistent across bacteria-antibiotic combinations. With non-anonymised country data, this could be used by international agencies to support specific countries to explore drivers and prioritise the age- and sex-related targeting of interventions.

Our results could inform policy and practice in healthcare settings in a variety of ways. Firstly, understanding differences in age- and sex-related risks of infection with resistant bacteria could lead to more targeted empiric prescribing, tailored to the individual and setting, as has previously been suggested [18,20,60]. This may be particularly important in older adults that often experience more severe consequences of bacterial infection [61]. There could also be potential for improved treatment in younger adults through recommendations for the use of antibiotics which are currently limited across adults due to concerns about serious risks from resistant infections (driven by higher risk profiles for older populations) or other age-related concerns such as quinolone use linked *C*. *difficile* infections. Aside from more targeted age-based, and potentially sex-based, antibiotic stewardship, this analysis points to interventions that target transmission and de-colonisation strategies. For example, knowledge of higher resistance prevalence in BSI in a certain age/sex group could lead to their being a prioritisation for de-colonisation strategies [62], as well as potential candidates for single room or personal protective equipment infection prevention control interventions to prevent onward transmission.

Secondly, understanding the importance of demographic factors on AMR will support the collection and smart use of further data in this area. This is especially relevant due to the high levels of variation across settings that we identify, underpinning the need for local-level infection data collection and policies. Only this level of data would enable determination of the local sources of AMR and hence optimal targeting of interventions. While demographic data is often encouraged to be reported by countries, this is often not included in analyses, and its use is confounded by differences in the data sources and sampling practices [63]. These differences, and the subnational variation we found, highlight the need for reducing the reliance on estimates of AMR based on either single settings within a country or national averages, as done with large global estimation studies [56], as averaging across data collected from different study sites can reduce the accuracy and poorly reflect heterogeneity.

This is not only true for understanding AMR, but also BSI risks—sex differences in incidence by age could give clues to targeting this large contributor to mortality that are not commonly explored or considered in BSI epidemiology [64]. The clear higher BSI rate in men, apart from for *E*. *coli* infections in those aged 15 to 40, contrasts with the lack of clear sex effect in many of the resistance trends. The higher BSI levels in women aged 15 to 40 has been seen previously [65,66] and could reflect the higher urinary tract infection incidence in women [67] which are a common BSI source [68,69] and potentially reflect hormonal changes that can affect the microbiome between menarche and menopause [70]. Future work is needed to quantify the contribution of biological sex versus sex-related exposure and societal influences on both the direct and indirect risks of infection and hence AMR.

In addition to the direct implications of our findings on public health policy, understanding of the links between demographics and AMR will be foundational to a deeper understanding of acquisition routes of AMR. In our analysis, we explored demographic trends across populations but are limited in our ability to understand the mechanisms behind these trends, where further research is required. One potential avenue for such research is to explore the primary source of the bacterium causing the BSI: endogenous following long-term carriage, with a potential minor infection prior to the BSI or recent transmission. Age- and sex-related patterns in BSI source will be influenced by many factors, such as levels of contact with healthcare systems (e.g., hospital stays, previous antibiotic prescriptions [60]), individual behaviour (e.g., causes for hospital admission, rate of contact with other individuals), and inherent biology (e.g., immunosenescence, likelihood of source being a urinary tract or wound infection), as well as varying by bacteria-antibiotic combination. While the contribution of some of these factors have direct links to incidence by age (e.g., immunosenescence contributes to higher sepsis incidence with age [71]), linking these factors to proportion resistance by age is more complicated.

One theory linking the proportion of resistant infections with age is that older individuals are more likely to have weaker immune systems, and therefore are more likely to develop infections due to bacteria they are already colonised with and then enter the healthcare setting, as compared to younger individuals that would be relatively more likely to acquire a resistant bacterium through a transmission event within a healthcare setting. This would have implications for the proportion resistant by demographic characteristics for given bacteria and could be linked to changes in the microbiome with age [72]. One of the most surprising species we detected patterns for was *Acinetobacter* species, with strong age-, sex-, and subregion-related trends. This could be explained by young men being more likely to attend hospital for trauma compared to women [73], so if for this demographic population the key route to BSIs is from wound infections due to bacteria (such as *Acinetobacter* spp. [74]) acquired in hospital, this could explain the differences we observed in resistant proportion. Differences in incidence and resistance proportion could also be explained by the demographics of those who travel to areas with higher prevalence of *Acinetobacter* species [75]. Key to understanding the influence of the various factors on AMR is detailed knowledge at an individual level, as well as information on community versus hospital acquisition and antibiotic exposure, which we were unable to determine in this study. However, there are indications that for many bacteria, hospital-acquired infections are likely to have higher resistance [27] so changing contact with healthcare would be an important avenue to explore. Exploiting this demographic link could also be used to tackle and determine the key drivers of known subregional variation in resistance prevalence [45]. Our work therefore highlights the need for future research on the mechanisms of age- and sex-related AMR trends. In order to achieve this individual patient-level data, linked across primary and secondary care will be essential.

The large variation we see in age-sex-country trends in resistance prevalence is likely driven by a complex array of factors from the important high variation in antibiotic usage [35], to healthcare delivery variation [76] which can contribute to variation in healthcare practices, some of which will have sex-related impacts such as obstetric interventions [77]. Disentangling these structural differences from individual-level factors such as recent international travel [75], immune status, and comorbidities will require more detailed linked patient-level data as well as mathematical model methodology to simulate and test hypotheses as to the interaction and directionality of effect. Currently, the evidence as to the relative importance of these factors to driving the age-sex relative national differences is scarce.

Our research has several limitations. Firstly, we were unable to account for comorbidities and other syndromes of individuals, which may impact the age groups that are susceptible to different infections. For example, cystic fibrosis patients are known to be particularly susceptible to infections of *P*. *aeruginosa* [78], while also being correlated with the demographics of patients [79]. Not including such aspects may particularly bias our work on the future burden of AMR, as the demographics of the syndromes will likely also change over time [79]. This also highlights the need to take syndromes into account when prescribing antibiotics, as well as demographic factors, and to record such information alongside AMR data.

Our analysis is only of European data, and not split by community or hospital-onset, and as such may not represent universal trends that could vary in other settings, in particular where demographic and healthcare distributions are substantially different. Our interpretation and results are also limited by the anonymous nature of the country-linked information. We could not report non-anonymised country-level differences at individual age and by sex as this level of detail is not publicly available—only resistant proportion by gender and separately for age groups 0 to 4, 5 to 18, 19 to 64, and 65+ [9]. However, reporting only the European-level association would mask many patterns and hence we chose to show the results by anonymised country-level to emphasise the importance of continuing this analysis with country-specific data in the future.

In addition, the individuals included in this data set may be biassed because of variation in whose samples are sent to be tested for resistance—we are relying on data from routine surveillance. This variation will depend on demographics and can be influenced by the age of the individual, the severity of infection and previous failed antibiotic use, and testing guidelines, among other factors. Understanding the decisions in sampling made by clinicians and other healthcare professionals is a vital area of future study and may account for some of the local-level variation we have identified [63]. Upstream of the sampling decision, it may also be influenced by healthcare seeking behaviour, for example, women are more likely to seek healthcare than men [80], and this also varies by age and potentially by country. Variation in where within hospital settings samples are taken (ICU, A&E, etc.) may also explain some of the national and subnational variation we observe, but need further country-specific information to explore. Differences in sampling and processing may both create and obscure differences in resistance prevalence between populations. By using data from TESSy, which contains only blood and cerebrospinal fluid isolates, likely representing the most serious types of bacterial infection [81] where the vast majority of infections will be hospitalised, these biases should be minimal. However, this does not mean they are all sampled, and of those many samples will test negatively for infection [82]. The EARS-NET dataset also does not include all bacteria and all antibiotics and hence we have a limited, though clinically important, set of combinations considered.

In terms of the analysis and modelling in this paper, we chose to limit ourselves to a “one-size-fits-all” approach, applying the same models to each bacteria-antibiotic combination. There is potential for models that are a better fit to the data for specific bacteria-antibiotic combinations; however, this approach allowed us to compare model outputs across bacteria-antibiotic combinations, as well as reducing the complexity required. Lastly, we did not attempt to link AMR prevalence with mortality rates. This is because we did not have appropriate information to do this, with age-specific mortality rates and the impact of resistance on infection being hard to estimate, with variations in baselines used (e.g., associated versus attributable [56]). Recent estimates have found that data scarcity makes estimating relative risks of mortality by subgroups or geographical setting difficult [21].

Future work estimating the burden of AMR and impact of interventions will need to account for these trends by age and sex to accurately capture burden. The complexity in trends in resistance prevalence by age and sex interact with the exponential increase in BSI incidence with age to mean that often, the elderly population, especially men, would still be expected to suffer more infections with resistant bacteria. How this collides with the global shift to older populations [2] and the impact this will have on public health burden as well as AMR spread should be a research priority. Future work is also needed to explore the national and subnational variation using age- and sex-disaggregated antibiotic usage data which is not currently widely available, as well as other national policy differences in infection control, antibiotic stewardship, and demographic trends. Fundamental differences in healthcare contact by sex related to pregnancy and chronic disease patterns also need to be explored in relation to driving both selection and transmission differences in resistance prevalence.

In 2018, the WHO asked “Is the impact of AMR the same for everyone? Do any groups in society face greater or different risks of exposure to AMR or more challenges in accessing, using and benefiting from the information, services and solutions to tackle AMR? If yes, who, why, and what can we do about it?” [30]. In this paper, we go some way to addressing these questions by quantifying how AMR prevalence in BSIs across Europe varies by age and sex, as well as identifying variation at the local level. We do not wish to oversimplify any trends in AMR by age or sex—risk factors, previous prescribing as well as contact with high-risk transmission settings such as hospital or long-term care facilities will all influence individual-level risk of AMR infection. However, our ecological analysis shows the substantial interactions of age and sex with AMR, and we therefore encourage their inclusion in future data collection and research studies to improve health outcomes across the spectrum of AMR.

## Supporting information

S1 Appendix Additional methods. (DOCX)

S2 Appendix Additional results and sensitivity analysis. (DOCX)

S3 Appendix Bacteria-antibiotic-specific model results. (PDF)

## Abbreviations

AMR: antimicrobial resistance
AST: antimicrobial susceptibility testing
BSI: bloodstream infection
COVID-19: Coronavirus Disease 2019
DRI: drug-resistant infection
EARS-Net: European Antimicrobial Resistance Surveillance Network
ECDC: European Centre for Disease Prevention and Control
MRSA: methicillin-resistant *Staphylococcus aureus*
WHO: World Health Organisation

## References

[1] WHO. Antibiotic resistance [Internet]. 2021 [cited 2021 Apr 1]. Available from: https://www.who.int/news-room/fact-sheets/detail/antibiotic-resistance.

[2] WHO. Ageing and health [Internet]. 2021 [cited 2021 Mar 23]. Available from: https://www.who.int/news-room/fact-sheets/detail/ageing-and-health.

[3] Gavazzi G, Krause KH. Ageing and infection. Lancet Infect Dis. 2002 Nov;2(11):659–66. 12409046 10.1016/s1473-3099(02)00437-1

[4] Dias SP, Brouwer MC, van de Beek D. Sex and Gender Differences in Bacterial Infections. Infect Immun. 2022 Oct 20;90(10):e0028322. doi: 10.1128/iai.00283-22 36121220 PMC9584217

[5] Prince MJ, Wu F, Guo Y, Gutierrez Robledo LM, O’Donnell M, Sullivan R, et al. The burden of disease in older people and implications for health policy and practice. Lancet Lond Engl. 2015 Feb 7;385(9967):549–62. doi: 10.1016/S0140-6736(14)61347-7 25468153

[6] Sadighi Akha AA. Aging and the immune system: An overview. J Immunol Methods. 2018 Dec;463:21–6. doi: 10.1016/j.jim.2018.08.005 30114401

[7] WHO. WHO. World Health Organization. 2020 [cited 2020 May 26]. GLASS | Global Antimicrobial Resistance Surveillance System (GLASS). Available from: http://www.who.int/glass/en/.

[8] WHO. WHO GLASS Dashboard [Internet]. 2023 [cited 2023 Feb 23]. Available from: https://worldhealthorg.shinyapps.io/glass-dashboard/_w_ef0f2081/#!/.

[9] European Centre for Disease Control and Prevention. Surveillance Atlas of Infectious Diseases [Internet]. 2023 [cited 2023 Feb 23]. Available from: https://atlas.ecdc.europa.eu/public/index.aspx?Dataset=27&HealthTopic=4.

[10] Centres For Disease Control and Prevention. Antibiotic Resistance | A.R. & Patient Safety Portal. 2023. Available from: https://arpsp.cdc.gov/profile/antibiotic-resistance?tab=antibiotic-resistance.

[11] WHO Regional Office for Europe, Europe/European Centre for Disease Prevention and Control. Antimicrobial resistance surveillance in Europe 2022–2020 data. Copenhagen: WHO Regional Office for Europe; 2022. Available from: https://www.ecdc.europa.eu/sites/default/files/documents/Joint-WHO-ECDC-AMR-report-2022.pdf.

[12] Kontula KSK, Skogberg K, Ollgren J, Järvinen A, Lyytikäinen O. Population-Based Study of Bloodstream Infection Incidence and Mortality Rates, Finland, 2004–2018. Emerg Infect Dis. 2021 Oct;27(10). doi: 10.3201/eid2710.204826 34546161 PMC8462341

[13] Gagliotti C, Högberg LD, Billström H, Eckmanns T, Giske CG, Heuer OE, et al. Staphylococcus aureus bloodstream infections: diverging trends of meticillin-resistant and meticillin-susceptible isolates, EU/EEA, 2005 to 2018. Eur Secur. 2021 Nov 18;26(46):2002094. doi: 10.2807/1560-7917.ES.2021.26.46.2002094 34794536 PMC8603406

[14] Thorlacius-Ussing L, Sandholdt H, Larsen AR, Petersen A, Benfield T. Age-Dependent Increase in Incidence of Staphylococcus aureus Bacteremia, Denmark, 2008–2015. Emerg Infect Dis. 2019 May;25(5):875–82. doi: 10.3201/eid2505.181733 31002300 PMC6478196

[15] Wilson J, Elgohari S, Livermore DM, Cookson B, Johnson A, Lamagni T, et al. Trends among pathogens reported as causing bacteraemia in England, 2004–2008. Clin Microbiol Infect. 2011 Mar 1;17(3):451–8. doi: 10.1111/j.1469-0691.2010.03262.x 20491834

[16] Laupland KB, Lyytikäinen O, Søgaard M, Kennedy KJ, Knudsen JD, Ostergaard C, et al. The changing epidemiology of Staphylococcus aureus bloodstream infection: a multinational population-based surveillance study. Clin Microbiol Infect. 2013 May;19(5):465–71. doi: 10.1111/j.1469-0691.2012.03903.x 22616816

[17] OECD. Stemming the Superbug Tide: Just A Few Dollars More. Paris: OECD Publishing; 2018. Available from: https://www.oecd.org/health/stemming-the-superbug-tide-9789264307599-en.htm.

[18] Weber SG, Miller RR, Perencevich EN, Tolentino J, Meltzer D, Pitrak D, et al. Prevalence of antimicrobial-resistant bacteria isolated from older versus younger hospitalized adults: results of a two-centre study. J Antimicrob Chemother. 2009 Dec;64(6):1291–8. doi: 10.1093/jac/dkp349 19808237 PMC2775664

[19] Sivasankar S, Goldman JL, Hoffman MA. Variation in antibiotic resistance patterns for children and adults treated at 166 non-affiliated US facilities using EHR data. JAC-Antimicrob Resist. 2023 Feb;5(1):dlac128. doi: 10.1093/jacamr/dlac128 36601546 PMC9806600

[20] Swami SK, Banerjee R. Comparison of hospital-wide and age and location—stratified antibiograms of S. aureus, E. coli, and S. pneumoniae: age- and location-stratified antibiograms. SpringerPlus. 2013 Dec;2(1):63. doi: 10.1186/2193-1801-2-63 23487499 PMC3593003

[21] Mestrovic T, Aguilar GR, Swetschinski LR, Ikuta KS, Gray AP, Weaver ND, et al. The burden of bacterial antimicrobial resistance in the WHO European region in 2019: a cross-country systematic analysis. Lancet Public Health. 2022 Nov 1;7(11):e897–913. doi: 10.1016/S2468-2667(22)00225-0 36244350 PMC9630253

[22] Glynn JR, Moss PAH. Systematic analysis of infectious disease outcomes by age shows lowest severity in school-age children. Sci Data. 2020 Oct 15;7(1):329. doi: 10.1038/s41597-020-00668-y 33057040 PMC7566589

[23] ReAct. ReAct Report: Antibiotic resistance affects men and women differently– 2020. Available from: https://www.reactgroup.org/news-and-views/news-and-opinions/year-2020/new-react-report-antibiotic-resistance-affects-men-and-women-differently/.

[24] Badura A, Feierl G, Pregartner G, Krause R, Grisold AJ. Antibiotic resistance patterns of more than 120 000 clinical Escherichia coli isolates in Southeast Austria, 1998–2013. Clin Microbiol Infect Off Publ Eur Soc Clin Microbiol Infect Dis. 2015 Jun;21(6):569.e1–7. 10.1016/j.cmi.2015.02.012 25704445

[25] Badura A, Pregartner G, Holzer JC, Feierl G, Grisold AJ. Susceptibility of Austrian Clinical Klebsiella and Enterobacter Isolates Linked to Patient-Related Data. Front Microbiol. 2016;7:34. doi: 10.3389/fmicb.2016.00034 26903953 PMC4743402

[26] Livermore DM, Nichols T, Lamagni TL, Potz N, Reynolds R, Duckworth G. Ciprofloxacin-resistant Escherichia coli from bacteraemias in England; increasingly prevalent and mostly from men. J Antimicrob Chemother. 2003 Dec 1;52(6):1040–2. doi: 10.1093/jac/dkg479 14585850

[27] Sahuquillo-Arce JM, Selva M, Perpiñán H, Gobernado M, Armero C, López-Quílez A, et al. Antimicrobial Resistance in More than 100,000 Escherichia coli Isolates According to Culture Site and Patient Age, Gender, and Location. Antimicrob Agents Chemother. 2011 Mar;55(3):1222–8. doi: 10.1128/AAC.00765-10 21220537 PMC3067086

[28] Nguyen HQ, Nguyen NTQ, Hughes CM, O’Neill C. Trends and impact of antimicrobial resistance on older inpatients with urinary tract infections (UTIs): A national retrospective observational study. PLoS ONE. 2019;14(10):e0223409. doi: 10.1371/journal.pone.0223409 31581222 PMC6776395

[29] MacKinnon MC, McEwen SA, Pearl DL, Lyytikäinen O, Jacobsson G, Collignon P, et al. Increasing incidence and antimicrobial resistance in Escherichia coli bloodstream infections: a multinational population-based cohort study. Antimicrob Resist Infect Control. 2021 Sep 6;10(1):131. doi: 10.1186/s13756-021-00999-4 34488891 PMC8422618

[30] WHO. TACKLING ANTIMICROBIAL RESISTANCE (AMR) TOGETHER Working Paper 5.0: Enhancing the focus on gender and equity. 2018. Available from: https://www.who.int/antimicrobial-resistance/national-action-plans/AMRGenderEquityGuidance-Sept2018.pdf.

[31] Chua AQ, Verma M, Hsu LY, Legido-Quigley H. An analysis of national action plans on antimicrobial resistance in Southeast Asia using a governance framework approach. Lancet Reg Health West Pac. 2021 Feb;7:100084. doi: 10.1016/j.lanwpc.2020.100084 34327414 PMC8315476

[32] Thomson K, Berry R, Robinson T, Brown H, Bambra C, Todd A. An examination of trends in antibiotic prescribing in primary care and the association with area-level deprivation in England. BMC Public Health. 2020 Aug 3;20:1148. doi: 10.1186/s12889-020-09227-x 32741362 PMC7397662

[33] Collignon P, Beggs JJ, Walsh TR, Gandra S, Laxminarayan R. Anthropological and socioeconomic factors contributing to global antimicrobial resistance: a univariate and multivariable analysis. Lancet Planet Health. 2018;2(9):e398–e405. doi: 10.1016/S2542-5196(18)30186-4 30177008

[34] Auguet OT, Betley JR, Stabler RA, Patel A, Ioannou A, Marbach H, et al. Evidence for Community Transmission of Community-Associated but Not Health-Care-Associated Methicillin-Resistant Staphylococcus Aureus Strains Linked to Social and Material Deprivation: Spatial Analysis of Cross-sectional Data. PLoS Med. 2016 Jan 26;13(1):e1001944. doi: 10.1371/journal.pmed.1001944 26812054 PMC4727805

[35] European Centre for Disease Prevention and Control. Antimicrobial consumption in the EU/EEA (ESAC-Net)—Annual Epidemiological Report for 2021. Stockholm: ECDC; 2022 Nov. Available from: https://www.ecdc.europa.eu/en/publications-data/surveillance-antimicrobial-consumption-europe-2021.

[36] Curtis HJ, Walker AJ, Mahtani KR, Goldacre B. Time trends and geographical variation in prescribing of antibiotics in England 1998–2017. J Antimicrob Chemother. 2019 Jan 1;74(1):242–50. doi: 10.1093/jac/dky377 30239809

[37] Eurostat. Fertility statistics [Internet]. 2022 [cited 2023 Feb 24]. Available from: https://ec.europa.eu/eurostat/statistics-explained/index.php?title=Fertility_statistics.

[38] Eurostat. Surgical operations and procedures statistics. 2023 [cited 2023 Feb 24]. Available from: https://ec.europa.eu/eurostat/statistics-explained/index.php?title=Surgical_operations_and_procedures_statistics.

[39] European Centre for Disease Prevention and Control. The European Surveillance System (TESSy). 2022. Available from: https://www.ecdc.europa.eu/en/publications-data/european-surveillance-system-tessy.

[40] European Centre for Disease Prevention and Control. EARS-Net Reporting protocol. 2022. Available from: https://www.ecdc.europa.eu/en/publications-data/ears-net-reporting-protocol-2022.

[41] European Centre for Disease Prevention and Control. External quality assessment of laboratory performance-European Antimicrobial Resistance Surveillance Network (EARS-Net). 2018. Available from: https://www.ecdc.europa.eu/en/publications-data/external-quality-assessment-laboratoryperformance-european-antimicrobial-0.

[42] European Committee on Antimicrobial Susceptibility Testing (EUCAST). EUCAST guidelines for detection of resistance mechanisms and specific resistances of clinical and/or epidemiological importance. Version 2 [Internet]. Växjö, EUCAST; 2017. Available from: http://www.eucast.org/fileadmin/src/media/PDFs/EUCAST_files/Resistance_mechanisms/EUCAST_detection_of_resistance_mechanisms_170711.pdf.

[43] Niewiesk S. Maternal Antibodies: Clinical Significance, Mechanism of Interference with Immune Responses, and Possible Vaccination Strategies. Front Immunol. 2014 Sep 16;5:446. doi: 10.3389/fimmu.2014.00446 25278941 PMC4165321

[44] Simon AK, Hollander GA, McMichael A. Evolution of the immune system in humans from infancy to old age. Proc R Soc B Biol Sci. 2015 Dec 22;282(1821):20143085. doi: 10.1098/rspb.2014.3085 26702035 PMC4707740

[45] Central Asian and European surveillance of Antimicrobial Resistance (CAESAR), European Antimicrobial Resistance Surveillance Network (EARS-Net). Antimicrobial resistance surveillance in Europe—2023. 2023 Apr 14 [cited 2023 Apr 26]. Available from: https://www.ecdc.europa.eu/en/publications-data/antimicrobial-resistance-surveillance-europe-2023.

[46] Sharland M, Cappello B, Ombajo LA, Bazira J, Chitatanga R, Chuki P, et al. The WHO AWaRe Antibiotic Book: providing guidance on optimal use and informing policy. Lancet Infect Dis. 2022 Nov 1;22(11):1528–30. doi: 10.1016/S1473-3099(22)00683-1 36309019

[47] Cassini A, Högberg LD, Plachouras D, Quattrocchi A, Hoxha A, Simonsen GS, et al. Attributable deaths and disability-adjusted life-years caused by infections with antibiotic-resistant bacteria in the EU and the European Economic Area in 2015: a population-level modelling analysis. Lancet Infect Dis. 2019 Jan 1;19(1):56–66. doi: 10.1016/S1473-3099(18)30605-4 30409683 PMC6300481

[48] The World Bank Group. DataBank. 2022 [cited 2022 Oct 19]. Available from: https://databank.worldbank.org/home.

[49] Bürkner PC. brms: An R Package for Bayesian Multilevel Models Using Stan. J Stat Softw. 2017 Aug 29;80:1–28.

[50] Brauer R, Ruigómez A, Downey G, Bate A, Garcia Rodriguez LA, Huerta C, et al. Prevalence of antibiotic use: a comparison across various European health care data sources. Pharmacoepidemiol Drug Saf. 2016 Mar;25 Suppl 1(Suppl Suppl 1):11–20. doi: 10.1002/pds.3831 26152658 PMC4918309

[51] Diekema DJ, Hsueh PR, Mendes RE, Pfaller MA, Rolston KV, Sader HS, et al. The Microbiology of Bloodstream Infection: 20-Year Trends from the SENTRY Antimicrobial Surveillance Program. Antimicrob Agents Chemother. 2019 Jun 24;63(7):e00355–19. doi: 10.1128/AAC.00355-19 31010862 PMC6591610

[52] Tacconelli E, Carrara E, Savoldi A, Harbarth S, Mendelson M, Monnet DL, et al. Discovery, research, and development of new antibiotics: the WHO priority list of antibiotic-resistant bacteria and tuberculosis. Lancet Infect Dis. 2018 Mar;18(3):318–27. doi: 10.1016/S1473-3099(17)30753-3 29276051

[53] Garcia A, Delorme T, Nasr P. Patient age as a factor of antibiotic resistance in methicillin-resistant Staphylococcus aureus. J Med Microbiol. 2017 Dec;66(12):1782–9. doi: 10.1099/jmm.0.000635 29116037

[54] Boyd LB, Atmar RL, Randall GL, Hamill RJ, Steffen D, Zechiedrich L. Increased fluoroquinolone resistance with time in Escherichia coli from >17,000 patients at a large county hospital as a function of culture site, age, sex, and location. BMC Infect Dis. 2008 Jan 15;8:4. 18197977 10.1186/1471-2334-8-4 PMC2258293

[55] Ji Z, Han F, Meng F, Tu M, Yang N, Zhang J. The Association of Age and Antibiotic Resistance of Helicobacter Pylori. Medicine (Baltimore). 2016 Mar 3;95(8). 10.1097/MD.0000000000002831 PMC4779009 26937912

[56] Murray CJ, Ikuta KS, Sharara F, Swetschinski L, Aguilar GR, Gray A, et al. Global burden of bacterial antimicrobial resistance in 2019: a systematic analysis. Lancet [Internet]. 2022 Jan 19 [cited 2022 Jan 25];0(0). 10.1016/S0140-6736(21)02724-0 PMC8841637 35065702

[57] Turner NA, Sharma-Kuinkel BK, Maskarinec SA, Eichenberger EM, Shah PP, Carugati M, et al. Methicillin-resistant Staphylococcus aureus: an overview of basic and clinical research. Nat Rev Microbiol. 2019 Apr;17(4):203–18. doi: 10.1038/s41579-018-0147-4 30737488 PMC6939889

[58] Knight GM, McQuaid CF, Dodd PJ, Houben RMGJ. Global burden of latent multidrug-resistant tuberculosis: trends and estimates based on mathematical modelling. Lancet Infect Dis. 2019 Aug;19(8):903–12. doi: 10.1016/S1473-3099(19)30307-X 31281059 PMC6656782

[59] Mor A, Frøslev T, Thomsen RW, Oteri A, Rijnbeek P, Schink T, et al. Antibiotic use varies substantially among adults: a cross-national study from five European Countries in the ARITMO project. Infection. 2015 Aug;43(4):453–72. doi: 10.1007/s15010-015-0768-8 25828936

[60] Yelin I, Snitser O, Novich G, Katz R, Tal O, Parizade M, et al. Personal clinical history predicts antibiotic resistance of urinary tract infections. Nat Med. 2019 Jul;25(7):1143–52. doi: 10.1038/s41591-019-0503-6 31273328 PMC6962525

[61] Esme M, Topeli A, Yavuz BB, Akova M. Infections in the Elderly Critically-Ill Patients. Front Med. 2019 Jun 6;6:118. doi: 10.3389/fmed.2019.00118 31275937 PMC6593279

[62] Roson-Calero N, Ballesté-Delpierre C, Fernández J, Vila J. Insights on Current Strategies to Decolonize the Gut from Multidrug-Resistant Bacteria: Pros and Cons. Antibiotics. 2023 Jun 19;12(6):1074. doi: 10.3390/antibiotics12061074 37370393 PMC10295446

[63] WHO. Methodological principles of nationally representative surveys as a platform for global surveillance of antimicrobial resistance in human bloodstream infection. Geneva: World Health Organization; 2023. Licence: CC BY-NC-SA 3.0 IGO.

[64] Kern WV, Rieg S. Burden of bacterial bloodstream infection-a brief update on epidemiology and significance of multidrug-resistant pathogens. Clin Microbiol Infect. 2020 Feb;26(2):151–7. doi: 10.1016/j.cmi.2019.10.031 31712069

[65] UK Health Security Agency. Enterococcus spp. bacteraemia: voluntary surveillance 2021–2023. Available from: https://www.gov.uk/government/publications/enterococcus-spp-bacteraemia-voluntary-surveillance-2021.

[66] Bonten M, Johnson JR, van den Biggelaar AHJ, Georgalis L, Geurtsen J, de Palacios PI, et al. Epidemiology of Escherichia coli Bacteremia: A Systematic Literature Review. Clin Infect Dis. 2021 Apr 1;72(7):1211–9. doi: 10.1093/cid/ciaa210 32406495

[67] Al-Badr A, Al-Shaikh G. Recurrent Urinary Tract Infections Management in Women. Sultan Qaboos Univ Med J. 2013 Aug;13(3):359–67. 23984019 10.12816/0003256 PMC3749018

[68] Abernethy J, Guy R, Sheridan EA, Hopkins S, Kiernan M, Wilcox MH, et al. Epidemiology of Escherichia coli bacteraemia in England: results of an enhanced sentinel surveillance programme. J Hosp Infect. 2017 Apr;95(4):365–75. doi: 10.1016/j.jhin.2016.12.008 28190700

[69] UK Health Security Agency. Annual epidemiological commentary: Gram-negative, MRSA, MSSA bacteraemia and C. difficile infections, up to and including financial year 2021 to 2022. Available from: https://www.gov.uk/government/statistics/mrsa-mssa-and-e-coli-bacteraemia-and-c-difficile-infection-annual-epidemiological-commentary/annual-epidemiological-commentary-gram-negative-mrsa-mssa-bacteraemia-and-c-difficile-infections-up-to-and-including-financial-year-2021-to-2022.

[70] Auriemma RS, Scairati R, Del Vecchio G, Liccardi A, Verde N, Pirchio R, et al. The Vaginal Microbiome: A Long Urogenital Colonization Throughout Woman Life. Front Cell Infect Microbiol. 2021;11:686167. doi: 10.3389/fcimb.2021.686167 34295836 PMC8290858

[71] Girard TD, Ely EW. Bacteremia and sepsis in older adults. Clin Geriatr Med. 2007 Aug;23(3):633–647. viii. doi: 10.1016/j.cger.2007.05.003 17631238

[72] Bosco N, Noti M. The aging gut microbiome and its impact on host immunity. Genes Immun. 2021 Oct;22(5):289–303. doi: 10.1038/s41435-021-00126-8 33875817 PMC8054695

[73] NHS. NHS Digital. 2021 [cited 2023 Jun 21]. Hospital Admitted Patient Care Activity. Available from: https://digital.nhs.uk/data-and-information/publications/statistical/hospital-admitted-patient-care-activity.

[74] Chopra T, Marchaim D, Awali RA, Krishna A, Johnson P, Tansek R, et al. Epidemiology of Bloodstream Infections Caused by Acinetobacter baumannii and Impact of Drug Resistance to both Carbapenems and Ampicillin-Sulbactam on Clinical Outcomes. Antimicrob Agents Chemother. 2013 Dec;57(12):6270–5. doi: 10.1128/AAC.01520-13 24100492 PMC3837851

[75] Bokhary H, Pangesti KNA, Rashid H, Abd El Ghany M, Hill-Cawthorne GA. Travel-Related Antimicrobial Resistance: A Systematic Review. Trop Med Infect Dis Ther. 2021 Jan 16;6(1):11. doi: 10.3390/tropicalmed6010011 33467065 PMC7838817

[76] Peiró S, Maynard A. Variations in health care delivery within the European Union. Eur J Pub Health. 2015 Feb 1;25(suppl_1):1–2. 10.1093/eurpub/cku223 25690122

[77] Macfarlane AJ, Blondel B, Mohangoo AD, Cuttini M, Nijhuis J, Novak Z, et al. Wide differences in mode of delivery within Europe: risk-stratified analyses of aggregated routine data from the Euro-Peristat study. BJOG Int J Obstet Gynaecol. 2016 Mar;123(4):559–68. 10.1111/1471-0528.13284 25753683

[78] Lyczak JB, Cannon CL, Pier GB. Lung Infections Associated with Cystic Fibrosis. Clin Microbiol Rev. 2002 Apr;15(2):194–222. 11932230 10.1128/CMR.15.2.194-222.2002 PMC118069

[79] Keogh RH, Tanner K, Simmonds NJ, Bilton D. The changing demography of the cystic fibrosis population: forecasting future numbers of adults in the UK. Sci Rep. 2020 Jun 30;10(1):10660. doi: 10.1038/s41598-020-67353-3 32606329 PMC7327064

[80] Thompson AE, Anisimowicz Y, Miedema B, Hogg W, Wodchis WP, Aubrey-Bassler K. The influence of gender and other patient characteristics on health care-seeking behaviour: a QUALICOPC study. BMC Fam Pract. 2016 Dec;17(1):1–7. doi: 10.1186/s12875-016-0440-0 27036116 PMC4815064

[81] Goto M, Al-Hasan MN. Overall burden of bloodstream infection and nosocomial bloodstream infection in North America and Europe. Clin Microbiol Infect. 2013 Jun;19(6):501–9. doi: 10.1111/1469-0691.12195 23473333

[82] Ratzinger F, Dedeyan M, Rammerstorfer M, Perkmann T, Burgmann H, Makristathis A, et al. A risk prediction model for screening bacteremic patients: a cross sectional study. PLoS ONE. 2014;9(9):e106765. doi: 10.1371/journal.pone.0106765 25184209 PMC4153716

