# Supplementary material for: Antimicrobial resistance prevalence in bloodstream infection in 29 European countries by age and sex: An observational study
Source: PLoS Med. 2024 Mar 14;21(3):e1004301. doi: 10.1371/journal.pmed.1004301 (PMC10939247; doi:10.1371/journal.pmed.1004301)
Supplement: S1 Appendix — (DOCX) [file pmed.1004301.s001.docx]

**S1 Appendix:** **Additional methods for “Antimicrobial resistance prevalence in bloodstream infection in 29 European countries by age and sex: an observational study”**

Table of Contents

[1. Abbreviations 2](#_Toc157409104)

[2. Data cleaning 3](#_Toc157409105)

[3. Incidence calculations 11](#_Toc157409106)

[4. Population trends – 2000 to 2021 14](#_Toc157409107)

[5. Model fitting 15](#_Toc157409108)

## Abbreviations

ACISPP = *Acinetobacter spp*

ENCFAE = *Enterococcus faecalis*

ENCFAI = *Enterococcus faecium*

ESCCOL = *Escherichia coli*

KLEPNE = *Klebsiella pneumoniae*

PSEAER = *Pseudomonas aeruginosa*

STAAUR = *Staphylococcus aureus*

STRPNE = *Streptococcus pneumoniae*

#K = number of thousands

AMR = antimicrobial resistance

BSI = bloodstream infection

EARS-NET = European Antimicrobial Resistance Surveillance Network (EARS-Net)

ECDC = European Centre for Disease Prevention and Control

GLASS = Global Antimicrobial Resistance and Use Surveillance System

IQR = interquartile range

MRSA = methicillin resistant *S. aureus*

WHO = World Health Organisation

## Data cleaning

***Original data***

The original data from the ECDC included 3,549,617 isolates with age, reporting country and different combinations of test results to a variety of antibiotics. This grouped into 510,816 unique combinations of year, age, pathogen, gender, antibiotic tested and country with a total of 9,855,100 antibiotic testing results (either susceptible “0” or resistant “1”) over 46 bacteria-antibiotic groupings. Samples were tested to a different combination of antibiotics: on average 2.7 antibiotic test results were conducted per sample. There were slightly fewer females in the data: 1,445,370 (45%) of isolates were from females.

The proportion of isolates in each age group (0-4, 5-19, 20-64, 65+) and by patient sex are reported in EARS-Net country summaries and on the ECDC Dashboard (e.g. of the 2019 report [1]).

***Data overview***

1. Laboratory code: There were 1755 unique laboratory codes in the data with a wide range of number of samples per laboratory: an average of 1803 [IQR: 47 - 2202]. No specimens were missing a laboratory code.
2. Antibiotics: Across the 24 different antibiotic resistance groupings (e.g. some to individual antibiotics, some to families such as macrolides, some to multiple resistance) over the whole dataset, most had more than 50,000 results (susceptible or resistant value), but three were outliers with fewer than 20,000.
3. Some antibiotic classifications were not to single antibiotics but were summary indicators of “multiple resistances” i.e. in the data a `1` indicated multi-resistance when resistance to a set combination of resistances was measured e.g. to macrolides and penicillin. This data was excluded as the aim here was to consider resistance to single antibiotics, not combinations. These ”combined” resistance profiles were generated to look at levels of multi-resistant bacteria in the EARS-Net analysis (e.g. Table 3 in [1]).
4. Bacteria: Of the 7 bacteria in the dataset, all had >150,000 results apart from *Acinetobacter* species which had ~50,000. All bacteria were kept in the data.
5. Countries: Across countries, four countries had approximately a million isolates. There was a substantial decline across the other countries. 5 countries had fewer than 12,000 isolates. We did not exclude any country from the analysis.
6. Years: Across the 19 years (2002 – 2020), the earlier time points had fewer data (~65K results) compared to more than ~750K from 2016. This was distributed differently across different pathogens and so we did not explicitly exclude certain years. Instead, we focused our analysis on the final five years prior to the COVID-19 pandemic i.e. 2015-2019. Subsetting the data to this time period only removed the country Lithuania which had no linked gender data for this time period.

***Antibiotic resistance definitions***

For the fluoroquinolones there was a different indicator (Table A1) for different species:

- fq_pseudo_R for *P. aeruginosa* and *Acinetobacter*
- fq_ent_R for *E. coli*  and *K. pneumoniae*
- fq_staaur_R for *S. aureus*
- fq_strpne_R for *S. pneumoniae*

As we fit to data for each bacteria-antibiotic separately, we label these the same as fluroquinolone resistance but they have strain specific definitions.

For the aminoglycosides we were provided with data for “amikacin” and “high-level gentamicin” resistance separately to an “aminoglycoside” resistance category. We provide the results for each of these separately.

MRSA primarily indicates oxacillin or cefoxitin resistance, but other markers are accepted for oxacillin, if oxacillin was not reported. See protocol for details [2].

***Missing data distributions***

To determine if the missingness in the data was completely at random, we investigated the missingness by key variables (age, sex and susceptibility). We considered the pattern overall as we’d expect different denominator patterns for each bacteria-antibiotic, but also explored the proportion of missingness for each bacterial species and for the case study of *S. aureus*.

Over the whole dataset, there were a total of 1,902,271 individual records over the 2015-2019 time period, of which 6.0% were missing age only, 1.5% were missing sex only and 2.2% were missing both age and sex. To check for biases in missingness we compared the distribution of ages with those missing sex and those not missing sex (Figure 1A). We also compared the distribution of sex with those missing and not missing age (Figure 1B). In both cases differences were minimal. As a case study, we ran the same analysis on just the *S. aureus* patients (Figure 2). To further confirm the minimality of differences a logistic regression was performed on those with missing gender information compared to those without missing information on the *S. aureus* subset, however these models did not reach convergence. This was also the case when looking at missingness in age. Therefore, as we could not investigate this further and the crude comparison showed minimal differences by missingness, imputation of missing data was not performed.


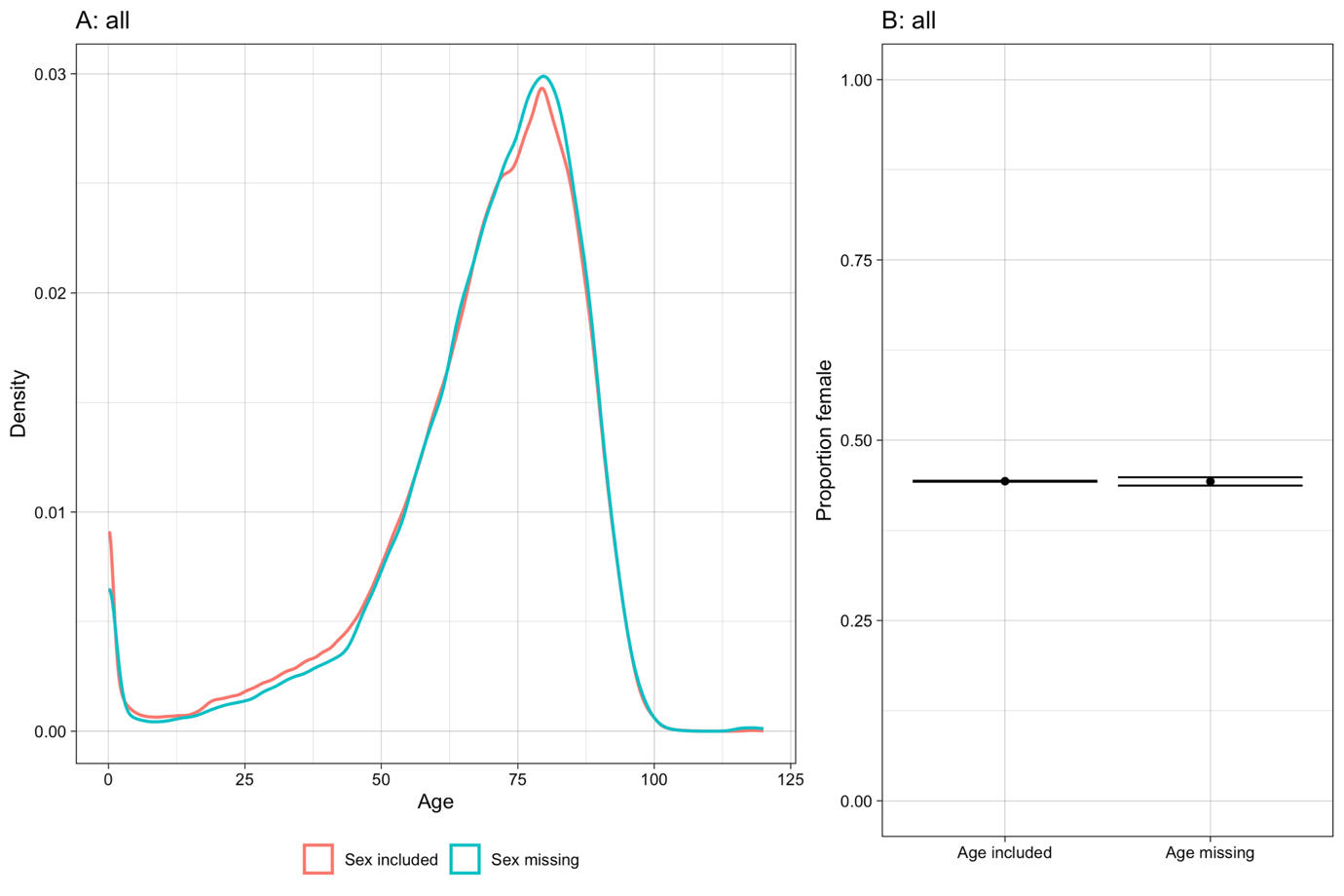


Figure 1: A) Density of individuals across age groups, for those individuals including and missing sex. B) Proportion and 95% credible interval of female indivduals, subsetted for those including and missing age.


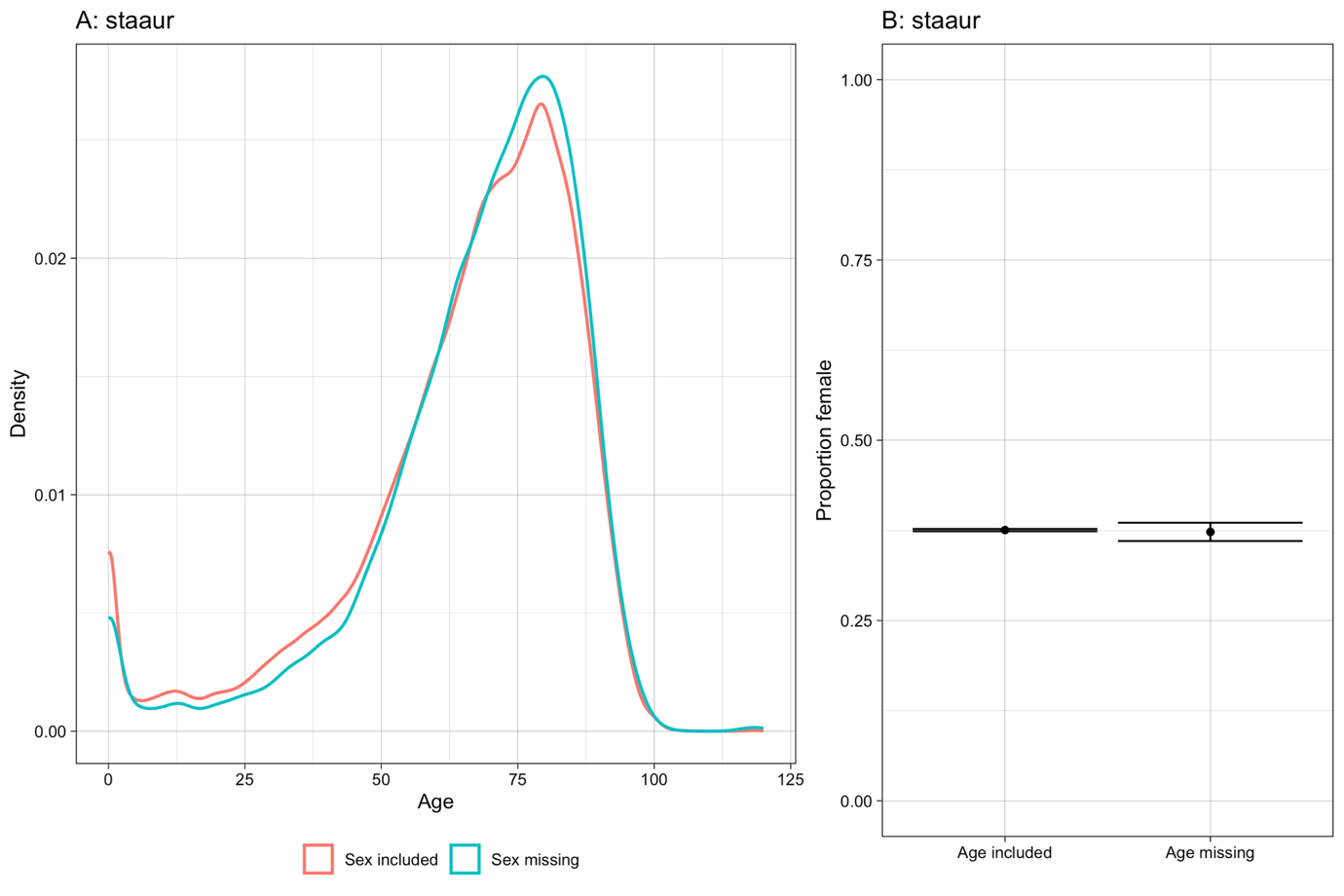


Figure 2: A) Density of individuals across age groups infected with S.aureus, for those individuals including and missing sex. B) Proportion and 95% credible interval of female indivduals infected with S.aureus, subsetted for those including and missing age.

Not all samples were tested for each resistance type, and hence were coded as NA values in the data. The proportion of samples that were not tested for susceptibility ranged between 1.4% and 51.8%, depending on the bacteria-antibiotic combination (Table A1).

We analysed the missingness of susceptibility test data for our case study MRSA, by age and sex. The distribution of ages between those missing and not missing susceptibility information followed the same trend and there were no differences in susceptibility missingness by sex (Figure 3).

*
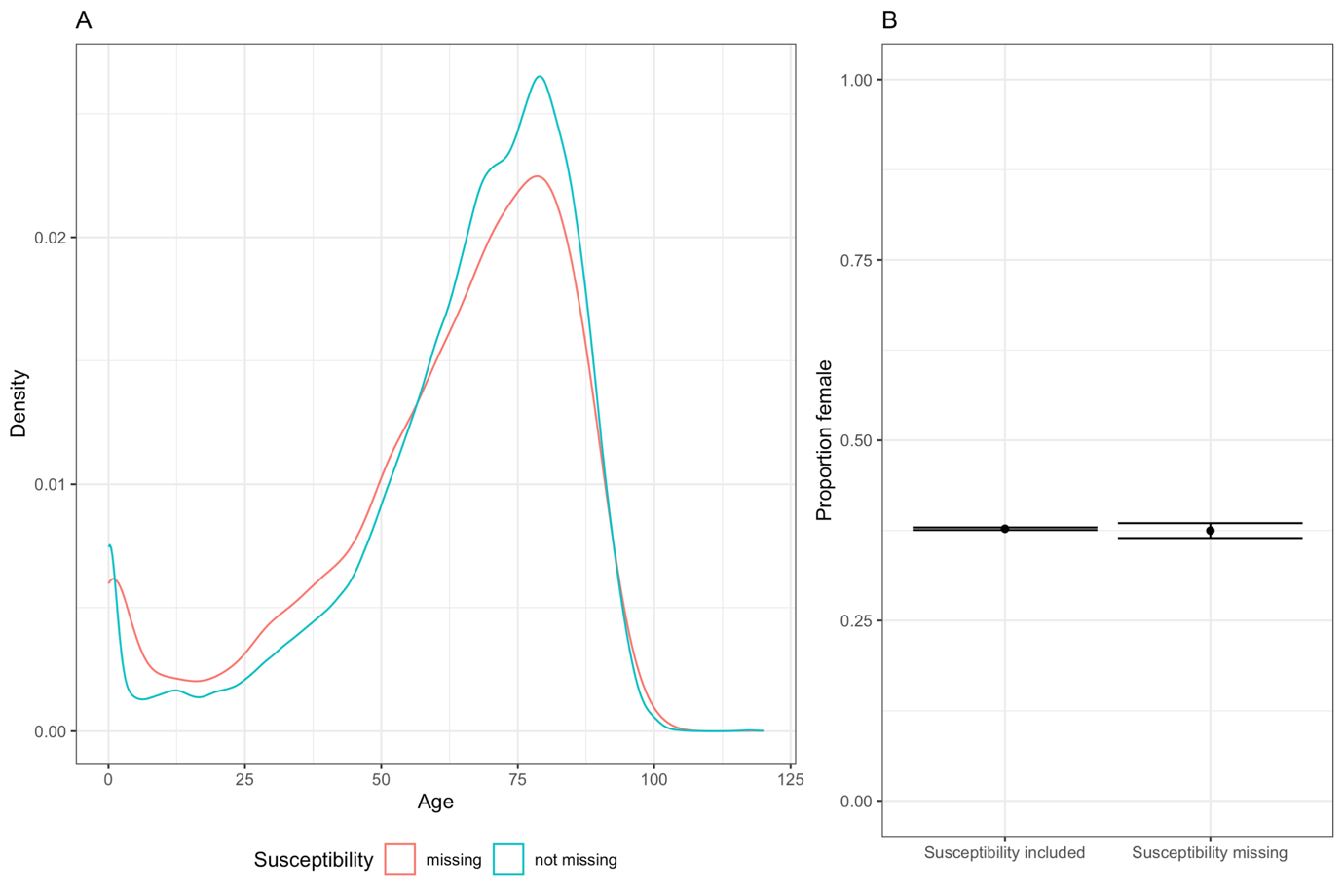
*

Figure 3: A) Density of individuals across age groups infected with S.aureus, for those individuals including and missing susceptibility tests. B) Proportion and 95% credible interval of female indivduals infected with S.aureus, subsetted for those including and missing susceptbility tests.

***Final cleaned dataset for resistance prevalence***

The final dataset for the analysis of resistance prevalence filtered for data with (a) age and sex values, (b) for the time period 2015-2019, with (c) multi-resistance class groupings removed and (d) only those aged 1 or older, consisted of data on 17 bug-drug combinations across 8 bacteria (Table 1 of main manuscript, Table A1) and 29 countries (Table A2). Our main analysis therefore used a dataset consisting of a total of 6,862,577 susceptibility results consisting of 74% of the original dataset (i.e. when taking the number of susceptibility tests in the cleaned data divided by the number of potential susceptibility tests (1s or 0s) with no filtering on age, sex or multi-resistance in 2015-2019).

Table A1: AWaRE classification of bacteria antibiotics in data set (> 2015) and percentage of bacterial samples tested for resistance by bacteria and antibiotic.

| **Bacteria** | **Code in data** | | **Antibiotic** | **AWaRE 2021 classification** | **Percentage of bacterial samples tested for antibiotic (%)** |
| --- | --- | --- | --- | --- | --- |
| Acinetobacter species | acispp | amika_R | Amikacin | Access | 71.8 |
|  | acispp | aminogl_R | Aminoglycosides | Access + Watch | 96.9 |
|  | acispp | carbapen_R | Carbapenems | Watch | 98.4 |
|  | acispp | fq_pseudo_R | Fluroquinolones | Watch | 97.5 |
| *Enterococcus faecalis* | encfae | aminopen_R | Aminopenicillins | Access | 93.7 |
|  | encfae | genta_high | High-level aminoglycoside | Access | 59.5 |
|  | encfae | vanco_R | Vancomycin | Watch | 97.8 |
| *Enterococcus faecium* | encfai | aminopen_R | Aminopenicillins | Access | 94.4 |
|  | encfai | genta_high | High-level aminoglycoside | Access | 57.5 |
|  | encfai | vanco_R | Vancomycin | Watch | 98.3 |
| *Escherichia coli* | esccol | amika_R | Amikacin | Access | 56.7 |
|  | esccol | aminogl_R | Aminoglycosides | Access + Watch | 97.8 |
|  | esccol | aminopen_R | Aminopenicillins | Access | 82.7 |
|  | esccol | carbapen_R | Carbapenems | Watch | 96.0 |
|  | esccol | cefIII_entero_R | Third-generation cephalosporins | Watch + Reserve | 97.3 |
|  | esccol | ert_R | Ertapenem | Watch | 45.4 |
|  | esccol | fq_ent_R | Fluoroquinolones | Watch | 98.3 |
|  | esccol | ureidopen_R | piperacillin-tazobactam | Watch | 71.6 |
| *Klebsiella pneumoniae* | klepne | amika_R | Amikacin | Access | 66.4 |
|  | klepne | aminogl_R | Aminoglycosides | Access + Watch | 97.6 |
|  | klepne | carbapen_R | Carbapenems | Watch | 96.9 |
|  | klepne | cefIII_entero_R | Third-generation cephalosporins | Watch + Reserve | 98.0 |
|  | klepne | ert_R | Ertapenem | Watch | 48.2 |
|  | klepne | fq_ent_R | Fluoroquinolone | Watch | 98.5 |
|  | klepne | ureidopen_R | piperacillin-tazobactam | Watch | 75.4 |
| *Pseudomonas aeruginosa* | pseaer | amika_R | Amikacin | Access | 79.4 |
|  | pseaer | aminogl_R | Aminoglycoside | Access + Watch | 91.4 |
|  | pseaer | carbapen_R | Carbapenem | Watch | 98.6 |
|  | pseaer | ceftaz_R | Ceftazidime | Watch | 96.6 |
|  | pseaer | fq_pseudo_R | Fluoroquinolone | Watch | 98.4 |
|  | pseaer | ureidopen_R | piperacillin-tazobactam | Watch | 95.3 |
| *Staphylococcus aureus* | staaur | fq_staaur_R | Fluoroquinolone | Watch | 87.3 |
|  | staaur | mrsa_R | MRSA (oxacillin or cefoxitin) | Access | 97.4 |
|  | staaur | rifamp_R | Rifampicin | Watch | 77.8 |
| *Streptococcus pneumoniae* | strpne | cefIII_strpne_R | Third-generation cephalosporins | Watch + Reserve | 71.1 |
|  | strpne | fq_strpne_R | Fluoroquinolone | Watch | 71.8 |
|  | strpne | macrol_R | Macrolide | Watch | 96.2 |
|  | strpne | penic_RI | Penicillins | Access + Watch | 93.3 |

Table A2: Number of susceptibility samples by bacteria-antibiotic (rows) and country (column) in cleaned dataset. Country labels are random anonymised three letter code used for this analysis only and consistent throughout.

| pathogen | name | WLI | YGW | UMV | PUB | VYQ | BKP | KXR | LKP | EJQ | IFY | AKX | FLV | PMS | CGZ | FGI | BPQ | MUV | ANW | NSL | IGC | SEG | NAJ | CIP | ZYI | KVX | XTL | ABO | ZVM | QET | TOTAL |
| --- | --- | --- | --- | --- | --- | --- | --- | --- | --- | --- | --- | --- | --- | --- | --- | --- | --- | --- | --- | --- | --- | --- | --- | --- | --- | --- | --- | --- | --- | --- | --- |
| acispp | **amika_R** | 516 | 280 | 520 | 836 | 57 | 357 | 125 | 0 | 406 | 17 | 1828 | 817 | 1584 | 1364 | 273 | 145 | 2 | 2943 | 42 | 247 | 47 | 116 | 50 | 14 | 1234 | 549 | 120 | 202 | 607 | **15298** |
| acispp | **aminogl_R** | 646 | 284 | 494 | 869 | 193 | 357 | 1840 | 321 | 434 | 171 | 2071 | 833 | 1634 | 2789 | 391 | 300 | 18 | 3777 | 44 | 246 | 45 | 597 | 35 | 148 | 1553 | 947 | 317 | 202 | 618 | **22174** |
| acispp | **carbapen_R** | 609 | 335 | 530 | 870 | 193 | 357 | 1956 | 314 | 433 | 175 | 2086 | 841 | 1647 | 2731 | 390 | 316 | 18 | 3737 | 37 | 253 | 47 | 596 | 54 | 149 | 1552 | 984 | 325 | 202 | 592 | **22329** |
| acispp | **fq_pseudo_R** | 605 | 339 | 533 | 865 | 192 | 357 | 1936 | 330 | 432 | 175 | 2088 | 828 | 1631 | 2750 | 387 | 324 | 18 | 3741 | 44 | 214 | 47 | 606 | 41 | 149 | 1536 | 980 | 321 | 202 | 613 | **22284** |
| encfae | **aminopen_R** | 457 | 1774 | 620 | 4327 | 249 | 2631 | 12949 | 3015 | 4730 | 2078 | 9149 | 777 | 3205 | 9181 | 3555 | 1450 | 136 | 10803 | 309 | 264 | 149 | 4625 | 314 | 2515 | 3140 | 657 | 4589 | 739 | 1130 | **89517** |
| encfae | **genta_high** | 455 | 1167 | 594 | 4089 | 249 | 2629 | 8957 | 387 | 4380 | 0 | 5603 | 771 | 3209 | 0 | 2085 | 1294 | 136 | 7456 | 297 | 369 | 146 | 3071 | 312 | 976 | 2947 | 483 | 3998 | 722 | 1049 | **57831** |
| encfae | **vanco_R** | 500 | 1773 | 613 | 4618 | 249 | 2630 | 13353 | 2840 | 4845 | 2604 | 9775 | 777 | 3184 | 9286 | 3678 | 1470 | 136 | 10964 | 310 | 381 | 149 | 4618 | 317 | 2454 | 3224 | 675 | 4690 | 739 | 1143 | **91995** |
| encfai | **aminopen_R** | 379 | 1148 | 335 | 1990 | 176 | 1543 | 9103 | 3572 | 3158 | 1137 | 4056 | 404 | 1254 | 8800 | 2536 | 2083 | 79 | 6170 | 153 | 154 | 59 | 4256 | 253 | 978 | 1639 | 403 | 2568 | 650 | 638 | **59674** |
| encfai | **genta_high** | 372 | 766 | 324 | 1907 | 177 | 1543 | 6468 | 399 | 2880 | 0 | 2651 | 401 | 1261 | 0 | 1438 | 1866 | 78 | 4311 | 145 | 227 | 59 | 3093 | 262 | 452 | 1619 | 314 | 2275 | 640 | 543 | **36471** |
| encfai | **vanco_R** | 397 | 1161 | 332 | 2149 | 176 | 1544 | 9561 | 3653 | 3235 | 1470 | 4310 | 405 | 1259 | 9037 | 2632 | 2127 | 79 | 6269 | 152 | 233 | 59 | 4259 | 277 | 966 | 1773 | 418 | 2597 | 650 | 675 | **61855** |
| esccol | **amika_R** | 1319 | 15066 | 1260 | 26650 | 245 | 4008 | 14111 | 0 | 32449 | 0 | 53632 | 4988 | 8539 | 55710 | 18388 | 12941 | 1 | 42897 | 2069 | 1221 | 1519 | 4668 | 2233 | 1036 | 12021 | 1060 | 19569 | 7341 | 4228 | **349169** |
| esccol | **aminogl_R** | 1321 | 13796 | 1192 | 28849 | 588 | 16301 | 77720 | 25320 | 34292 | 23422 | 53919 | 5205 | 8987 | 119115 | 26881 | 14900 | 1009 | 43786 | 2068 | 1361 | 1519 | 36880 | 3452 | 18373 | 11465 | 2483 | 32993 | 7343 | 4299 | **618839** |
| esccol | **aminopen_R** | 1301 | 15758 | 1117 | 28139 | 588 | 16288 | 78610 | 24923 | 33365 | 14064 | 54666 | 5234 | 8882 | 110126 | 26376 | 14738 | 1011 | 16809 | 2066 | 1365 | 1519 | 36871 | 2029 | 18370 | 3859 | 2166 | 395 | 7343 | 4249 | **532227** |
| esccol | **carbapen_R** | 1325 | 15852 | 1242 | 28745 | 587 | 7645 | 79072 | 23833 | 34235 | 24764 | 53202 | 5191 | 8665 | 115881 | 26336 | 14860 | 29 | 41942 | 2069 | 1364 | 1519 | 36862 | 3028 | 18338 | 11566 | 2555 | 32541 | 7343 | 4027 | **604618** |
| esccol | **cefIII_entero_R** | 1326 | 15869 | 1274 | 28942 | 588 | 16297 | 79110 | 23801 | 34268 | 24545 | 54555 | 5185 | 9095 | 107584 | 26794 | 14883 | 1011 | 43853 | 2069 | 1386 | 1519 | 36873 | 3446 | 18378 | 12186 | 2570 | 33291 | 7343 | 4290 | **612331** |
| esccol | **ert_R** | 764 | 7924 | 0 | 26887 | 120 | 0 | 47580 | 622 | 22040 | 2902 | 0 | 0 | 8195 | 62280 | 7525 | 10665 | 939 | 32612 | 1189 | 252 | 1191 | 2810 | 2332 | 2116 | 4672 | 780 | 7470 | 7323 | 3672 | **264862** |
| esccol | **fq_ent_R** | 1315 | 15130 | 1273 | 28948 | 588 | 16284 | 79044 | 25281 | 33618 | 24773 | 54668 | 5230 | 9064 | 116592 | 26955 | 14876 | 964 | 43422 | 2063 | 1367 | 1519 | 36876 | 3413 | 18361 | 11049 | 2556 | 32821 | 7291 | 4307 | **619648** |
| esccol | **ureidopen_R** | 1323 | 13921 | 522 | 25447 | 576 | 6398 | 77694 | 15463 | 31532 | 0 | 0 | 0 | 8579 | 73646 | 18247 | 0 | 938 | 42917 | 1757 | 281 | 1517 | 36003 | 1721 | 16133 | 11230 | 867 | 32589 | 7337 | 3906 | **430544** |
| klepne | **amika_R** | 1085 | 2774 | 754 | 11355 | 84 | 5610 | 2606 | 0 | 8309 | 0 | 12484 | 1457 | 2941 | 10021 | 3952 | 1964 | 0 | 16263 | 419 | 606 | 558 | 1025 | 513 | 232 | 5100 | 635 | 2636 | 1384 | 2157 | **96924** |
| klepne | **aminogl_R** | 1089 | 2474 | 729 | 12008 | 326 | 7054 | 14178 | 5898 | 8664 | 3656 | 12645 | 1501 | 3074 | 21195 | 5717 | 2277 | 115 | 16583 | 419 | 705 | 558 | 6717 | 779 | 3832 | 4981 | 1696 | 5970 | 1384 | 2186 | **148410** |
| klepne | **carbapen_R** | 1093 | 2889 | 768 | 11981 | 328 | 5654 | 14400 | 5599 | 8662 | 3824 | 12484 | 1493 | 2988 | 20694 | 5734 | 2276 | 3 | 16192 | 419 | 705 | 557 | 6711 | 688 | 3827 | 5050 | 1756 | 6313 | 1384 | 2079 | **146551** |
| klepne | **cefIII_entero_R** | 1095 | 2906 | 769 | 12043 | 328 | 7051 | 14401 | 5565 | 8670 | 3799 | 12724 | 1484 | 3074 | 19408 | 5844 | 2274 | 115 | 16658 | 419 | 713 | 558 | 6715 | 810 | 3836 | 5173 | 1780 | 6424 | 1384 | 2172 | **148192** |
| klepne | **ert_R** | 533 | 1358 | 0 | 11151 | 38 | 0 | 8775 | 110 | 5979 | 525 | 0 | 0 | 2786 | 11155 | 1590 | 1599 | 108 | 12828 | 198 | 146 | 431 | 588 | 518 | 436 | 1898 | 384 | 726 | 1378 | 1824 | **67062** |
| klepne | **fq_ent_R** | 1089 | 2876 | 770 | 12057 | 328 | 7049 | 14397 | 5894 | 8572 | 3834 | 12738 | 1497 | 3049 | 20785 | 5895 | 2275 | 109 | 16555 | 419 | 702 | 558 | 6715 | 780 | 3831 | 4702 | 1764 | 6321 | 1378 | 2183 | **149122** |
| klepne | **ureidopen_R** | 1089 | 2585 | 325 | 10946 | 321 | 5585 | 14209 | 3572 | 8239 | 0 | 0 | 0 | 2879 | 13034 | 4038 | 0 | 108 | 16291 | 370 | 160 | 558 | 6522 | 353 | 3381 | 4880 | 542 | 4788 | 1384 | 1933 | **108092** |
| pseaer | **amika_R** | 554 | 1462 | 361 | 5648 | 224 | 2068 | 5517 | 0 | 4245 | 0 | 8601 | 1060 | 3281 | 7562 | 3305 | 1111 | 1 | 8397 | 239 | 126 | 167 | 512 | 191 | 32 | 1833 | 591 | 1020 | 759 | 1101 | **59968** |
| pseaer | **aminogl_R** | 561 | 1301 | 339 | 5698 | 230 | 2461 | 6849 | 2351 | 4656 | 1915 | 8762 | 1070 | 3361 | 10856 | 3580 | 1260 | 78 | 8640 | 233 | 126 | 167 | 3487 | 241 | 1139 | 1758 | 621 | 2410 | 759 | 1106 | **76015** |
| pseaer | **carbapen_R** | 559 | 1528 | 364 | 5692 | 230 | 2462 | 6878 | 2279 | 4660 | 1918 | 8725 | 1071 | 3384 | 10679 | 3601 | 1259 | 78 | 8592 | 196 | 129 | 167 | 3481 | 241 | 1199 | 1791 | 644 | 2423 | 759 | 1066 | **76055** |
| pseaer | **ceftaz_R** | 557 | 1363 | 359 | 5675 | 230 | 2461 | 6859 | 2263 | 4560 | 1912 | 8486 | 1023 | 3368 | 10212 | 3314 | 1228 | 77 | 8448 | 239 | 128 | 167 | 3466 | 183 | 1154 | 1845 | 618 | 2406 | 759 | 982 | **74342** |
| pseaer | **fq_pseudo_R** | 561 | 1456 | 364 | 5679 | 230 | 2461 | 6879 | 2333 | 4657 | 1789 | 8728 | 1067 | 3376 | 10710 | 3593 | 1257 | 78 | 8581 | 239 | 129 | 167 | 3487 | 242 | 1203 | 1788 | 640 | 2400 | 744 | 1106 | **75944** |
| pseaer | ureidopen_R | 541 | 1398 | 358 | 5661 | 230 | 2400 | 6774 | 2354 | 4511 | 1904 | 8444 | 1049 | 3342 | 10243 | 3256 | 1255 | 2 | 8359 | 221 | 122 | 167 | 3296 | 241 | 1117 | 1717 | 596 | 2423 | 759 | 989 | **73729** |
| staaur | **fq_staaur_R** | 565 | 4987 | 1217 | 15151 | 510 | 7827 | 41667 | 2504 | 10311 | 10421 | 27109 | 2143 | 6931 | 34369 | 12424 | 5217 | 31 | 22425 | 788 | 1492 | 427 | 14788 | 1272 | 5483 | 7703 | 2589 | 12703 | 2760 | 2791 | **258605** |
| staaur | **mrsa_R** | 603 | 5340 | 1247 | 17238 | 504 | 9672 | 42892 | 9990 | 10397 | 10869 | 27692 | 2163 | 6987 | 35265 | 15415 | 5492 | 422 | 22362 | 885 | 1436 | 427 | 15078 | 1439 | 7427 | 8047 | 2443 | 19365 | 2840 | 2794 | **286731** |
| staaur | **rifamp_R** | 163 | 4061 | 682 | 10278 | 311 | 9667 | 36896 | 2504 | 8363 | 10700 | 27808 | 1912 | 3936 | 34728 | 14393 | 4974 | 32 | 17347 | 707 | 1372 | 423 | 15021 | 147 | 3635 | 3296 | 1826 | 12035 | 2840 | 2528 | **232585** |
| strpne | **cefIII_strpne_R** | 0 | 6836 | 121 | 4488 | 56 | 1557 | 6115 | 0 | 3825 | 0 | 4624 | 595 | 836 | 6516 | 2240 | 1818 | 141 | 3325 | 205 | 171 | 106 | 5411 | 307 | 1833 | 1174 | 324 | 2749 | 1396 | 139 | **56908** |
| strpne | **fq_strpne_R** | 0 | 6844 | 164 | 4352 | 53 | 1659 | 6419 | 1439 | 3669 | 0 | 4624 | 669 | 744 | 11147 | 2279 | 962 | 1 | 3278 | 187 | 277 | 106 | 3149 | 244 | 0 | 1161 | 383 | 3369 | 1432 | 51 | **58662** |
| strpne | **macrol_R** | 0 | 6844 | 175 | 4541 | 54 | 1656 | 6622 | 3500 | 3811 | 3636 | 4624 | 675 | 796 | 16588 | 2483 | 1766 | 145 | 3222 | 206 | 275 | 103 | 7860 | 572 | 2212 | 1337 | 332 | 4210 | 1333 | 153 | **79731** |
| strpne | **penic_RI** | 0 | 6844 | 178 | 4410 | 42 | 1659 | 6568 | 3500 | 3832 | 3245 | 4624 | 679 | 854 | 15631 | 2301 | 1836 | 145 | 2590 | 200 | 314 | 106 | 7535 | 625 | 2384 | 1476 | 345 | 3768 | 1432 | 160 | **77283** |

## Incidence calculations

The calculation of incidence used all data that had (a) age and (b) sex (male / female) recorded in those aged 1 or older. Excluding those isolates without age or sex information removed 4.8% or 7.1% of isolates respectively. Due to the overlap in missing data, the final data set for incidence included 91% of the original isolates (a total of 3,231,153 isolates). For 2019, the year that we report incidence in the main analysis, the number of isolates used across Europe varied by the bacterial species (Table A3).

| Bacterial species | Number of isolates in 2019 |
| --- | --- |
| *Acinetobacter spp* | 5,637 |
| *Enterococcus faecalis* | 23,717 |
| *Enterococcus faecium* | 15,778 |
| *Escherichia coli* | 154,071 |
| *Klebsiella pneumoniae* | 39,708 |
| *Pseudomonas aeruginosa* | 19,497 |
| *Staphylococcus aureus* | 72,016 |
| *Streptococcus pneumoniae* | 19,024 |

Table A3: Number of isolates in the dataset use to calculate incidence across Europe in 2019.

The raw data can be used to calculate the number of *isolates* taken from patients of each age which can be standardised by the population in each age group. However, the network of contributing laboratories is not a population-based surveillance network – the number of isolates does not always reflect the total number of infections in a country. Therefore, in order to estimate the incidence of bloodstream infections by age we need to inflate the reported numbers by some indication of what proportion of the infections are captured i.e. the coverage.

*Coverage complexity*

The hurdles to understanding coverage are two-fold. Firstly, not all microbiology laboratories responsible for processing isolates are linked to the EARSS/EARS-Net network. In the ECDC AMR report of 2017 [3] (and previous years), the number of laboratories reporting at least one isolate to the EARSS/EARS-Net surveillance dataset is shown for the 2000-2016 period. The number of laboratories reporting is stable or increasing over this period, showing how coverage has improved.

However, the size and number of laboratories varies by country and hence it is hard to get an estimate for coverage or representativeness from this. Moreover, the overlapping hospital population catchment areas and movement of patients for care seeking means that the exact proportion of the population included in the data is highly difficult to assess. It requires local knowledge and will likely always be an approximation.

Hence, ECDC AMR reports since 2018 have asked countries to report an “Estimated population country coverage”. This is a self-assessed national coverage and sample representativeness value as estimated by the National Focal Points for AMR and/or Operational Contact Points for AMR (Table 2.1 in the 2018 report, Table 1 in the 2019 report, Table 2 in the 2020 report ). This is reported across all bacteria in the EARS-Net reports and shows a large variation between countries and some variation in time (Figure 4).

Figure 4: Combined estimated population coverage of the isolates reported in EARS-Net from the annual antimicrobial resistance surveillance reports [4] and estimates reported to Cassini et al. (shown with crosses).

It is likely that there is further variation in coverage or representativeness when comparing between bacteria. This is shown in data collected by Cassini and colleagues for their estimates of deaths and disability-adjusted life-years caused by antibiotic resistance bacteria in 2015 [5]. Designated contact points in each Member State were given the possibility of providing an estimated “percentages of population covered” to give national population coverage (Table 3 in the Supplementary material of [5]). This showed that despite wide variation between countries (Figure 5), for 70% (21/30) of countries that reported values, the estimated coverage was the same across bacteria in 2015. Two countries had more than 50% variation between coverage –high coverage of *S. pneumoniae* (67%/87%) being an outlier against 18% coverage of other bacteria or 24% for others apart from *Acinobacter spp.* (8%) for France and Belgium respectively.

Figure 5: Estimated population coverage of EARS-Net from country experts as reported to the Cassini study [5].

The second hurdle is the variation in local sampling procedures – not just what patients might have their isolates sent to the laboratory but how many patients with an infection will even be sampled and where within the hospital and when. EARS-Net has attempted to address this by considering the blood culture rate (blood culture sets / 1,000 patient-days). This is complicated further by the varying country definitions of a blood culture set as well as a “patient day”. Thus, it is hard to use this indicator – in the ECDC reports it is seen as another factor to bear in mind when interpreting resistance trends and Cassini et al do not use it, after consideration, in their methods.

The added complexity of any variation in sampling by age or patient sex has no information to support any assumptions.

Only the values for Italy (Figure 4) gave a significant trend under a linear model testing for increasing coverage over time, and so it was decided to

1. use the country level values to inflate number of isolates where they exist for estimates of incidence
2. to use the last value available for a country as the value to inflate the data for total number of isolates in projections. Both the number of isolates that are likely to be reported to EARS-Net and the inflated totals per country are reported.

Our sensitivity analysis explored using the minimum coverage value instead of the last.

Cerebrospinal fluid isolates represent a small minority of the EARS-NET data (< 4% of all isolates, only rising to 6% for *S. pneumoniae*). Patients from whom these isolates were taken are likely to also have a bloodstream infection (BSI), so, following Cassini et al, we include them in our estimates of BSI.

## Population trends – 2000 to 2021

The number of individuals in each age and sex group for the period of 2000-2021 was needed for the calculation of age-standardised incidence rates. Population sizes for 5yr age groupings were available from the World Bank DataBank [6] up to the age of 80 and then all adults were pooled. These are World Bank staff estimates using the World Bank's total population and age/sex distributions of the United Nations Population Division's World Population Prospects: 2019 Revision [7]. The trends vary substantially by country with an increase in the proportion in older ages present in most countries.

## Model fitting

*Variables included*

To determine whether country and laboratory level variation should be included in the model, we ran a variance-components model (using the R *glmer* functions) for MRSA, using this as a case study for the other bacteria-antibiotic combinations. Results showed that 33% of susceptibility variation lies between countries, 38% of susceptibility variations lies between laboratory ID and country combined. Figure 7 shows the variation across different laboratories and countries.


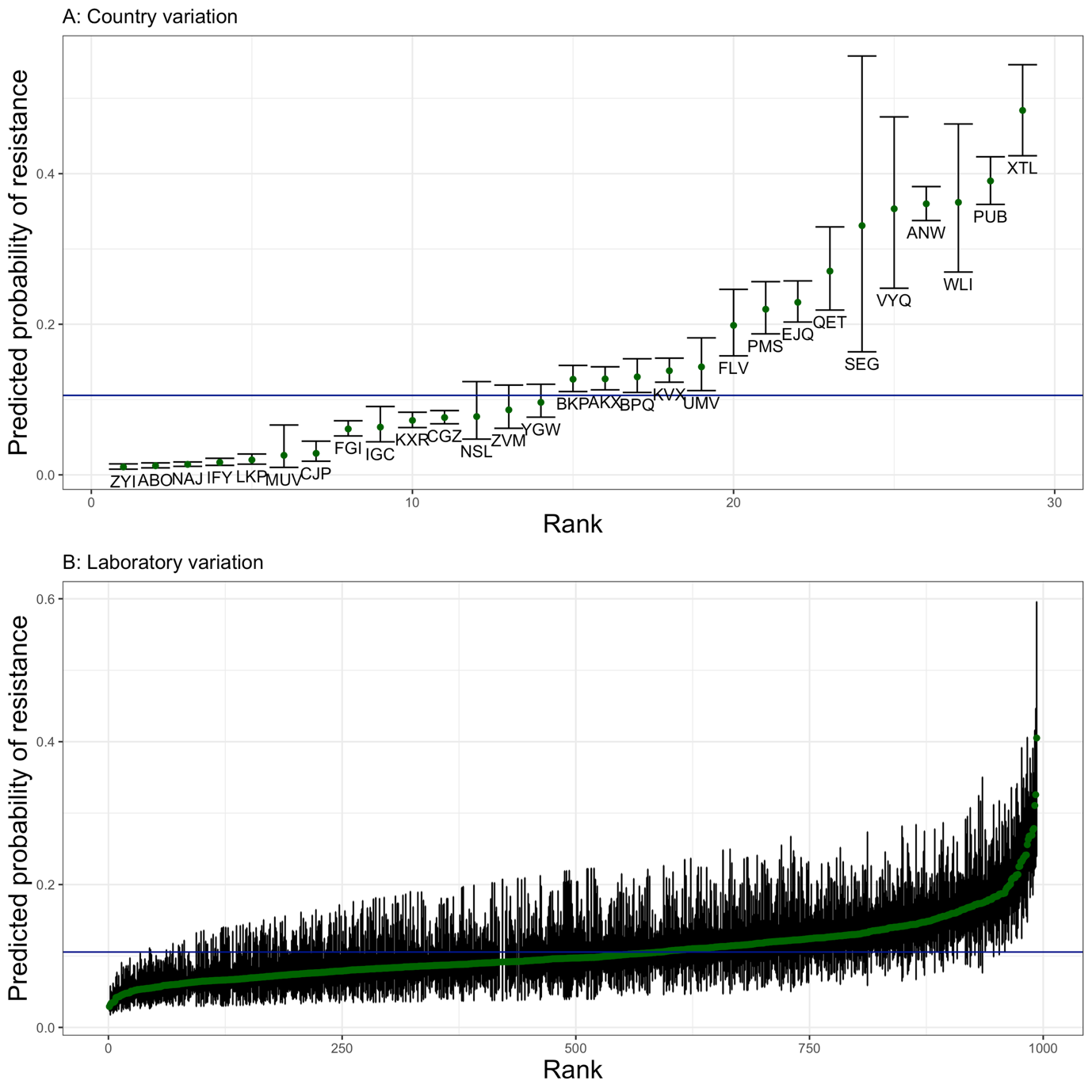


Figure 7: Predicted probability of resistance across (A) hospitals within a country and (B) laboratory ids over all countries.

*Variable transformations*

To achieve efficient model convergence, we transformed the input variables in our model to be on a similar scale. The transformations were as follows:

Model age <- Age / 100

Model age^2^ <- Model age * Model age

Years were converted onto a scale of 0-1 corresponding to the years 2015-2019.

*Fitting algorithm*

We used the brms package in R to run our models using stan software [8]. The algorithm used was no U-turn sampling (NUTS) which we ran for 3000 iterations for each model. We deemed models to not have converged if more than 1 divergent transition occurred. Bacteria-antibiotic combinations that did not converge were: *Escherichia coli* – Ertapenem*, Enterococcus faecalis* – aminopenicillins and *Enterococcus faecium* – vancomycin and *Streptococcus pneumoniae –* third-generation cephalosporins. We therefore excluded these combinations from our analysis, as they did not converge and they were not considered key bacteria-antibiotic combinations.

**References**

1. European Centre for Disease Prevention and Control. Antimicrobial resistance in the EU/EEA (EARS-Net) - Annual Epidemiological Report for 2019. Available from: https://www.ecdc.europa.eu/en/publications-data/surveillance-antimicrobial-resistance-europe-2019

2. European Centre for Disease Prevention and Control. EARS-Net Reporting protocol 2022. Available from: https://www.ecdc.europa.eu/en/publications-data/ears-net-reporting-protocol-2022

3. European Centre for Disease Prevention and Control. Antimicrobial resistance surveillance in Europe 2016. Available from: https://www.ecdc.europa.eu/en/publications-data/antimicrobial-resistance-surveillance-europe-2016

4. European Centre for Disease Prevention and Control. Annual Epidemiological Reports (AERs). 2022 Available from: https://www.ecdc.europa.eu/en/publications-data/monitoring/all-annual-epidemiological-reports

5. Cassini A, Högberg LD, Plachouras D, Quattrocchi A, Hoxha A, Simonsen GS, et al. Attributable deaths and disability-adjusted life-years caused by infections with antibiotic-resistant bacteria in the EU and the European Economic Area in 2015: a population-level modelling analysis. Lancet Infect Dis. 2019;19: 56–66. doi:10.1016/S1473-3099(18)30605-4

6. World Bank Group. DataBank; 2022 [cited 19 Oct 2023]. Available from: https://databank.worldbank.org/home

7. United Nations. World Population Prospects - Population Division. 2019 [cited 12 Mar 2021]. Available from: https://population.un.org/wpp/

8. Bürkner P-C. brms: An *R* Package for Bayesian Multilevel Models Using Stan. J Stat Softw. 2017;80: 1–28. doi:10.18637/jss.v080.i01

9. Central Asian and European surveillance of Antimicrobial Resistance (CAESAR), European Antimicrobial Resistance Surveillance Network (EARS-Net). Antimicrobial resistance surveillance in Europe - 2023. 2023 Apr. Available from: https://www.ecdc.europa.eu/en/publications-data/antimicrobial-resistance-surveillance-europe-2023
