# Supplementary material for: Antimicrobial resistance prevalence in bloodstream infection in 29 European countries by age and sex: An observational study
Source: PLoS Med. 2024 Mar 14;21(3):e1004301. doi: 10.1371/journal.pmed.1004301 (PMC10939247; doi:10.1371/journal.pmed.1004301)
Supplement: S2 Appendix — (DOCX) [file pmed.1004301.s002.docx]

**S2 Appendix: Additional results and sensitivity analysis for “Antimicrobial resistance prevalence in bloodstream infection in 29 European countries by age and sex: an observational study”**

Table of Contents

[1. Additional results: subregion analysis 2](#_Toc158789202)

[2. Additional results: Incidence 4](#_Toc158789203)

[3. Additional results: total number of resistant infections by age 6](#_Toc158789204)

[4. Additional results: Posterior estimates from model fit 7](#_Toc158789205)

[5. Additional results: Modelling 11](#_Toc158789206)

[6. Robustness of Index comparing resistance prevalence at ages 1 and 100 14](#_Toc158789207)

[7. Sensitivity analysis: data disaggregation by inpatient vs outpatient 18](#_Toc158789208)

[8. Sensitivity analysis: data disaggregation by hospital unit type 20](#_Toc158789209)

[9. Sensitivity analysis: incidence 23](#_Toc158789210)

[10. Sensitivity Analysis: Priors 24](#_Toc158789211)

[11. Sensitivity Analysis: Aged 0 25](#_Toc158789212)

[12. Sensitivity Analysis: Model structure 26](#_Toc158789213)

## 1. Additional results: subregion analysis

We observed substantial variation by subregion, matching previously reported large variation in resistance prevalence (with a north-to-south and west-to-east gradient of resistance) [1]) (Figure 1). Southern and Eastern Europe generally had higher resistance prevalence with substantial age / gender differences in Southern and Eastern Europe especially for *Acinetobacter spp.* and MRSA patterns driven by Southern and Eastern Europe, whilst for others the differences are less important e.g. for aminopenicillin resistance in *E. coli* (Figure 2). The patterns are still similar within a bacteria-antibiotic: i.e. the same across subregions within a bacteria-antibiotic and then different between bacteria-antibiotic combinations.


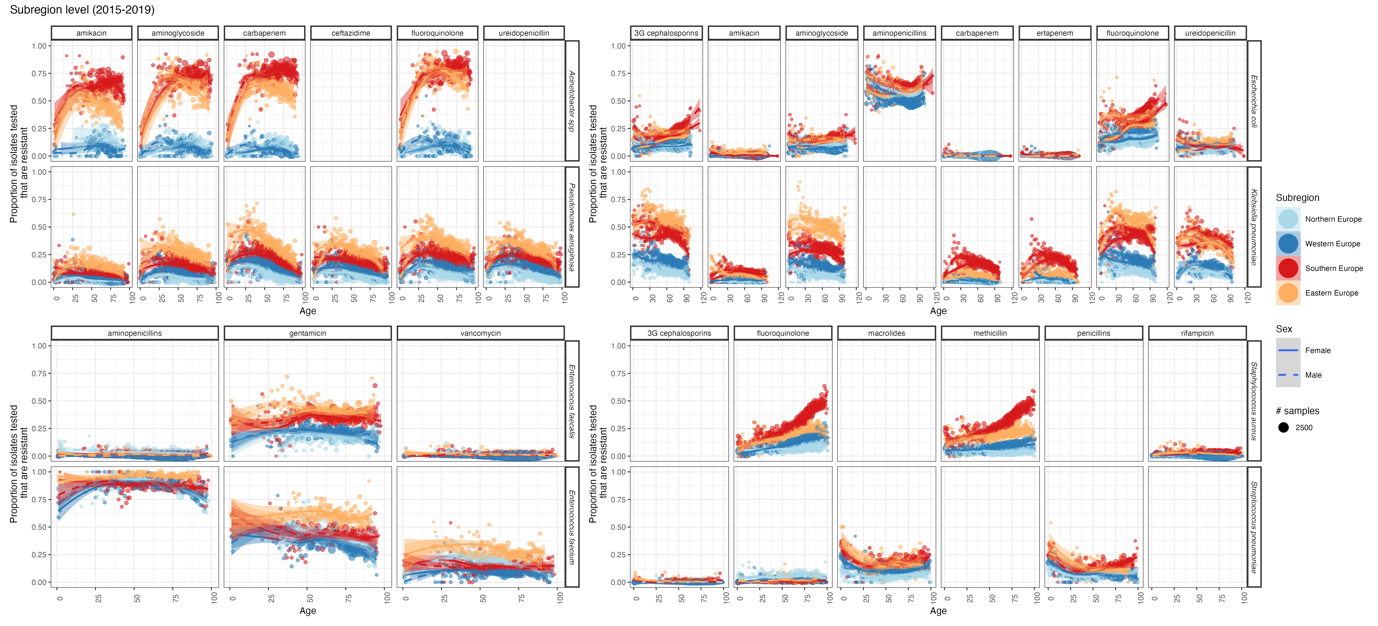


Figure 1: Trends in resistance prevalence vary by antibiotic, bacteria and demographic factors across the subregions of Europe. The proportion of isolates tested (y axis) that are resistant to each antibiotic (facet) within drug families (columns) for each bacteria (row) is shown for all European data grouped by subregion (colour) over 2015-2019 by age (x axis). Data is shown as points with number of samples indicated by size of point. Shaded areas are 95% confidence intervals around the LOESS fit line by sex(linetype).


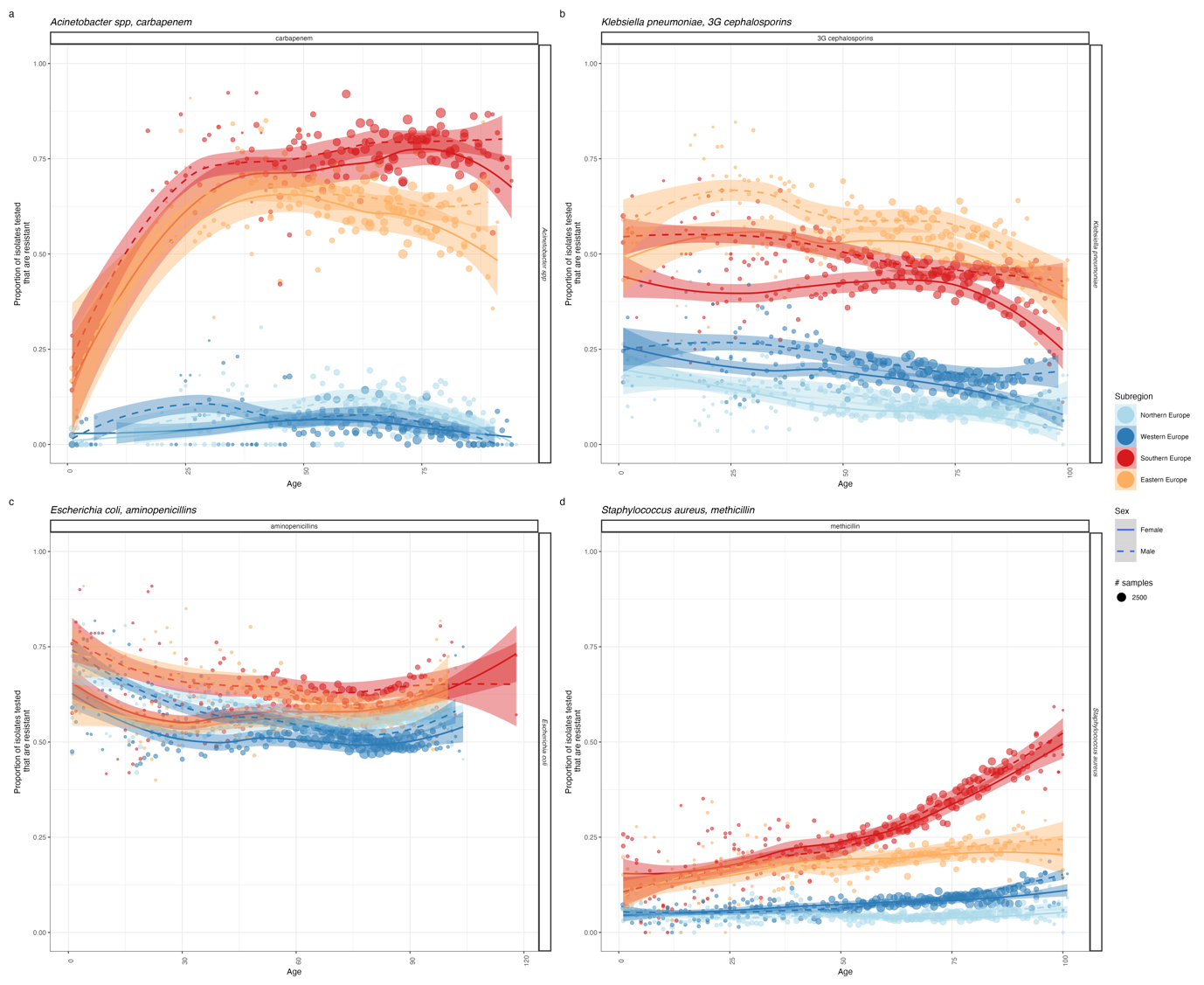


Figure 2: Zoomed in examples of how trends in resistance prevalence vary by antibiotic, bacteria and demographic factors across the subregions of Europe. The proportion of isolates tested (y axis) that are resistant to each antibiotic (facet) within drug families (columns and colour) for each bacteria (row) is shown for all European data grouped by subregion (colour) over 2015-2019 by age (x axis). Data is shown as points with number of samples indicated by size of point. Shaded areas are 95% confidence intervals around the LOESS fit line by sex (linetype).

## 2. Additional results: Incidence

The incidence of infection was calculated for all 29 countries in both datasets (population size and infection incidence). For this work we only included countries if they had reported data on the estimated coverage of these laboratories for any of the years this data was available: 2015, 2018-2020.

*Country level data and analysis*

These 28 countries were: "Austria", "Belgium", "Bulgaria", "Croatia", "Cyprus", "Czechia", "Denmark", "Estonia", "Finland", "France", "Germany", "Greece", "Hungary", "Iceland", "Ireland", "Italy", "Latvia", "Luxembourg", "Malta", "Netherlands", "Norway", "Poland", "Portugal", "Romania", "Slovak Republic", "Slovenia", "Spain", "Sweden" and “United Kingdom”. Not all countries were included in each data point. There was substantial variation between countries.

What is similar between countries is that there appears to be a trend to increasing incidence rates over time for those with data from 2015-2020. This reflects trends seen in earlier reports for *S. aureus* across Europe from the same data for 2005-2018 [2] and earlier trends for all bloodstream infections (BSIs) across Europe for 2002-2008 [3]. In individual countries increases have been seen in BSIs e.g. due to *E. faecalis* in Switzerland 2013-2018 [4,5], in England and Wales 1990-1998 [6] or England 2004-2008 [7]. We report this trend here but do not use it in our analyses.

Figure 3: Incidence of bloodstream infections per 100,000 population per year across European countries for 8 bacterial pathogens (panel) for multiple years (colour) and split by sex (solid line = female, dashed line = male). Shaded areas are 95% confidence intervals using a LOESS fit. Incidence plotted on a log scale (base 10).

## 3. Additional results: total number of resistant infections by age

The combination of exponential increase in infection by age with the proportion resistant, leads to an exponential increase in the number of infections with resistant bacteria by age (Figure 4).

**
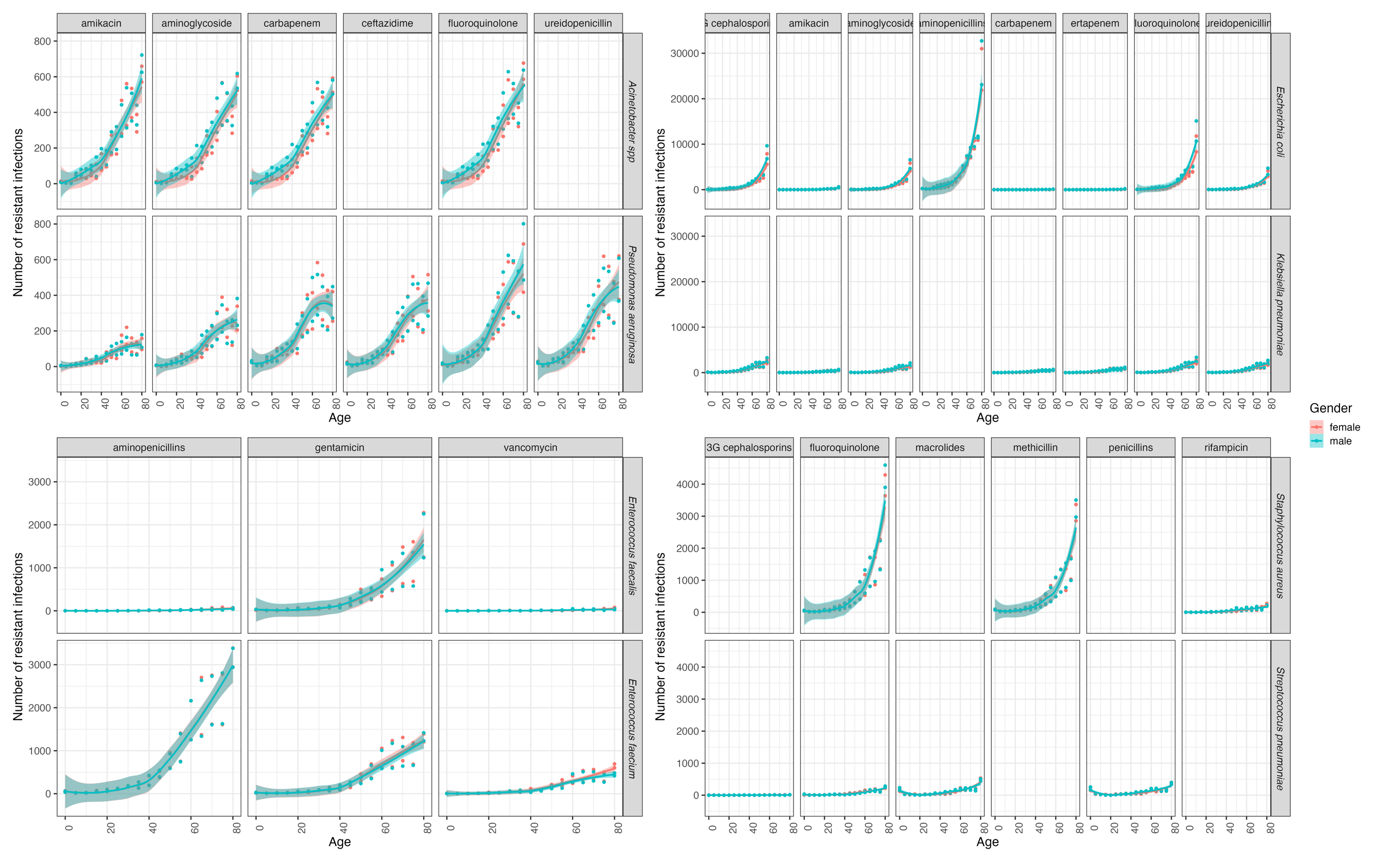
**

Figure 4: Predicted total number of bloodstream infections by age in 2019 across Europe with bacteria (row) resistant to different antibiotics (panels) by sex (colour). Shaded areas from a LOESS smoothing fit.

## 4. Additional results: Posterior estimates from model fit

Table A2 shows all the fixed effect parameter values and their 95% posterior credible intervals.

­­­Table A2: Posterior estimates for fixed effect parameter values. Blue indicates the 95% credible interval does not cross the null line. Shading matches Table 2 of main paper: orange shading indicates a positive coefficient and blue indicates a negative coefficient.

| **Bacteria** | **Antibiotic** | **year (**β_t_) | **Age (**β_a_)_‑_ | **age^2 (**β_a2_**)** | **sex(m) (**β_g_**)** | **Age:sex(m) (**β_ag_**)** |
| --- | --- | --- | --- | --- | --- | --- |
| *Acinetobacter species* | Amikacin | -0.03 (-0.17, 0.12) | 4.58 ( 3.36, 5.81) | -4.06 (-5.02, -3.11) | 0.22 (-0.08, 0.53) | -0.14 (-0.61, 0.33) |
|  | Aminoglycosides | -0.16 (-0.29, -0.03) | 5.24 ( 4.25, 6.23) | -4.33 (-5.13, -3.53) | 0.35 ( 0.08, 0.61) | -0.22 (-0.62, 0.19) |
|  | Carbapenems | -0.12 (-0.26, 0.01) | 5.44 ( 4.30, 6.57) | -4.85 (-5.74, -3.95) | 0.27 (-0.02, 0.56) | -0.02 (-0.46, 0.42) |
|  | Fluroquinolones | -0.19 (-0.32, -0.05) | 5.87 ( 4.83, 6.92) | -4.64 (-5.48, -3.80) | 0.42 ( 0.14, 0.69) | -0.31 (-0.73, 0.11) |
| *Enterococcus faecalis* | High-level aminoglycoside | -0.40 (-0.46, -0.34) | 1.42 ( 0.80, 2.03) | -1.14 (-1.61, -0.67) | 0.11 (-0.07, 0.29) | -0.05 (-0.30, 0.20) |
|  | Vancomycin | -0.41 (-0.67, -0.16) | 4.24 ( 1.70, 6.79) | -3.58 (-5.59, -1.57) | 0.66 (-0.02, 1.33) | -0.96 (-1.93, 0.00) |
| *Enterococcus faecium* | Aminopenicillins | 0.10 ( 0.02, 0.18) | 4.29 ( 3.48, 5.09) | -4.48 (-5.06, -3.89) | -0.07 (-0.30, 0.16) | 0.12 (-0.20, 0.44) |
|  | High-level aminoglycoside | -0.33 (-0.40, -0.25) | 2.12 ( 1.39, 2.85) | -1.97 (-2.56, -1.38) | -0.04 (-0.24, 0.17) | 0.07 (-0.22, 0.36) |
| *Escherichia coli* | Amikacin | 0.04 (-0.08, 0.17) | -0.95 (-2.04, 0.14) | 1.10 ( 0.25, 1.95) | 0.45 ( 0.12, 0.78) | -0.21 (-0.67, 0.24) |
|  | Aminoglycosides | -0.05 (-0.07, -0.02) | 0.35 (-0.02, 0.72) | 0.13 (-0.08, 0.35) | 0.49 ( 0.41, 0.57) | -0.34 (-0.45, -0.23) |
|  | Aminopenicillins | -0.08 (-0.10, -0.07) | -0.36 (-0.58, -0.14) | 0.35 ( 0.22, 0.49) | 0.42 ( 0.37, 0.48) | -0.41 (-0.48, -0.34) |
|  | Carbapenems | 1.74 ( 1.42, 2.05) | -3.23 (-5.37, -1.09) | 1.26 (-0.50, 3.02) | 0.78 ( 0.16, 1.40) | -0.47 (-1.34, 0.41) |
|  | Fluoroquinolones | -0.04 (-0.06, -0.02) | 1.35 ( 1.02, 1.68) | -0.40 (-0.57, -0.23) | 0.72 ( 0.66, 0.78) | -0.42 (-0.50, -0.33) |
|  | Third-generation cephalosporins | 0.05 ( 0.03, 0.08) | 0.50 ( 0.10, 0.90) | 0.16 (-0.05, 0.37) | 0.56 ( 0.49, 0.64) | -0.35 (-0.45, -0.25) |
|  | Piperacillin-tazobactam | -0.14 (-0.18, -0.10) | -0.01 (-0.45, 0.42) | 0.31 ( 0.00, 0.63) | 0.55 ( 0.44, 0.67) | -0.48 (-0.63, -0.33) |
| *Klebsiella pneumoniae* | Amikacin | -0.13 (-0.25, -0.02) | 1.52 ( 0.49, 2.55) | -1.88 (-2.65, -1.11) | 0.20 (-0.05, 0.45) | -0.08 (-0.45, 0.30) |
|  | Aminoglycosides | -0.13 (-0.18, -0.09) | -0.07 (-0.57, 0.43) | -0.50 (-0.83, -0.17) | 0.12 ( 0.00, 0.24) | 0.23 ( 0.06, 0.40) |
|  | Carbapenems | 0.49 ( 0.39, 0.58) | 2.33 ( 1.21, 3.45) | -3.31 (-4.00, -2.61) | 0.37 ( 0.15, 0.60) | -0.30 (-0.63, 0.02) |
|  | Ertapenem | 0.47 ( 0.36, 0.58) | 2.69 ( 1.69, 3.69) | -3.13 (-3.86, -2.40) | 0.27 ( 0.02, 0.52) | -0.17 (-0.53, 0.20) |
|  | Fluoroquinolones | 0.14 ( 0.09, 0.18) | 0.84 ( 0.38, 1.30) | -0.96 (-1.26, -0.66) | 0.14 ( 0.03, 0.25) | 0.22 ( 0.06, 0.37) |
|  | Third-generation cephalosporins | 0.04 ( 0.00, 0.08) | -0.13 (-0.59, 0.32) | -0.43 (-0.73, -0.13) | 0.15 ( 0.05, 0.26) | 0.20 ( 0.04, 0.35) |
|  | Piperacillin-tazobactam | 0.12 ( 0.07, 0.17) | 0.84 ( 0.36, 1.32) | -1.22 (-1.60, -0.85) | 0.09 (-0.04, 0.22) | 0.20 ( 0.01, 0.38) |
| *Pseudomonas aeruginosa* | Amikacin | -0.25 (-0.36, -0.13) | 1.74 ( 0.79, 2.70) | -2.69 (-3.49, -1.88) | 0.07 (-0.20, 0.35) | 0.07 (-0.35, 0.50) |
|  | Aminoglycosides | -0.34 (-0.42, -0.26) | 1.79 ( 1.14, 2.44) | -2.39 (-2.94, -1.84) | 0.11 (-0.08, 0.30) | 0.06 (-0.23, 0.35) |
|  | Carbapenems | -0.12 (-0.18, -0.05) | 2.10 ( 1.53, 2.66) | -3.52 (-3.98, -3.05) | 0.01 (-0.15, 0.16) | 0.10 (-0.14, 0.34) |
|  | Ceftazidime | -0.04 (-0.11, 0.03) | 1.25 ( 0.63, 1.86) | -2.21 (-2.70, -1.72) | 0.05 (-0.11, 0.22) | 0.06 (-0.19, 0.31) |
|  | Fluoroquinolone | -0.01 (-0.07, 0.05) | 2.22 ( 1.65, 2.78) | -2.59 (-3.04, -2.14) | -0.02 (-0.18, 0.14) | 0.25 ( 0.01, 0.48) |
|  | Piperacillin-tazobactam | -0.08 (-0.14, -0.01) | 1.21 ( 0.63, 1.79) | -2.17 (-2.63, -1.71) | 0.05 (-0.11, 0.21) | 0.04 (-0.20, 0.28) |
| *Staphylococcus aureus* | Fluoroquinolone | -0.38 (-0.41, -0.34) | 0.99 ( 0.46, 1.52) | 0.35 ( 0.08, 0.63) | -0.26 (-0.36, -0.16) | 0.50 ( 0.37, 0.64) |
|  | MRSA (oxacillin or cefoxitin) | -0.34 (-0.38, -0.30) | -0.75 (-1.35, -0.14) | 1.04 ( 0.78, 1.31) | -0.30 (-0.40, -0.21) | 0.55 ( 0.41, 0.68) |
|  | Rifampicin | -0.21 (-0.35, -0.06) | 4.06 ( 2.78, 5.34) | -3.81 (-4.81, -2.81) | 0.47 ( 0.13, 0.81) | -0.52 (-1.01, -0.03) |
| *Streptococcus pneumoniae* | Macrolide | -0.10 (-0.18, -0.03) | -2.77 (-3.21, -2.33) | 2.85 ( 2.50, 3.21) | 0.06 (-0.07, 0.19) | -0.14 (-0.33, 0.06) |
|  | Penicillins | 0.10 ( 0.02, 0.18) | -3.34 (-3.82, -2.87) | 2.93 ( 2.54, 3.31) | -0.04 (-0.18, 0.10) | - 1. -0.13, 0.28) |
|  | Fluoroquinolone | 2.20 ( 1.99 - 2.41) | 0.44 (-1.02 - 1.89) | 0.29 (-0.85 - 1.44) | 0.02 (-0.45 - 0.49) | 0.15 (-0.53 - 0.82) |


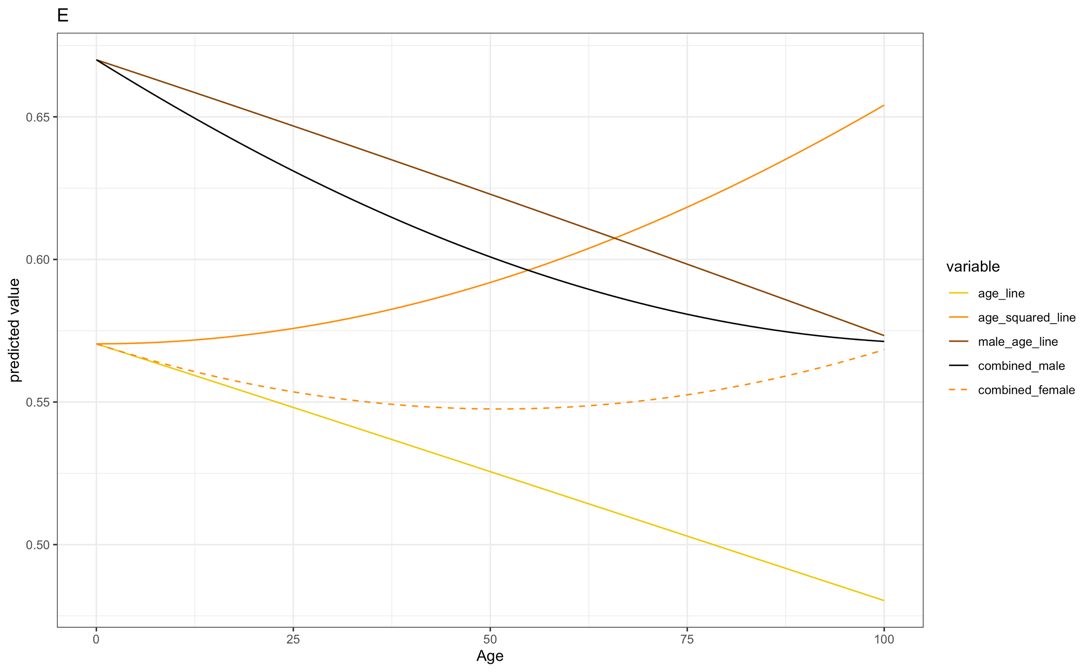

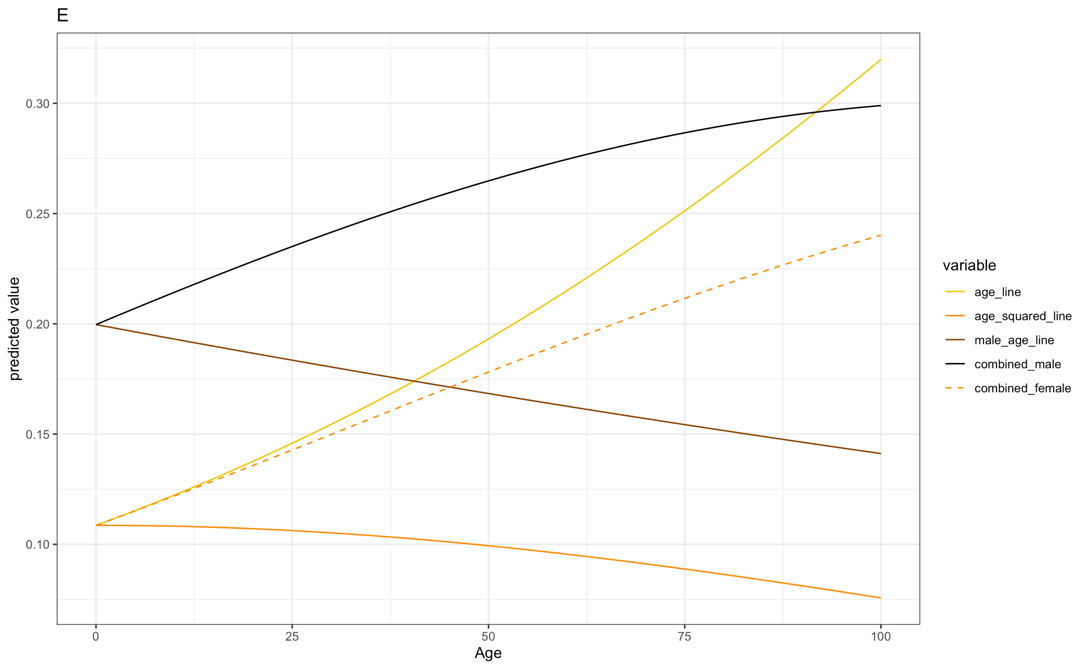


Figure 5: Decomposition plot for E. coli aminopenicillin (left) and fluoroquinolone (right) resistance showing components of model predicted output of resistance prevalence (y axis) by age (x axis) for each gender. Using the notation in Table A2, and in the methods, the predicted values is the inverse logit of the following equations: age_line = intercept + β_a_age, age_squared_line = intercept + β_a2_ age^2^, male_age_line = intercept + β_g_ +β_ag_ age, combined_male = intercept + β_g_ +β_ag_ age + β_a2_ age^2^+β_a_ age, combined_female = intercept + β_a2_ age^2^+β_a_ age. Note here that sex was coded as 1 for males, 0 for females.

Visualising the changes in coefficients in Table A2 (and Table 2 main analysis) can be done using the examples in Figure 5 for *E. coli* resistance to aminopenicillins and fluoroquinolones where the coefficients for age and age^2^ have opposite signs. This decomposition plot shows how the negative coefficient for age (yellow line) for aminopenicillin resistance in *E. coli* is counteracted by the positive coefficient for age^2^ (orange line) to give a combined “U” shaped curve for females (orange dashed). Sex affects the intercept, which for both of these resistances is higher for men, and there is an interaction term, which was negative here for both examples for men. For fluroquinolone resistance in *E. coli* the reversed sign for age and age^2^ coefficients is shown with the increasing linear component with age (yellow) but declining component for age^2^ (orange).

## 5. Additional results: Modelling

Our results also allow us to analyse the variation in age effect across countries for bacteria-antibiotic combinations, and we see little country-level patterns (Figure 10). As in, there is no trend for similar age-associations across bacteria-antibiotic within a country. The impact of age across different countries is correlated for some bacteria-antibiotic combinations, such as some *S. aureus* and *E. coli* resistances (Figure 11). We also look at the magnitude of the age effect across sub-regions and bacteria-antibiotic, by ranking each country by the magnitude of effect for each bacteria-antibiotic over ages 1-100 and summing across bacteria-antibiotic combinations. Investigating these by subregion show stark differences, with a higher magnitude effect of age in Southern / Eastern Europe than in Northern / Western Europe (Figure 12).


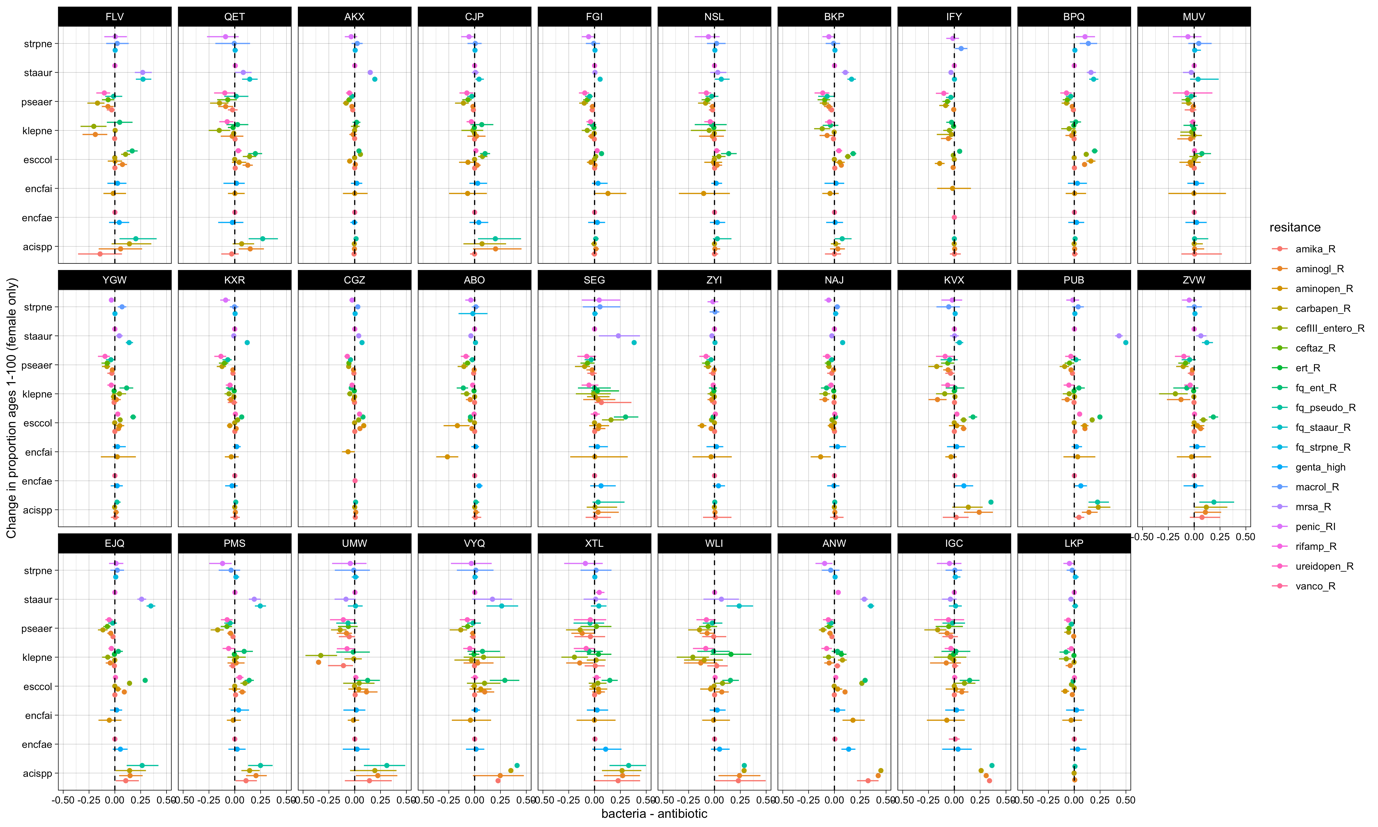


Figure 10: Country level age effect (difference in proportion resistant between ages 1 and 100 with 95% quantiles for females) by bacteria- antibiotic combination. Country labels are random anonymised three letter code used for this analysis only.


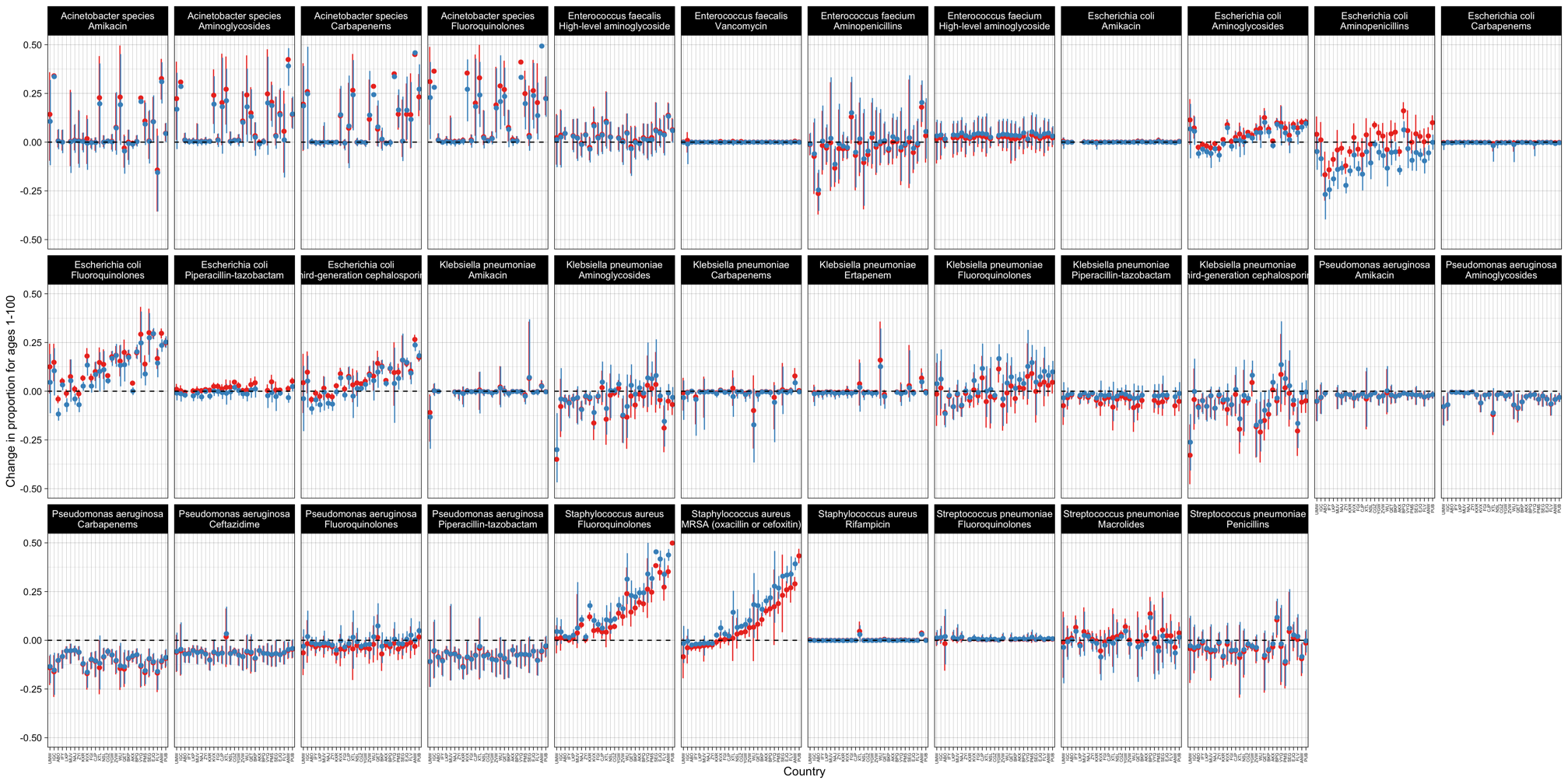

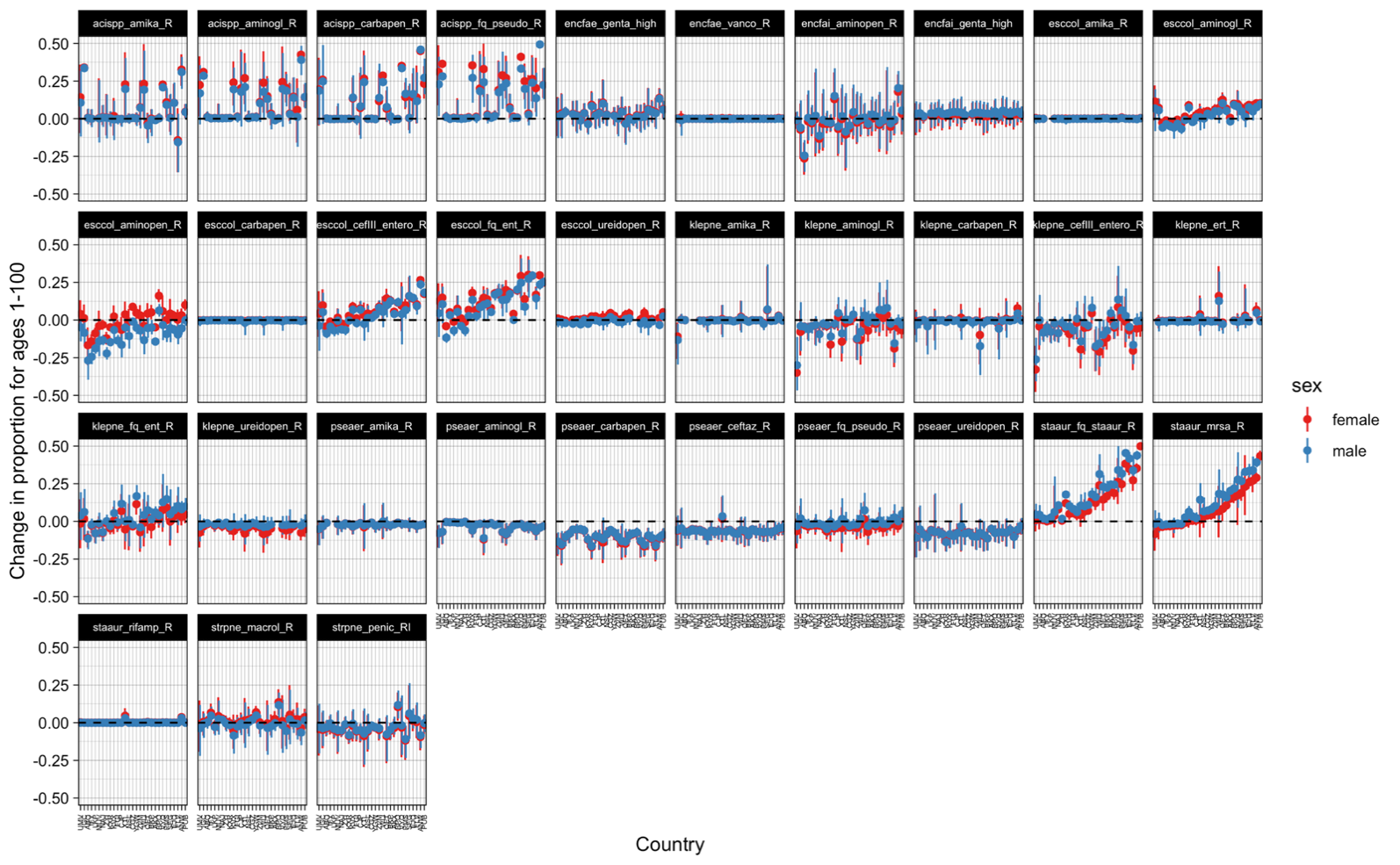


Figure 11: Changes in proportion for ages 1-100 by country (x axis) and sex (colour). Country labels are random anonymised three letter code used for this analysis only.


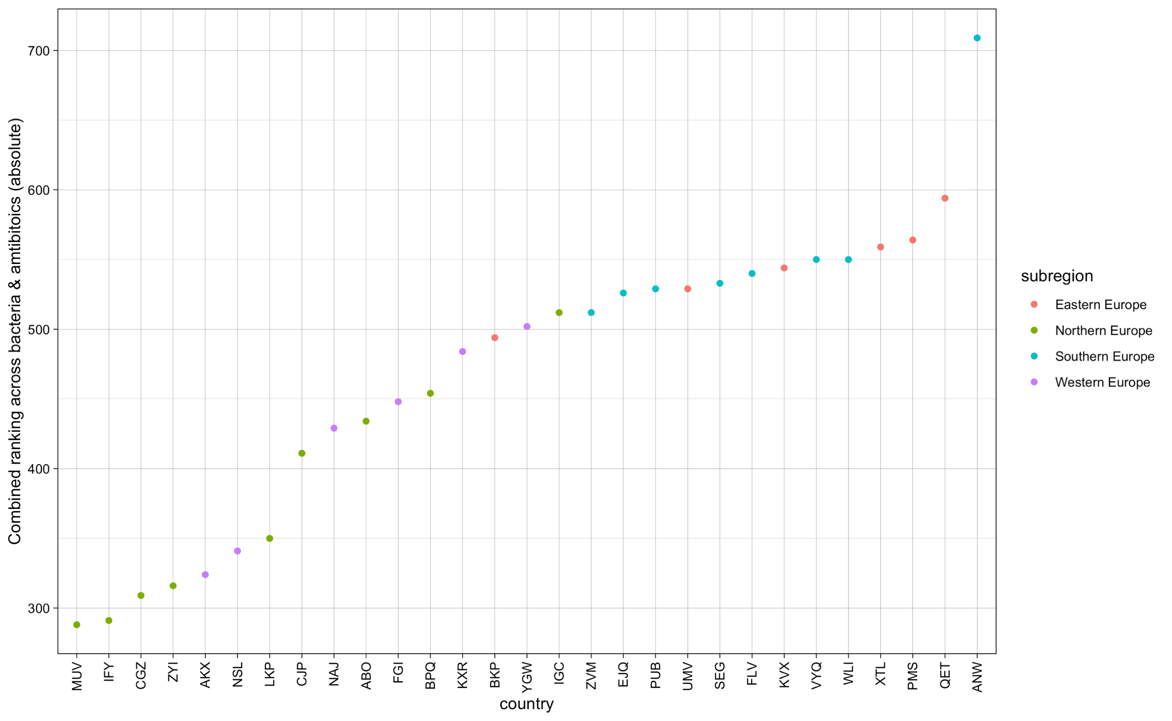


Figure 12: Combined ranking across bacteria-antibiotics for the magnitude of the age effects between ages 1 and 100. Country labels are random anonymised three letter code used for this analysis only.

## 6. Robustness of Index comparing resistance prevalence at ages 1 and 100

To cut through the complexity in the shape of the relationship between age and resistance prevalence, we wished to explore the main difference across antibiotics and bacteria over countries in terms of a general comparison of resistance prevalence in young vs. older people. Our index comparing the resistance prevalence in younger people (defined as 1yos) to older people (defined as 100yos) misses much of the complexity by not capturing that many are convex or concave.

To check the robustness of the index, we explored comparing the model predictions by sex of resistance prevalence at ages 50, 55, 60, 65, 70, 75, 80, 85, 90 and 95 against that at age 1 (Figure 6, Figure 8). We extracted the order of the countries (1 = smallest absolute median difference to 29 = highest absolute median difference) for each pairwise age comparison. This showed that the order of the countries varied little (colour remained constant for each country across y values) with some exceptions (country XTL for MRSA, and ICG for males for MRSA) (Figure 6). With the countries being anonymous this gives us little further information but suggests that those countries with higher resistance in BSI in those aged 100 also had higher resistance in those aged 50+ compared to those aged 1. The index was slightly less robust for MRSA than aminopenicillin resistance in *E. coli*.

We also explored varying the lower limit i.e. compared model predicted resistance prevalence by sex at age 100 to that at age 1, 5, 10, 15 and 20 (Figure 7, Figure 9). Similarly, this showed little variation in the order, with the exception of country KVX and XTL again for females in MRSA.

With decreases in the older age value (Figure 8) or increases in the younger age value (Figure 9), the absolute differences decreased as would be expected, but a subset of countries always have quantiles that do not cross zero.

Figure 6: The order of each country (colour) under a comparison of the size of the median difference in resistance prevalence by gender between age 10 and the age on the y axis for MRSA (top) and aminopenicillin resistance in E. coli (bottom). The x axis is ordered by the order of countries under the original comparison of resistance prevalence at age 100 vs age 1 for females.

Figure 7: The order of each country (colour) under a comparison of the size of the median difference in resistance prevalence by gender between age 100 and the age on the y axis for MRSA (top) and aminopenicillin resistance in E. coli (bottom). The x axis is ordered by the order of countries under the original comparison of resistance prevalence at age 100 vs age 1 for females.

Figure 8: Change in proportion resistant between ages 1 and the value at the top of each facet for each country and sex for MRSA (top) and aminopenicillin resistance in E coli (bottom), with the point indicating the median and the error bars the 95% quantiles across model predictions. Country labels are random anonymised three letter code used for this analysis only. The x axis is ordered by the order of countries under the original comparison of resistance prevalence at age 100 vs age 1 for females.

Figure 9: Change in proportion resistant between ages 100 and the value at the top of each facet for each country and sex for MRSA (top) and aminopenicillin resistance in E coli (bottom), with the point indicating the median and the error bars the 95% quantiles across model predictions. Country labels are random anonymised three letter code used for this analysis only. The x axis is ordered by the order of countries under the original comparison of resistance prevalence at age 100 vs age 1 for females.

## 7. Sensitivity analysis: data disaggregation by inpatient vs outpatient

*Background*

The original dataset includes a category of “Patient type” which recorded whether the patient at the moment the sample was taken was admitted in a hospital (“INPAT”, inpatient), or not (“OUTPAT”, outpatient). Patients that go to the hospital for dialysis or other types of day hospital care are classified as other (“o” in the data) or “unknown”.

This category could be used as a proxy for the important definition of community- vs hospital-acquired infection as the data is on the first blood and/or cerebrospinal fluid isolate from a patient. However, the standard definition splits the above classification based on the first 48hrs of patient care. Hence, some of the “inpatient” samples here will be officially “community acquired” (taken when the patient has officially been admitted but before they have been in hospital < 48hrs) [8]. We therefore cannot directly correlate this patient type with hospital vs community. Moreover, not all countries report this distinction – the UK has “unknown” for all isolates.

Exploring the data pooled across all countries with data, reveals the same age-dependent incidence curve for all bacteria across patient type where substantial data exists i.e. for inpatient and outpatient classifications and not “other” (Figure 13). Incidence is lower in outpatients as would be expected. Similar patterns can be seen for individual countries and across sexes as seen without the patient type split. However, this disaggregation also showed certain countries had substantially more isolates from outpatients. This likely reflects differences in healthcare processes and data coding.

*Conclusions*

The similarity of the high-income healthcare settings across Europe suggest that the above variations may be due to country level differences (“cultural factors”) in sampling and reporting rather than actual differences in bloodstream infection incidence by patient type. Hence, we have not explored resistance prevalence further by these patient types without further information on sample collection by country.

Figure 13: Impact of patient type (inpatient = INPAT, outpatient = OUTPAT or Other) on incidence of bloodstream infection for different bacterial types (colour) for 2019 for females (left) and males (right). Y axis is on a log scale (base 10).

## 8. Sensitivity analysis: data disaggregation by hospital unit type

*Background*

The dataset included a category of “Hospital unit type” which recorded the hospital unit within which the patient was when the sample was taken.

The Hospital Unit Types in data are:
ED = Emergency Department, ICU = Intensive Care Unit, INFECT = Infectious Disease Ward, INTMED = Internal Medicine, O = Other, OBGYN = Obstetrics/Gynecology, ONCOL = Haematology/Oncology, PEDS = Pediatrics/neonatal, PEDSICU = Pediatrics/neonatal ICU, PHC = Primary Health Care, SURG = Surgery, UNK = Unknown, URO = Urology Ward

*Data description*

Most were internal medicine (“INTMED”, 27%) or “Unknown” (21%), followed by Emergency Department (“ED”, 14%), the “ICU” (11%) and Surgery (“SURG”, 8%).

The distribution varied substantially by country – with one country have up to 53% of isolates reported as unknown, whilst another had the up to 45% as “Internal medicine” .

The age / sex distribution of incidence results across hospital unit types and further over countries are basically the same as across all data – there is an age association, with *E. coli* being the dominant bacteria (Figure 14). There seem some issues with the paediatric data (PEDS / PEDSICU) data with some isolates having older age associated with them. The data for oncology (“ONCOL”) suggests there may be some survival bias effects (Figure 14). *S. aureus* has the second highest infection incidence across all hospital unit types except for urology (“URO”) where other bacteria have relatively equal infection incidence levels which makes sense. The data from obstetrics and gynaecology (“OBGYN”) emphasises the importance of childbirth as a risk factor for women, though with relatively low rates.

The distribution of infection incidence emphases the above patterns showing for women aged 15-40 approximately 20% of the recorded bloodstream infections are linked to stays in the obstetrics and gynaecology departments (Figure 15). In men of this age, there is a larger contribution of infections in the ICU.

Figure 14: Total incidence of infection by age (x axis) and sex (columns) over all countries for different hospital unit types (rows) and bacteria (colours) in 2019. ED = Emergency Department, ICU = Intensive Care Unit, INFECT = Infectious Disease Ward, INTMED = Internal Medicine, O = Other, OBGYN = Obstetrics/Gynecology, ONCOL = Haematology/Oncology, PEDS = Pediatrics/neonatal, PEDSICU = Pediatrics/neonatal ICU, PHC = Primary Health Care, SURG = Surgery, UNK = Unknown, URO = Urology Ward. The y axis is on a log scale (base 10).

Figure 15: Distribution of infection incidence by hospital unit type (colour) across age (x axis) and sex (panels across Europe). ED = Emergency Department, ICU = Intensive Care Unit, INFECT = Infectious Disease Ward, INTMED = Internal Medicine, O = Other, OBGYN = Obstetrics/Gynecology, ONCOL = Haematology/Oncology, PEDS = Pediatrics/neonatal, PEDSICU = Pediatrics/neonatal ICU, PHC = Primary Health Care, SURG = Surgery, UNK = Unknown, URO = Urology Ward

*Conclusions*

The similarity of the high-income healthcare settings across Europe suggest that the above variations may be due to country level differences (“cultural factors”) in sampling and reporting rather than actual differences in bloodstream infection incidence by hospital unit type. Hence, we have not explored resistance prevalence further by these hospital unit types without further information on sample collection by country.

## 9. Sensitivity analysis: incidence

Using the minimum (instead of the actual reported or estimated coverage in 2019) estimates of the population coverage across the 2015-2019 data and estimates did not affect the sex and age patterns (Figure 16). The number of infections did increase substantially, as would be expected as coverage has been increasing for many countries, and also infections from countries that did not have coverage estimates for 2019 would be included if they had estimates for other years. On average, across all countries and years, the incidence was increased by ~38%, with similar increases for both sexes.

Figure 16: Incidence of bloodstream infections calculated using the minimum reported coverage estimates for surveillance per 100,000 population in 2019 across European countries for 8 bacterial pathogens split by (A) sex and bacteria for the first 50yrs of life and (B) sex (panel) and bacteria (colour) lifelong. Shaded areas are 95% confidence intervals using a LOESS fit. Infections in individuals younger than 0 are excluded, and those aged 80 and older are pooled into the 80yr data point. of bloodstream infections per 100,000 population per year across European countries for 8 bacterial pathogens (panel) for multiple years (colour) and split by sex (solid line = female, dashed line = male). Shaded areas are 95% confidence intervals using a LOESS fit.

## 10. Sensitivity Analysis: Priors

In our main paper models we used the default (flat) priors from the *brms* package [9] on all fixed effect variable parameters. In order to test the strength of our conclusions, we reran the MRSA model including regularising priors of *normal(0,1)* for all the fixed effect variables. We chose MRSA to run our sensitivity analysis, as this was one of the case studies in the main manuscript. Results showed no effect of the regularising priors on the estimated posterior parameters (Figure 17).


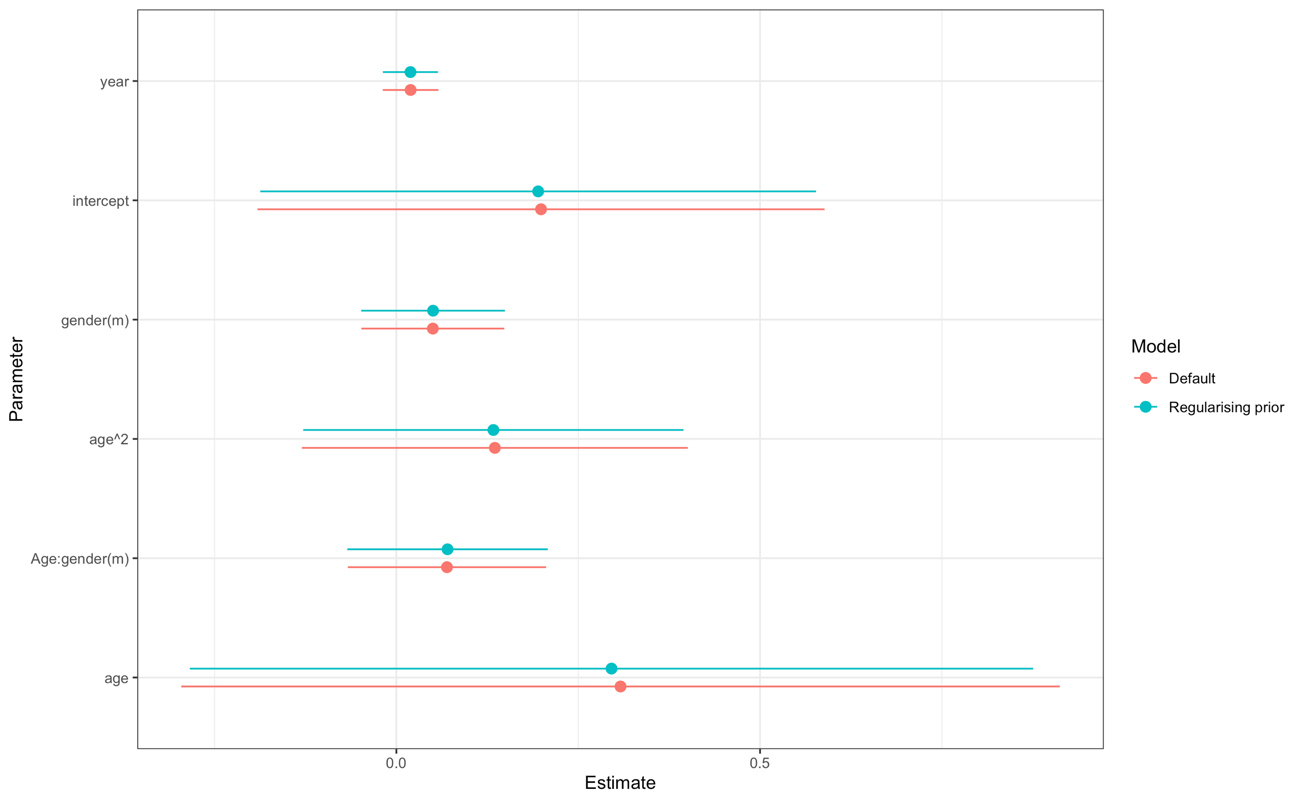


Figure 17: Parameter comparisons for sensitivity analysis including regularising priors. Points indicate the mean of the posterior estimate, with lines indicating the 95% credible interval.

## 11. Sensitivity Analysis: Aged 0

In our main analysis we excluded infants aged 0 from the data, due to large differences in their immune system and infection mechanisms. To test the impact of this, we reran the model for MRSA including individuals aged 0. We found no difference in the posterior parameter estimates for the fixed effect model variables (Figure 18A). Moreover, no substantial differences were seen in the random country level effects of age (Figure 18B).


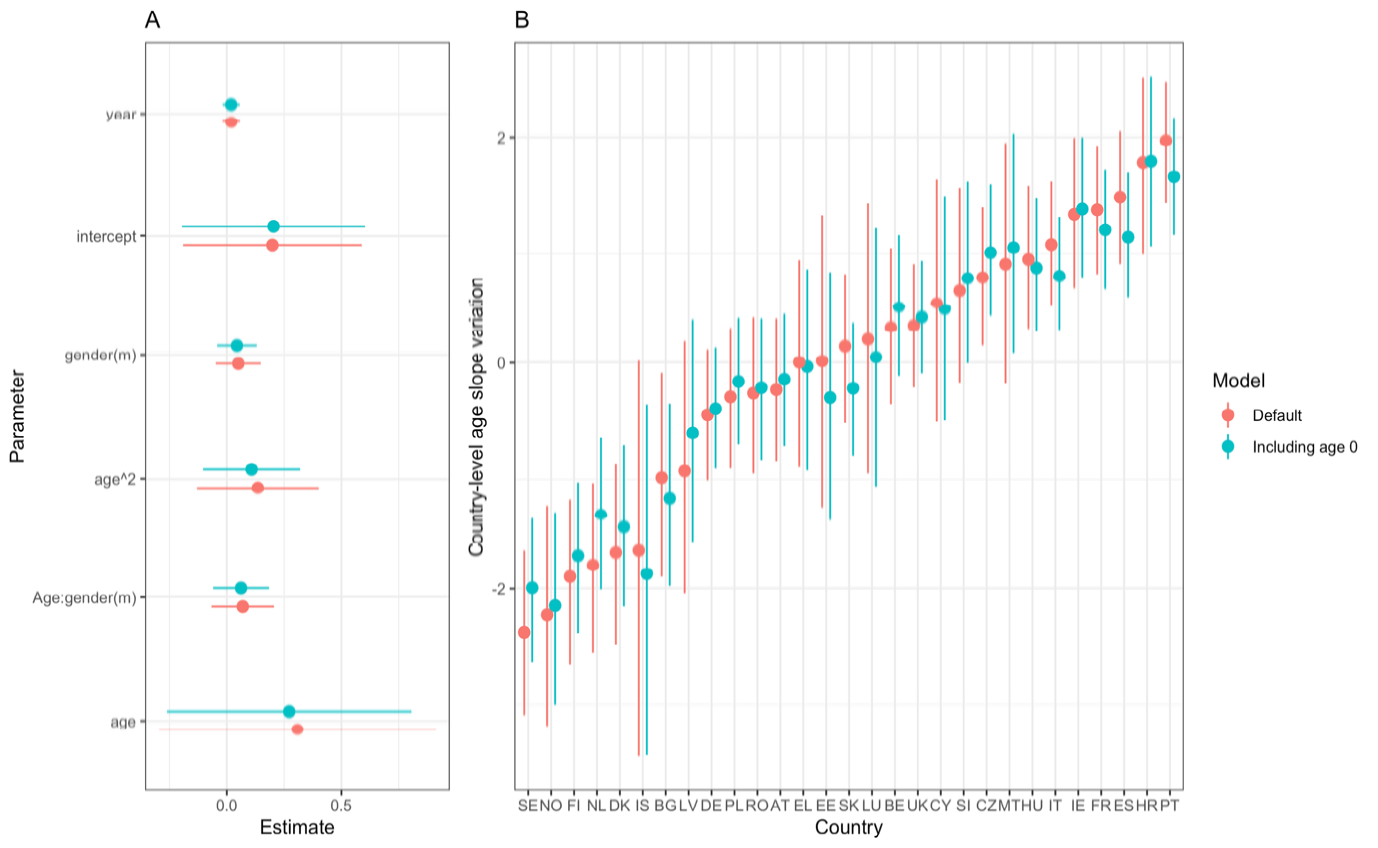


Figure 18: Parameter comparisons for sensitivity analysis including data from infants aged 0. Points indicate the mean of the posterior estimate, with lines indicating the 95% credible interval.

## 12. Sensitivity Analysis: Model structure

Whilst our main model used a Bayesian approach, where model variables that had no impact would have a parameter variable centred around 0, here we present a model comparison approach, for an example bacteria-antibiotic combination. In this approach, our base model (model A) did not account for sex or age impacts on resistance, but included a covariate for year, as well as random intercept effects for country and laboratory code (Equations 1:2). Our age model (model B) included additional covariates age, age-squared and a random covariate effect of age by country (Equations 3:4). This reflects the potential for age trends to be different across country due to differing policy and prescription patterns. Model C was the same as the base model, but additionally included a sex covariate (Equations 5:6). Model D was the complete model, including both age and sex terms, as in the main paper. Model E (Equations 7:8) was the complete model, without the age^2^ term to explore the importance of including this term. Differences between the base model and subsequent models are shown in bold.

| *Model A* | $\boldsymbol{y}_{\boldsymbol{i}}\boldsymbol{\sim Binomial(}\boldsymbol{n}_{\boldsymbol{i}}\boldsymbol{,}\boldsymbol{p}_{\boldsymbol{i}}\boldsymbol{)}$ | (1) |
| --- | --- | --- |
|  | $p_{i}=\beta_{0}+\beta_{t}*t_{i}+ v_{c(i)}+ u_{c(i),l(i)}+\epsilon_{i}$ | (2) |

Where $y$ is the resistance variable, taking a value of 0 or 1, $n$ is the number of samples and $p$ the probability of the sample being found to be resistant. The subscripts $c$, $l$ and $i$ denote country, laboratory code and grouping level. Recall that for each bacteria-antibiotic combination, our data consisted of multiple groupings of individual samples of a bacterium tested for resistance to that antibiotic. Each grouping *i* had a unique combination of country (c), laboratory code (l), sex, age and year of sample and hence a linked number of samples (*n*) and proportion resistant (*p*). $\beta_{0}$ is the overall intercept, $\beta_{t}$ is slope coefficient for time and $t$ is year. $\epsilon_{i}$ is the residual error, $u_{c(i),l(i)}$ is the level-2 random error on laboratory code and $v_{c(i)}$ is the level-3 random error on country.

| *Model B* | $\boldsymbol{y}_{\boldsymbol{i}}\boldsymbol{\sim Binomial(}\boldsymbol{n}_{\boldsymbol{i}}\boldsymbol{,}\boldsymbol{p}_{\boldsymbol{i}}\boldsymbol{)}$ | (3) |
| --- | --- | --- |
|  | $p_{i}=\beta_{0}+\beta_{t}*t_{i}\boldsymbol{+}\boldsymbol{\beta}_{\boldsymbol{a}}\boldsymbol{*}\boldsymbol{age}_{\boldsymbol{i}}\boldsymbol{+}\boldsymbol{\beta}_{\boldsymbol{a}^{\boldsymbol{2}}}\boldsymbol{*}{\boldsymbol{ag}\boldsymbol{e}^{\boldsymbol{2}}}_{\boldsymbol{i}}+ v_{c(i)}\boldsymbol{+}\boldsymbol{v}_{\boldsymbol{c(i)a(i)}}\boldsymbol{*}\boldsymbol{age}_{\boldsymbol{i}}+ u_{c(i),l(i)}+\epsilon_{i}$ | (4) |

Where $\beta_{a}$ is the age effect coefficient, $\beta_{a^{2}}$is the age squared effect coefficient and $\boldsymbol{v}_{\boldsymbol{ca}}$ is the country level age effect coefficient.

| *Model C* | $\boldsymbol{y}_{\boldsymbol{i}}\boldsymbol{\sim Binomial(}\boldsymbol{n}_{\boldsymbol{i}}\boldsymbol{,}\boldsymbol{p}_{\boldsymbol{i}}\boldsymbol{)}$ | (5) |
| --- | --- | --- |
|  | $p_{i}=\beta_{0}+\beta_{t}*t_{i}\boldsymbol{+}\boldsymbol{\beta}_{\boldsymbol{g}}\boldsymbol{*}\boldsymbol{gender}_{\boldsymbol{i}}+ v_{c(i)}+ u_{c(i),l(i)}+\epsilon_{i}$ | (6) |

Where $\beta_{g}$ is the sex effect coefficient.

| *Model D* | $\boldsymbol{y}_{\boldsymbol{i}}\boldsymbol{\sim Binomial(}\boldsymbol{n}_{\boldsymbol{i}}\boldsymbol{,}\boldsymbol{p}_{\boldsymbol{i}}\boldsymbol{)}$ | (7) |
| --- | --- | --- |
|  | $p_{i}=\beta_{0}+\beta_{t}*t_{i}+\boldsymbol{\beta}_{\boldsymbol{a}}\boldsymbol{*}\boldsymbol{age}_{\boldsymbol{i}}+ \boldsymbol{\beta}_{\boldsymbol{a}^{\boldsymbol{2}}}\boldsymbol{*}{\boldsymbol{ag}\boldsymbol{e}^{\boldsymbol{2}}}_{\boldsymbol{i}}+ \beta_{g}*{sex}_{i}+ \boldsymbol{\beta}_{\boldsymbol{ag}}\boldsymbol{*}\boldsymbol{age}_{\boldsymbol{i}}\boldsymbol{*}\boldsymbol{sex}_{\boldsymbol{i}}+ v_{c(i)}+\boldsymbol{v}_{\boldsymbol{c(i)a(i)}}\boldsymbol{*}\boldsymbol{age}_{\boldsymbol{i}}+ u_{c(i),l(i)}+\epsilon_{i}$ | (8) |

Where $\beta_{ag}$ is the sex and age interaction coefficient.

| *Model E* | $\boldsymbol{y}_{\boldsymbol{c,l,i}}\boldsymbol{\sim Binomial(}\boldsymbol{n}_{\boldsymbol{c,l,i}}\boldsymbol{,}\boldsymbol{p}_{\boldsymbol{c,l,i}}\boldsymbol{)}$ | (7) |
| --- | --- | --- |
|  | $p_{c,l,i}=\beta_{0}+\beta_{t}*t_{i}\boldsymbol{+}\boldsymbol{\beta}_{\boldsymbol{a}}\boldsymbol{*}\boldsymbol{age}_{\boldsymbol{i}}+ \boldsymbol{\beta}_{\boldsymbol{g}}\boldsymbol{*}\boldsymbol{gender}_{\boldsymbol{i}}\boldsymbol{+}\boldsymbol{\beta}_{\boldsymbol{ag}}\boldsymbol{*}\boldsymbol{age*gender}_{\boldsymbol{i}}\boldsymbol{+}v_{c}\boldsymbol{+}\boldsymbol{v}_{\boldsymbol{ca}}\boldsymbol{*}\boldsymbol{age}_{\boldsymbol{i}}+ u_{c,l}+\epsilon_{c,l,i}$ | (8) |

We ran this set of models for MRSA (coefficient estimates in Figure 19A). Leave-one-out cross validation (approximated by the Pareto smoothed importance-sampling method) (LOOIC) and the widely applicable information criterion (WAIC) were used to compare the predictive ability of the models.

Due to model nesting, we compared A-B-D-E and A-C-D-E separately. Including age in the base model A had a large impact on the LOOIC (Figure 19B, Model B vs Model A), and adding sex on top of that resulted an additional small improvement (Model D vs Model B). However, adding sex to the base model did not improve the LOOIC (Figure 19C, Model C vs Model A). Adding in age as well as sex had significant improvements on the LOOIC (Model D vs Model C). The inclusion of the age^2^ term (Figure 15 Model E vs B) had an additional small improvement on the LOOIC vs a single age term.

These results indicate that Model D, the one used in our main analysis, would be chosen as the best model to use in the model comparison approach for MRSA.


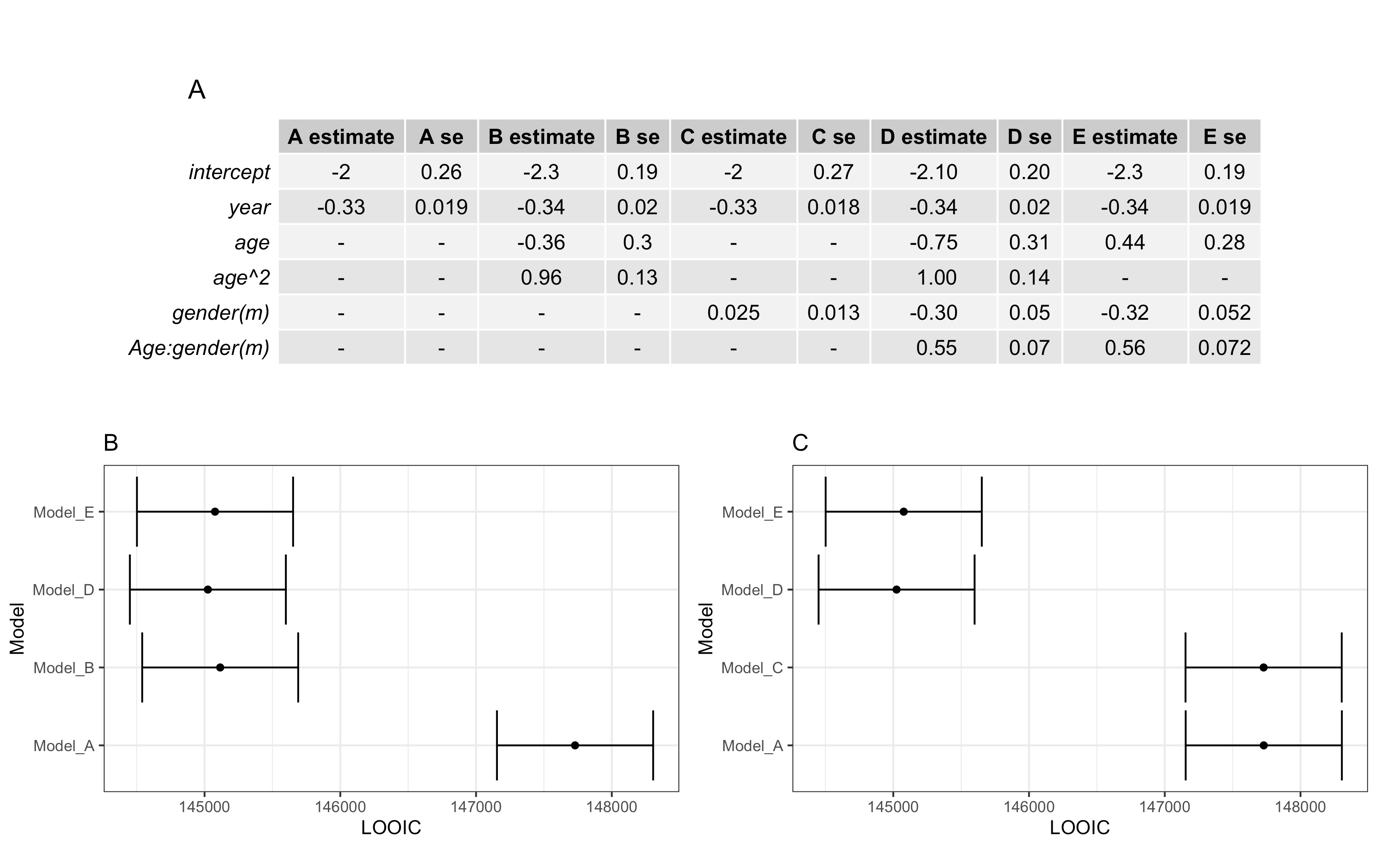


Figure 19: Model structure sensitivity analysis. A) Comparison of posterior parameter values across models. B) LOOIC for nested models with age. C) LOOIC for nested models with gender.

**References**

1. Central Asian and European surveillance of Antimicrobial Resistance (CAESAR), European Antimicrobial Resistance Surveillance Network (EARS-Net). Antimicrobial resistance surveillance in Europe - 2023. 2023 Apr. Available from: https://www.ecdc.europa.eu/en/publications-data/antimicrobial-resistance-surveillance-europe-2023

2. Gagliotti C, Högberg LD, Billström H, Eckmanns T, Giske CG, Heuer OE, et al. Staphylococcus aureus bloodstream infections: diverging trends of meticillin-resistant and meticillin-susceptible isolates, EU/EEA, 2005 to 2018. Eurosurveillance. 2021;26: 2002094. doi:10.2807/1560-7917.ES.2021.26.46.2002094

3. de Kraker MEA, Jarlier V, Monen JCM, Heuer OE, van de Sande N, Grundmann H. The changing epidemiology of bacteraemias in Europe: trends from the European Antimicrobial Resistance Surveillance System. Clin Microbiol Infect. 2013;19: 860–868. doi:10.1111/1469-0691.12028

4. Piezzi V, Gasser M, Atkinson A, Kronenberg A, Vuichard-Gysin D, Harbarth S, et al. Increasing proportion of vancomycin-resistance among enterococcal bacteraemias in Switzerland: a 6-year nation-wide surveillance, 2013 to 2018. Euro Surveill. 2020;25. doi:10.2807/1560-7917.ES.2020.25.35.1900575

5. Buetti N, Atkinson A, Marschall J, Kronenberg A, Swiss Centre for Antibiotic Resistance (ANRESIS). Incidence of bloodstream infections: a nationwide surveillance of acute care hospitals in Switzerland 2008-2014. BMJ Open. 2017;7: e013665. doi:10.1136/bmjopen-2016-013665

6. Reacher MH, Shah A, Livermore DM, Wale MC, Graham C, Johnson AP, et al. Bacteraemia and antibiotic resistance of its pathogens reported in England and Wales between 1990 and 1998: trend analysis. BMJ. 2000;320: 213–216. doi:10/fc4qm9

7. Wilson J, Elgohari S, Livermore DM, Cookson B, Johnson A, Lamagni T, et al. Trends among pathogens reported as causing bacteraemia in England, 2004-2008. Clin Microbiol Infect. 2011;17: 451–458. doi:10.1111/j.1469-0691.2010.03262.x

8. Garner JS, Jarvis WR, Emori TG, Horan TC, Hughes JM. CDC definitions for nosocomial infections, 1988. Am J Infect Control. 1988;16: 128–140. doi:10.1016/0196-6553(88)90053-3

9. Bürkner P-C. brms: An *R* Package for Bayesian Multilevel Models Using Stan. J Stat Softw. 2017;80: 1–28. doi:10.18637/jss.v080.i01
