# Supplementary material for: Antimicrobial resistance prevalence in bloodstream infection in 29 European countries by age and sex: An observational study
Source: PLoS Med. 2024 Mar 14;21(3):e1004301. doi: 10.1371/journal.pmed.1004301 (PMC10939247; doi:10.1371/journal.pmed.1004301)
Supplement: S3 Appendix — (PDF) [file pmed.1004301.s003.pdf]

S3 Appendix

Naomi R Waterlow, Ben S Cooper, Julie V Robotham, Gwenan M Knight

2024-01-29

Paper: Antimicrobial resistance prevalence in bloodstream infection in 29 European countries by age and sex: an observational study

Bacteria-antibiotic specific results

This Appendix contains the model results for each bacteria-antibiotic combination.

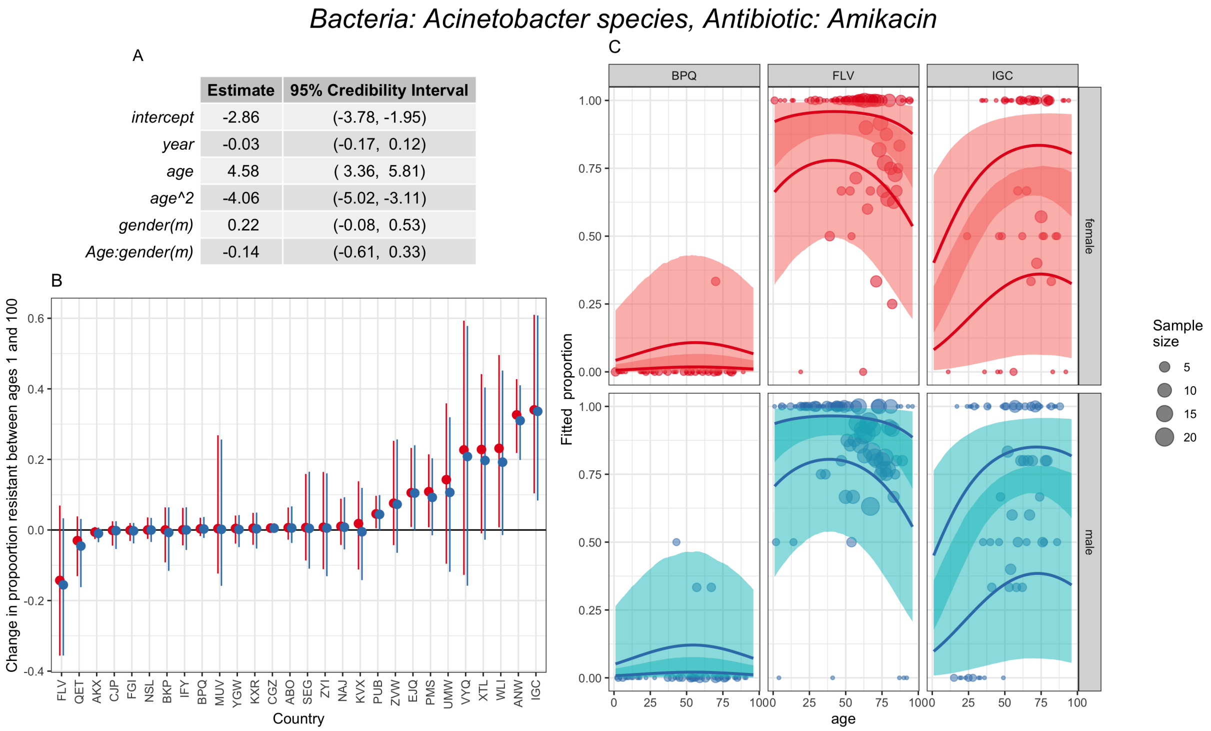

	Estimate	95% Credibility Interval
intercept	-2.86	(-3.78, -1.95)
year	-0.03	(-0.17, 0.12)
age	4.58	(3.36, 5.81)
age^2	-4.06	(-5.02, -3.11)
gender(m)	0.22	(-0.08, 0.53)
Age:gender(m)	-0.14	(-0.61, 0.33)

Figure 1: *Acinetobacter*, Amikacin A) Model parameters. B) Change in proportion resistant between ages 1 and 100 for each country and sex. C) Data (points) and model predictions (lines) with 95% CIs (ribbons) for the most extreme and the middle county from B. Each country has two lines, depicting the predictions for the most extreme laboratories in the country. Data is grouped across years and laboratories.

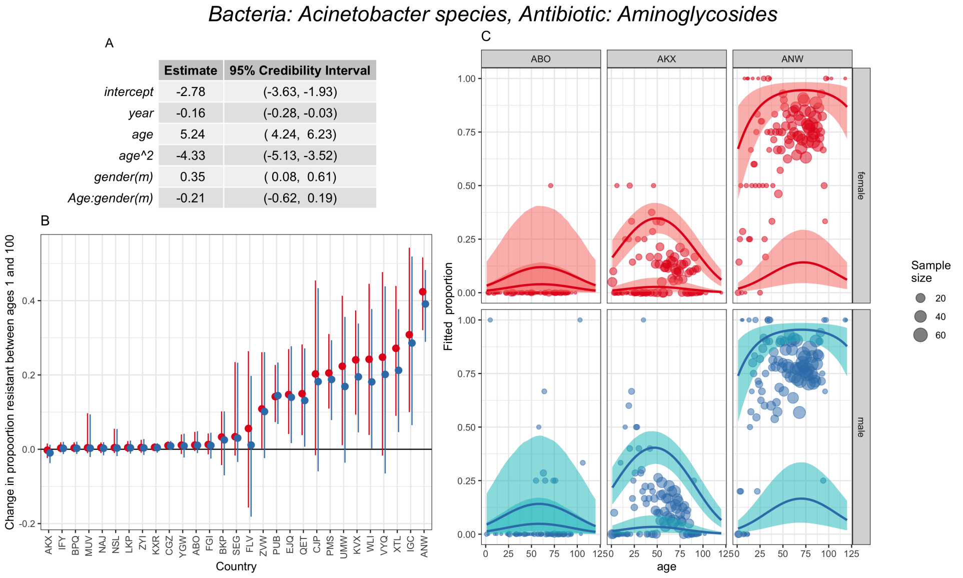

Figure 2: *Acinetobacter*, Aminoglycosides A) Model parameters. B) Change in proportion resistant between ages 1 and 100 for each country and sex. C) Data (points) and model predictions (lines) with 95% CIs (ribbons) for the most extreme and the middle county from B. Each country has two lines, depicting the predictions for the most extreme laboratories in the country. Data is grouped across years and laboratories.

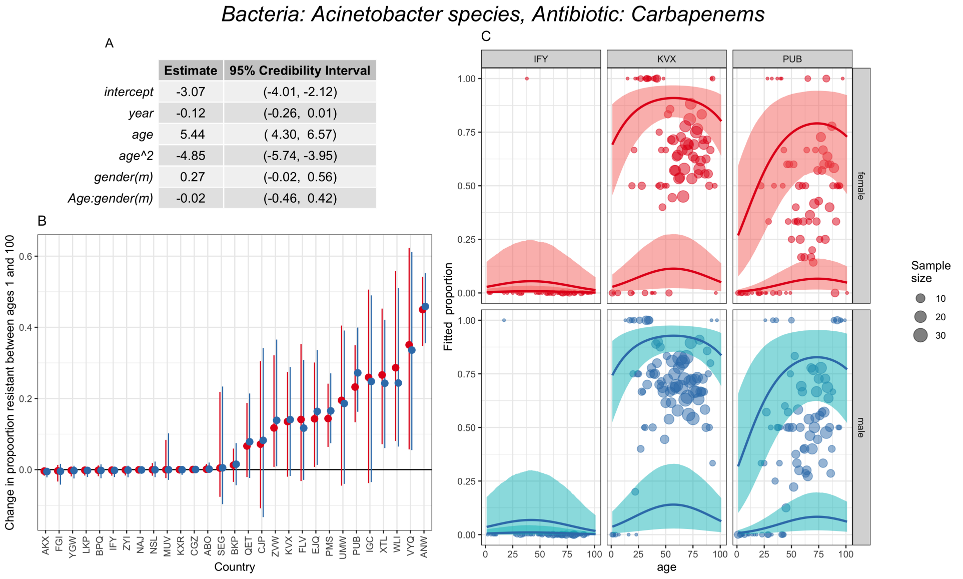

Figure 3: *Acinetobacter*, Carbapenems A) Model parameters. B) Change in proportion resistant between ages 1 and 100 for each country and sex. C) Data (points) and model predictions (lines) with 95% CIs (ribbons) for the most extreme and the middle county from B. Each country has two lines, depicting the predictions for the most extreme laboratories in the country. Data is grouped across years and laboratories.

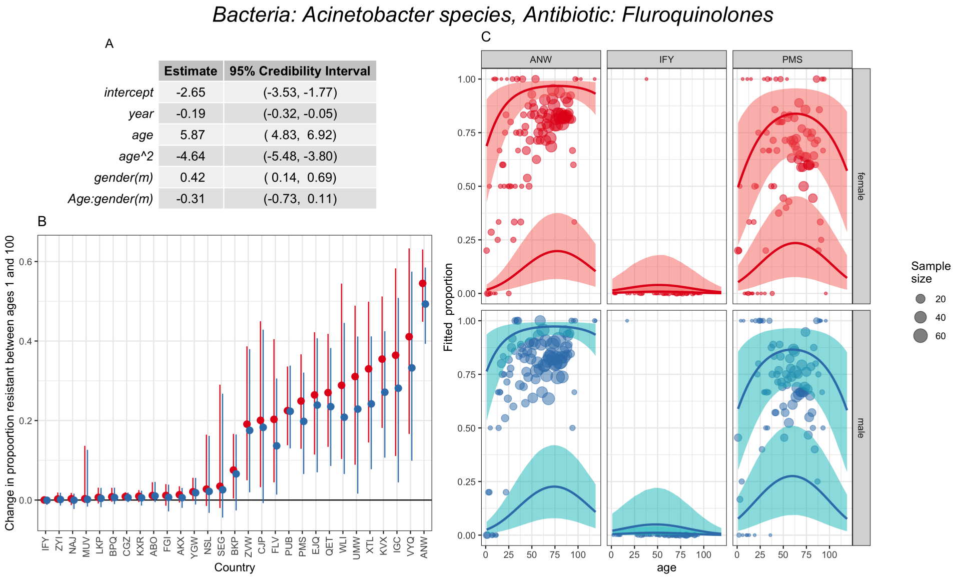

Figure 4: *Acinetobacter*, Fluroquinolones A) Model parameters. B) Change in proportion resistant between ages 1 and 100 for each country and sex. C) Data (points) and model predictions (lines) with 95% CIs (ribbons) for the most extreme and the middle county from B. Each country has two lines, depicting the predictions for the most extreme laboratories in the country. Data is grouped across years and laboratories.

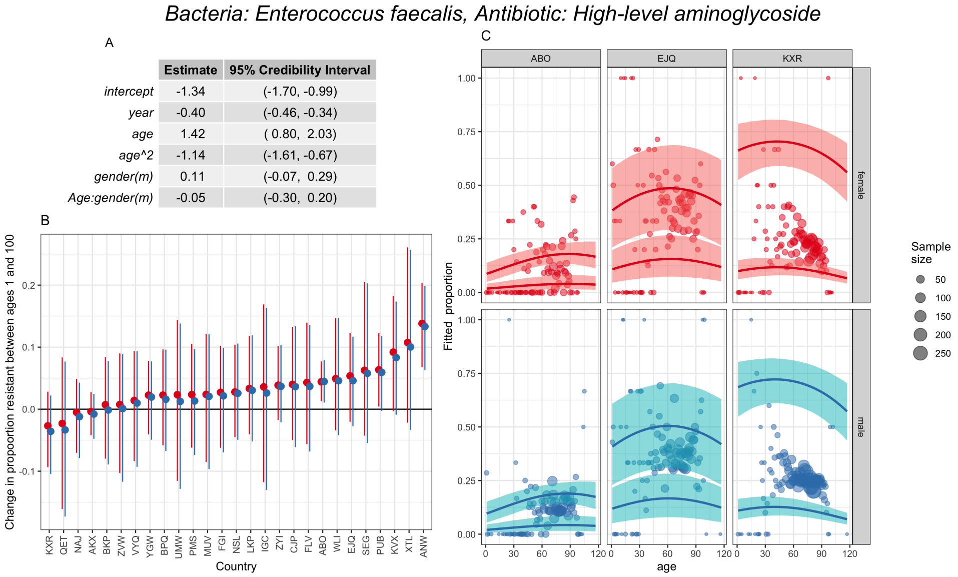

Figure 5: *Enterococcus faecalis*, High-level aminoglycoside A) Model parameters. B) Change in proportion resistant between ages 1 and 100 for each country and sex. C) Data (points) and model predictions (lines) with 95% CIs (ribbons) for the most extreme and the middle county from B. Each country has two lines, depicting the predictions for the most extreme laboratories in the country. Data is grouped across years and laboratories.

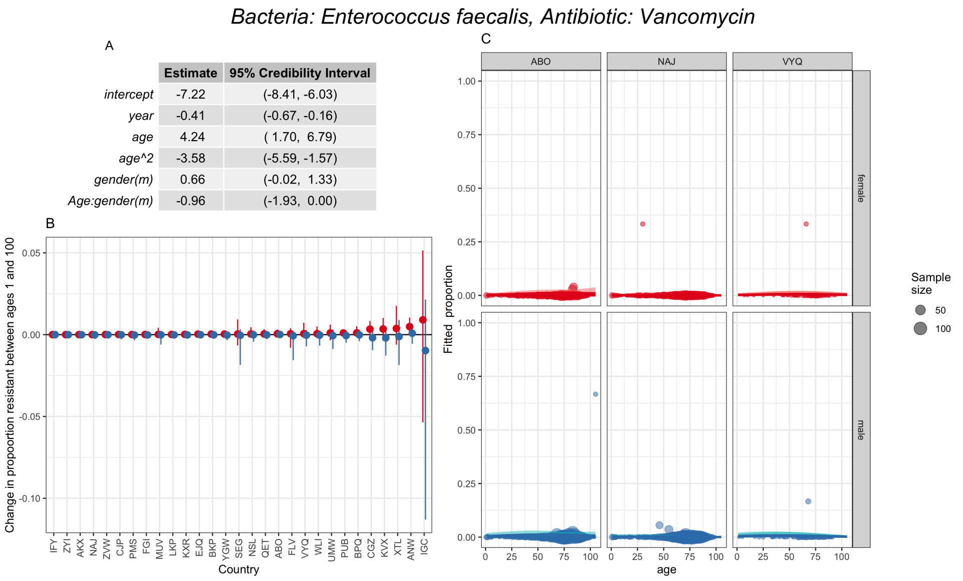

	Estimate	95% Credibility Interval
intercept	-7.22	(-8.41, -6.03)
year	-0.41	(-0.67, -0.16)
age	4.24	(1.70, 6.79)
age^2	-3.58	(-5.59, -1.57)
gender(m)	0.66	(-0.02, 1.33)
Age:gender(m)	-0.96	(-1.93, 0.00)

Figure 6: *Enterococcus faecalis*, Vancomycin. A) Model parameters. B) Change in proportion resistant between ages 1 and 100 for each country and sex. C) Data (points) and model predictions (lines) with 95% CIs (ribbons) for the most extreme and the middle county from B. Each country has two lines, depicting the predictions for the most extreme laboratories in the country. Data is grouped across years and laboratories.

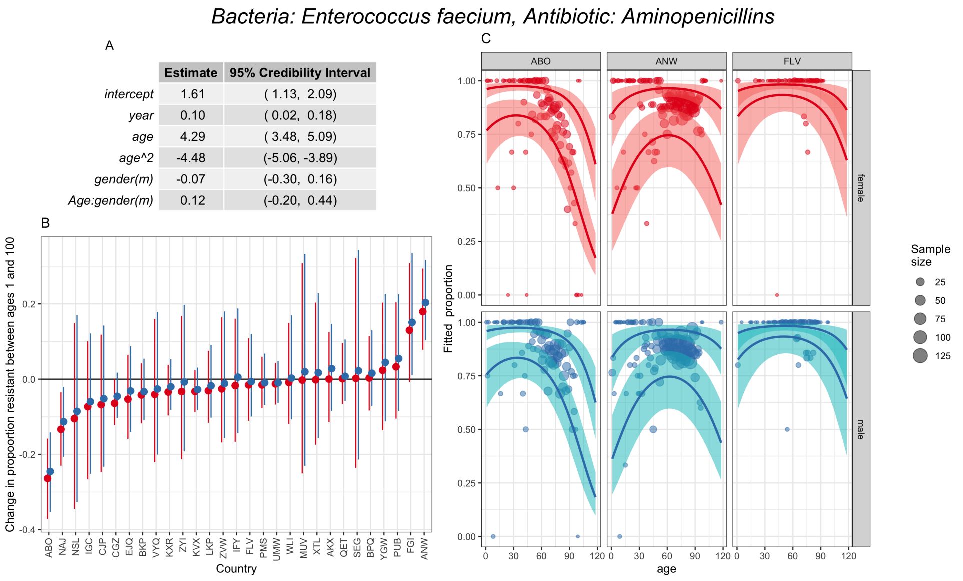

	Estimate	95% Credibility Interval
intercept	1.61	(1.13, 2.09)
year	0.10	(0.02, 0.18)
age	4.29	(3.48, 5.09)
age^2	-4.48	(-5.06, -3.89)
gender(m)	-0.07	(-0.30, 0.16)
Age:gender(m)	0.12	(-0.20, 0.44)

Figure 7: *Enterococcus faecium*, Aminopenicillins. A) Model parameters. B) Change in proportion resistant between ages 1 and 100 for each country and sex. C) Data (points) and model predictions (lines) with 95% CIs (ribbons) for the most extreme and the middle county from B. Each country has two lines, depicting the predictions for the most extreme laboratories in the country. Data is grouped across years and laboratories.

Bacteria: Enterococcus faecium, Antibiotic: High-level aminoglycoside

A

	Estimate	95% Credibility Interval
intercept	-0.59	(-1.02, -0.15)
year	-0.33	(-0.40, -0.25)
age	2.12	(1.39, 2.85)
age^2	-1.97	(-2.56, -1.38)
gender(m)	-0.04	(-0.24, 0.17)
Age:gender(m)	0.07	(-0.22, 0.36)

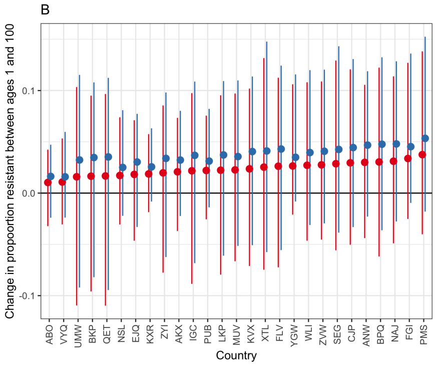

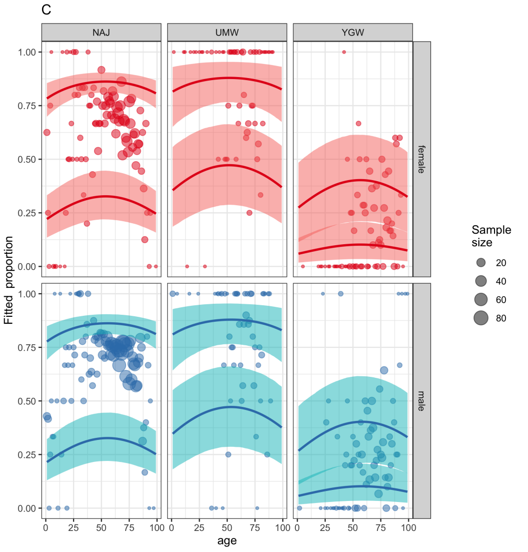

Figure 8: *Enterococcus faecium*, High-level aminoglycoside A) Model parameters. B) Change in proportion resistant between ages 1 and 100 for each country and sex. C) Data (points) and model predictions (lines) with 95% CIs (ribbons) for the most extreme and the middle county from B. Each country has two lines, depicting the predictions for the most extreme laboratories in the country. Data is grouped across years and laboratories.

Bacteria: Escherichia coli, Antibiotic: Amikacin

A

	Estimate	95% Credibility Interval
intercept	-5.31	(-5.85, -4.77)
year	0.04	(-0.08, 0.17)
age	-0.95	(-2.04, 0.14)
age^2	1.10	(0.25, 1.95)
gender(m)	0.45	(0.12, 0.78)
Age:gender(m)	-0.21	(-0.67, 0.24)

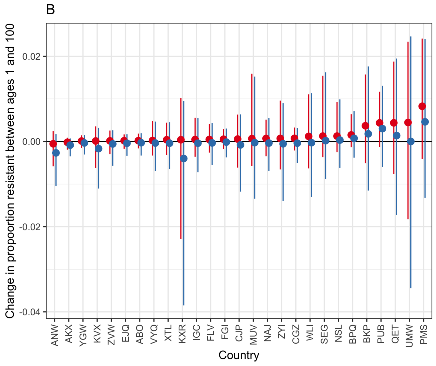

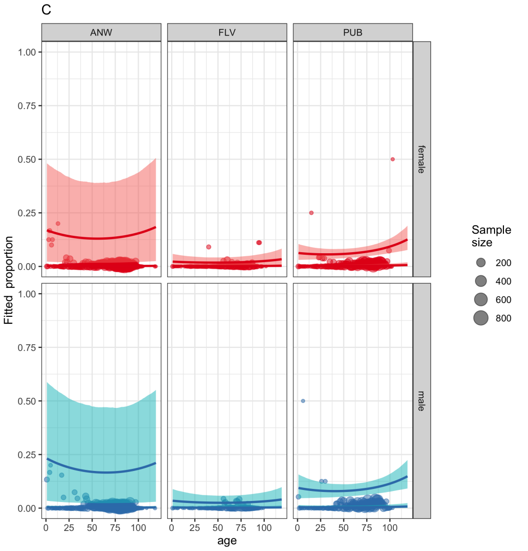

Figure 9: *Escherichia coli*, Amikacin. A) Model parameters. B) Change in proportion resistant between ages 1 and 100 for each country and sex. C) Data (points) and model predictions (lines) with 95% CIs (ribbons) for the most extreme and the middle county from B. Each country has two lines, depicting the predictions for the most extreme laboratories in the country. Data is grouped across years and laboratories.

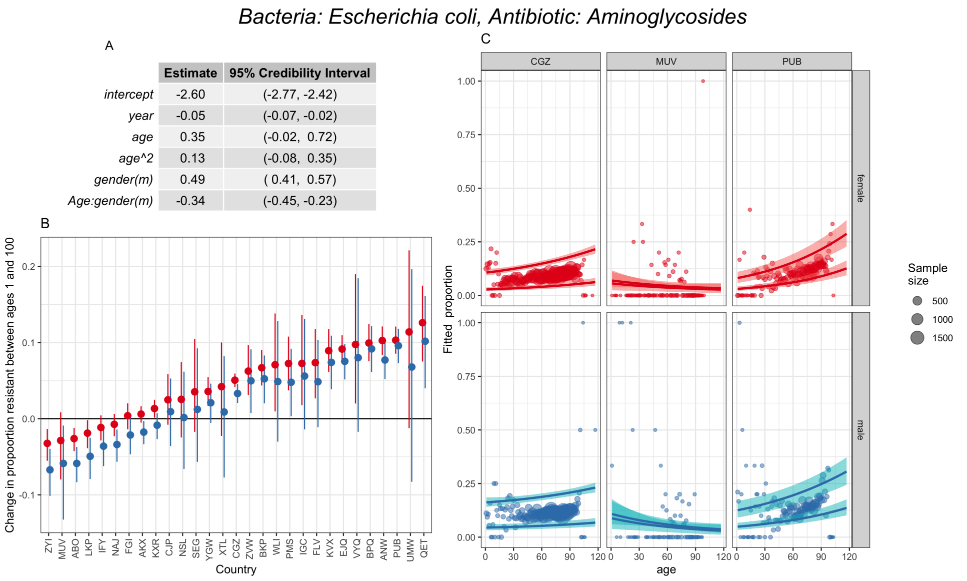

Figure 10: *Escherichia coli*, Aminoglycosides. A) Model parameters. B) Change in proportion resistant between ages 1 and 100 for each country and sex. C) Data (points) and model predictions (lines) with 95% CIs (ribbons) for the most extreme and the middle county from B. Each country has two lines, depicting the predictions for the most extreme laboratories in the country. Data is grouped across years and laboratories.

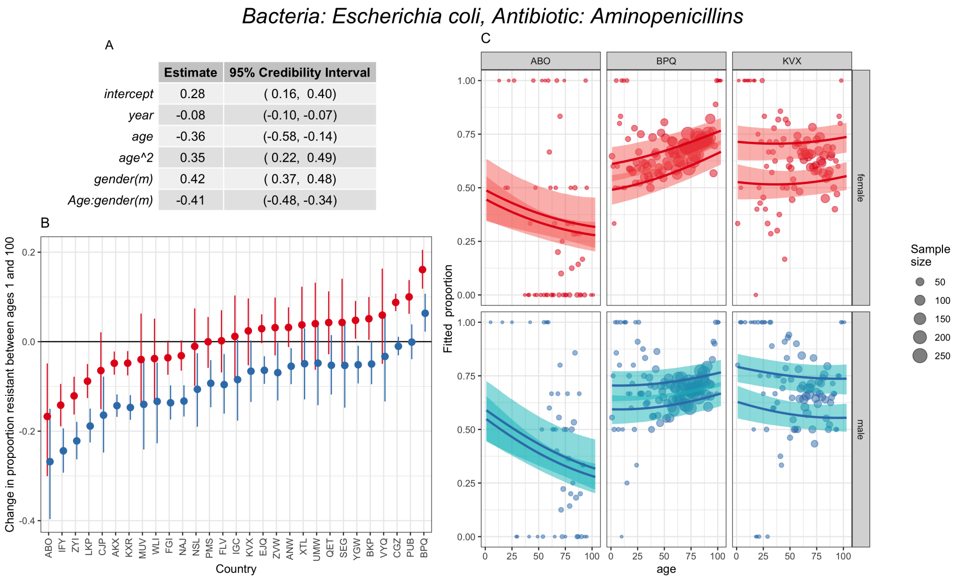

Figure 11: *Escherichia coli*, Aminopenicillins A) Model parameters. B) Change in proportion resistant between ages 1 and 100 for each country and sex. C) Data (points) and model predictions (lines) with 95% CIs (ribbons) for the most extreme and the middle county from B. Each country has two lines, depicting the predictions for the most extreme laboratories in the country. Data is grouped across years and laboratories.

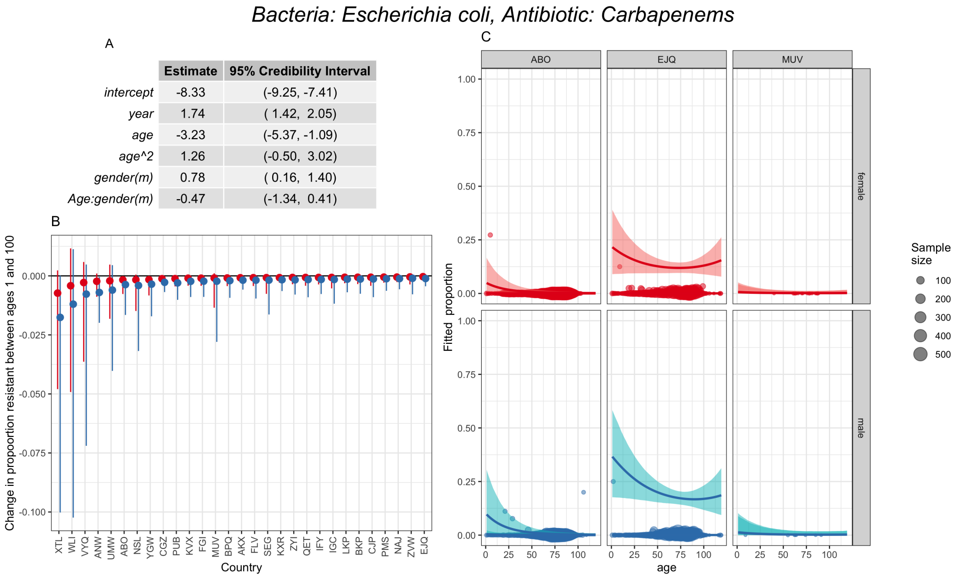

	Estimate	95% Credibility Interval
intercept	-8.33	(-9.25, -7.41)
year	1.74	(1.42, 2.05)
age	-3.23	(-5.37, -1.09)
age^2	1.26	(-0.50, 3.02)
gender(m)	0.78	(0.16, 1.40)
Age:gender(m)	-0.47	(-1.34, 0.41)

Figure 12: *Escherichia coli*, Carbapenems. A) Model parameters. B) Change in proportion resistant between ages 1 and 100 for each country and sex. C) Data (points) and model predictions (lines) with 95% CIs (ribbons) for the most extreme and the middle county from B. Each country has two lines, depicting the predictions for the most extreme laboratories in the country. Data is grouped across years and laboratories.

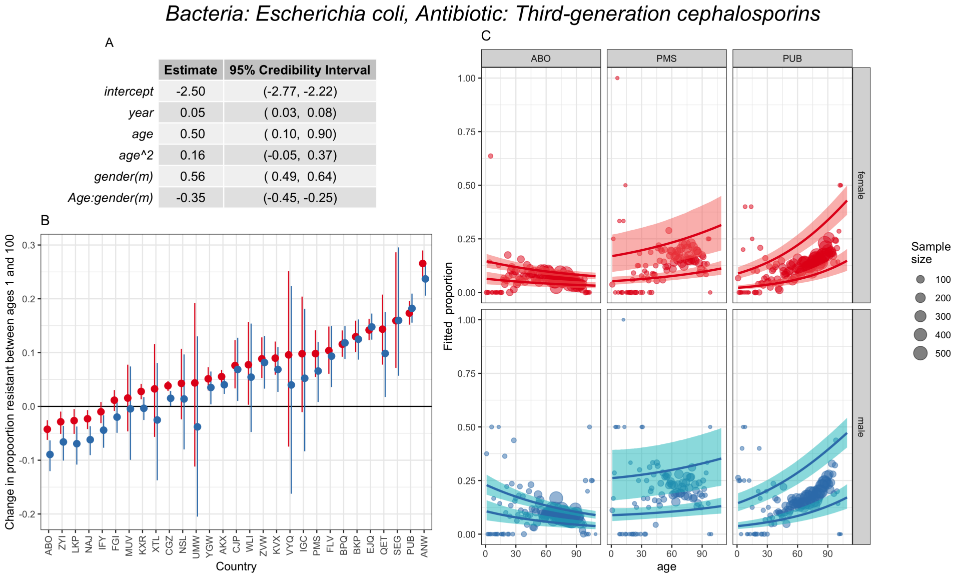

	Estimate	95% Credibility Interval
intercept	-2.50	(-2.77, -2.22)
year	0.05	(0.03, 0.08)
age	0.50	(0.10, 0.90)
age^2	0.16	(-0.05, 0.37)
gender(m)	0.56	(0.49, 0.64)
Age:gender(m)	-0.35	(-0.45, -0.25)

Figure 13: *Escherichia coli*, Third-generation cephalosporins. A) Model parameters. B) Change in proportion resistant between ages 1 and 100 for each country and sex. C) Data (points) and model predictions (lines) with 95% CIs (ribbons) for the most extreme and the middle county from B. Each country has two lines, depicting the predictions for the most extreme laboratories in the country. Data is grouped across years and laboratories.

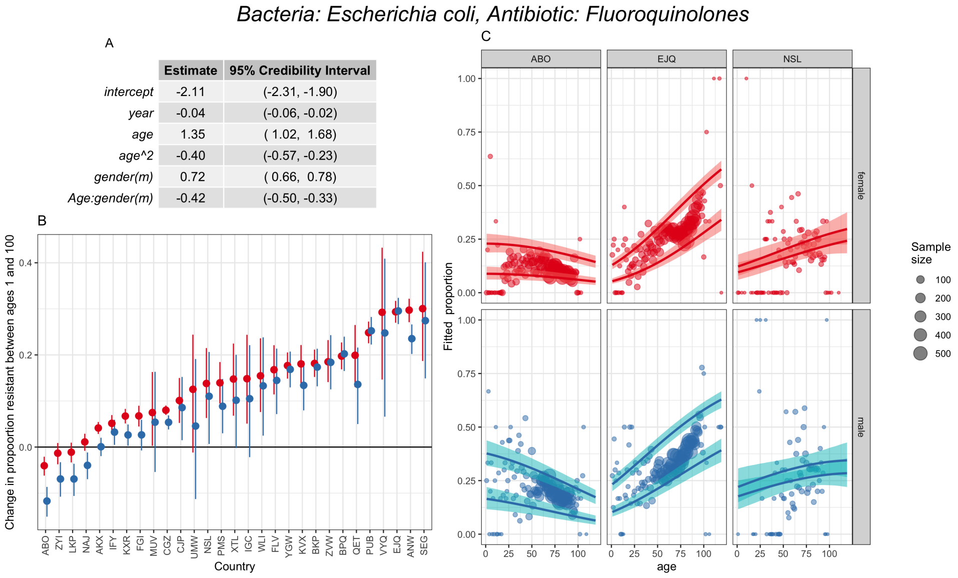

Figure 14: *Escherichia coli*, Fluoroquinolones. A) Model parameters. B) Change in proportion resistant between ages 1 and 100 for each country and sex. C) Data (points) and model predictions (lines) with 95% CIs (ribbons) for the most extreme and the middle county from B. Each country has two lines, depicting the predictions for the most extreme laboratories in the country. Data is grouped across years and laboratories.

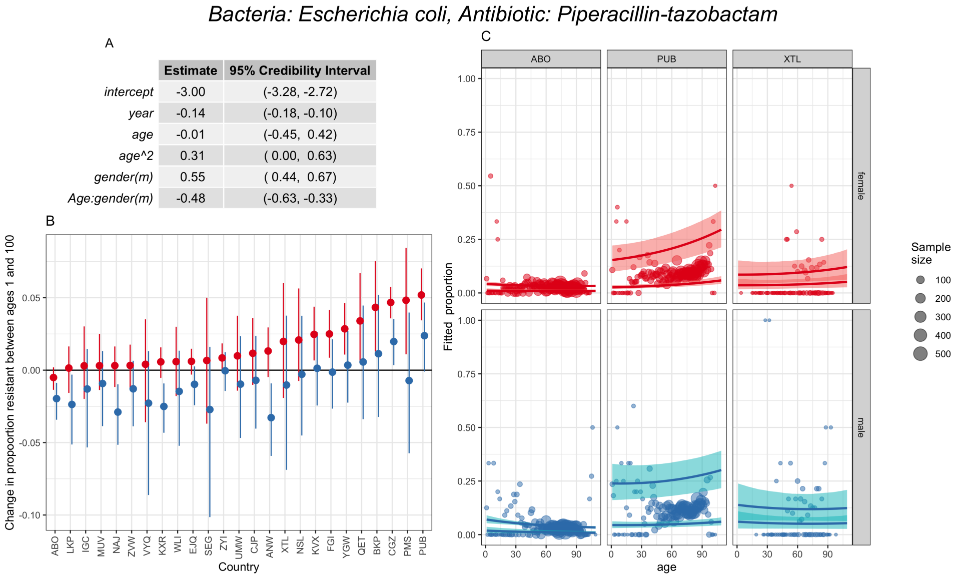

Figure 15: *Escherichia coli*, piperacillin-tazobactam A) Model parameters. B) Change in proportion resistant between ages 1 and 100 for each country and sex. C) Data (points) and model predictions (lines) with 95% CIs (ribbons) for the most extreme and the middle county from B. Each country has two lines, depicting the predictions for the most extreme laboratories in the country. Data is grouped across years and laboratories.

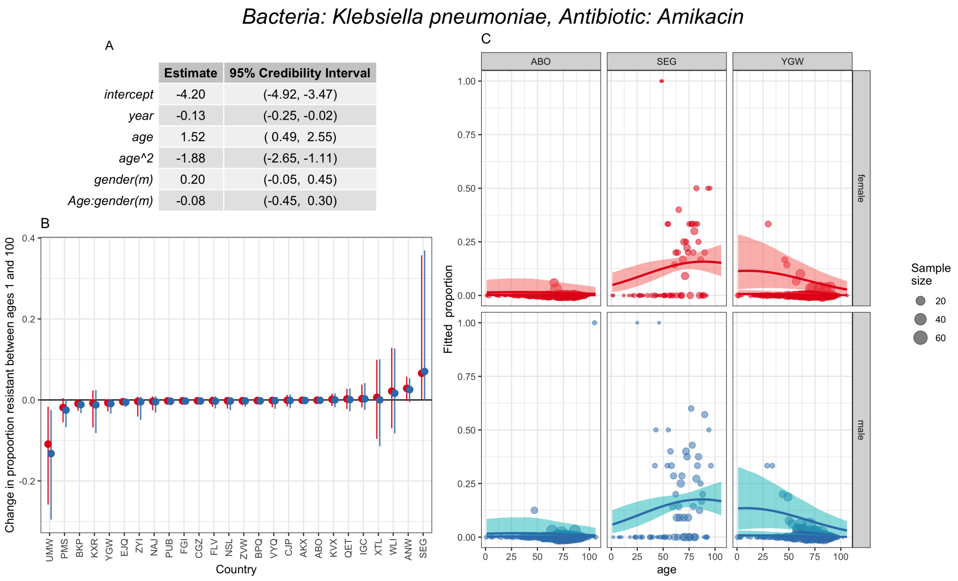

	Estimate	95% Credibility Interval
intercept	-4.20	(-4.92, -3.47)
year	-0.13	(-0.25, -0.02)
age	1.52	(0.49, 2.55)
age^2	-1.88	(-2.65, -1.11)
gender(m)	0.20	(-0.05, 0.45)
Age:gender(m)	-0.08	(-0.45, 0.30)

Figure 16: *Klebsiella pneumoniae*, Amikacin. A) Model parameters. B) Change in proportion resistant between ages 1 and 100 for each country and sex. C) Data (points) and model predictions (lines) with 95% CIs (ribbons) for the most extreme and the middle county from B. Each country has two lines, depicting the predictions for the most extreme laboratories in the country. Data is grouped across years and laboratories.

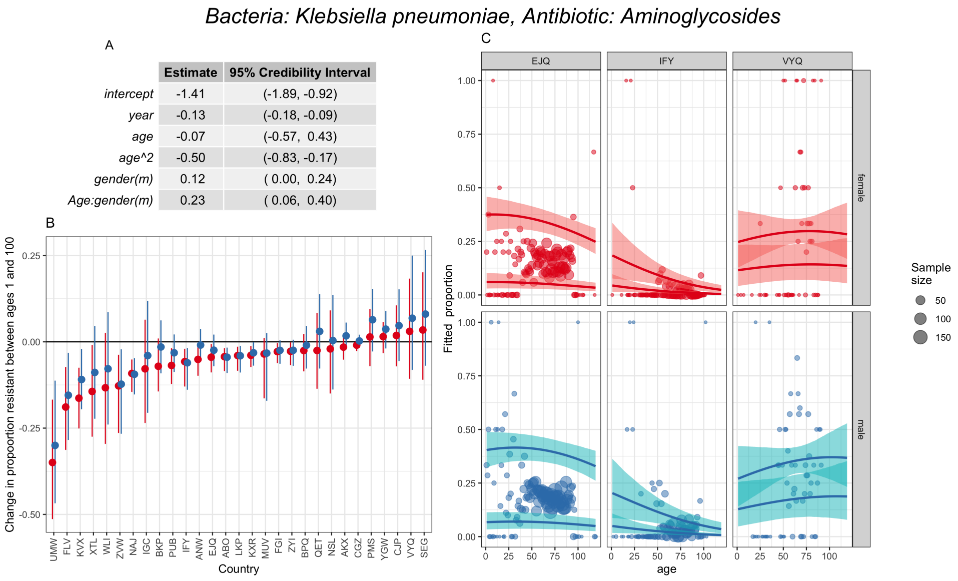

	Estimate	95% Credibility Interval
intercept	-1.41	(-1.89, -0.92)
year	-0.13	(-0.18, -0.09)
age	-0.07	(-0.57, 0.43)
age^2	-0.50	(-0.83, -0.17)
gender(m)	0.12	(0.00, 0.24)
Age:gender(m)	0.23	(0.06, 0.40)

Figure 17: *Klebsiella pneumoniae*, Aminoglycosides. A) Model parameters. B) Change in proportion resistant between ages 1 and 100 for each country and sex. C) Data (points) and model predictions (lines) with 95% CIs (ribbons) for the most extreme and the middle county from B. Each country has two lines, depicting the predictions for the most extreme laboratories in the country. Data is grouped across years and laboratories.

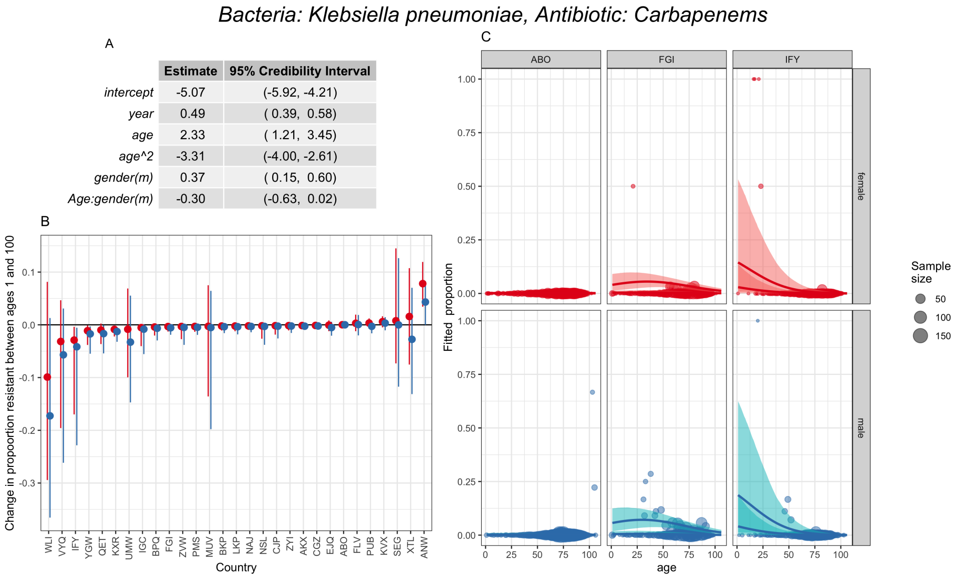

Figure 18: *Klebsiella pneumoniae*, Carbapenems A) Model parameters. B) Change in proportion resistant between ages 1 and 100 for each country and sex. C) Data (points) and model predictions (lines) with 95% CIs (ribbons) for the most extreme and the middle county from B. Each country has two lines, depicting the predictions for the most extreme laboratories in the country. Data is grouped across years and laboratories.

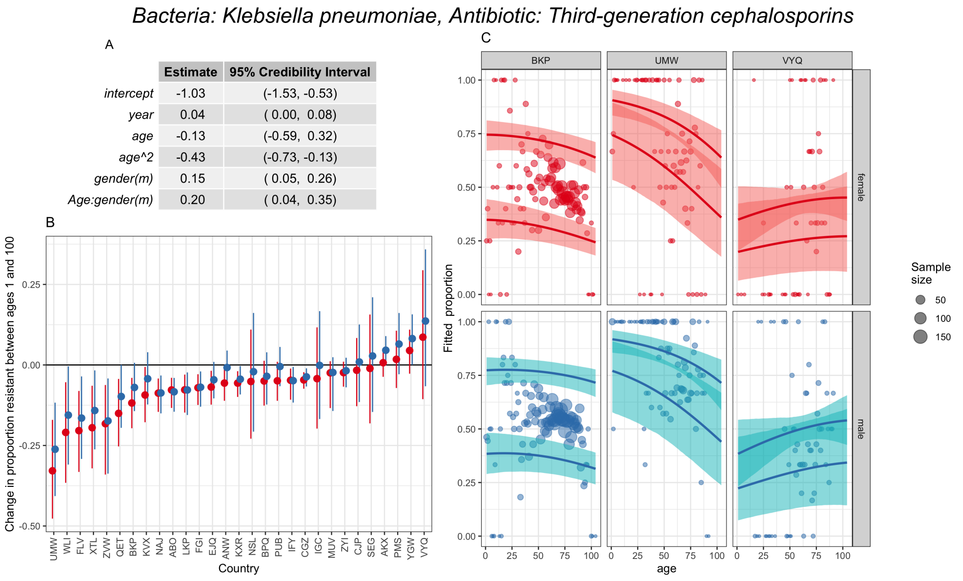

Figure 19: *Klebsiella pneumoniae*, Third-generation cephalosporins. A) Model parameters. B) Change in proportion resistant between ages 1 and 100 for each country and sex. C) Data (points) and model predictions (lines) with 95% CIs (ribbons) for the most extreme and the middle county from B. Each country has two lines, depicting the predictions for the most extreme laboratories in the country. Data is grouped across years and laboratories.

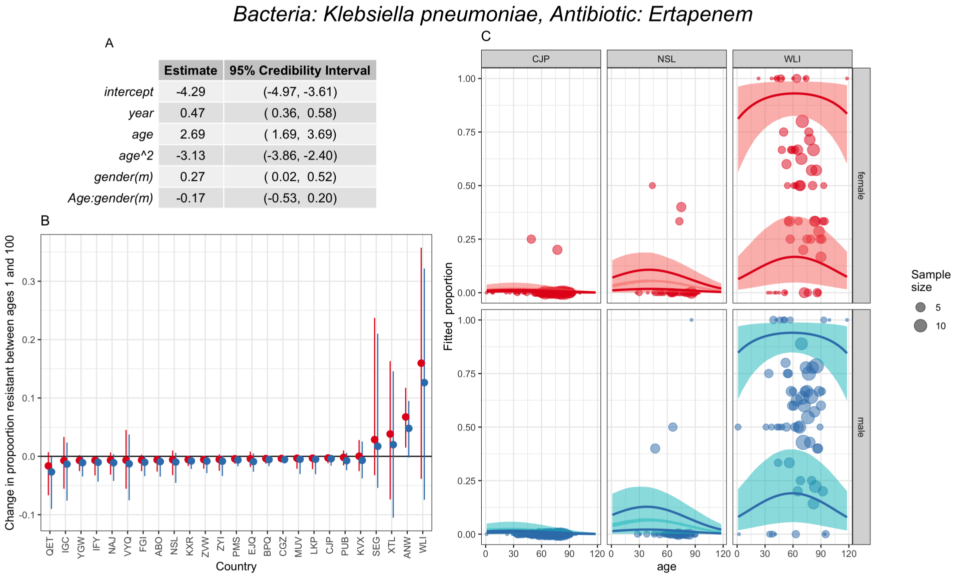

	Estimate	95% Credibility Interval
intercept	-4.29	(-4.97, -3.61)
year	0.47	(0.36, 0.58)
age	2.69	(1.69, 3.69)
age^2	-3.13	(-3.86, -2.40)
gender(m)	0.27	(0.02, 0.52)
Age:gender(m)	-0.17	(-0.53, 0.20)

Figure 20: *Klebsiella pneumoniae*, Ertapenem. A) Model parameters. B) Change in proportion resistant between ages 1 and 100 for each country and sex. C) Data (points) and model predictions (lines) with 95% CIs (ribbons) for the most extreme and the middle county from B. Each country has two lines, depicting the predictions for the most extreme laboratories in the country. Data is grouped across years and laboratories.

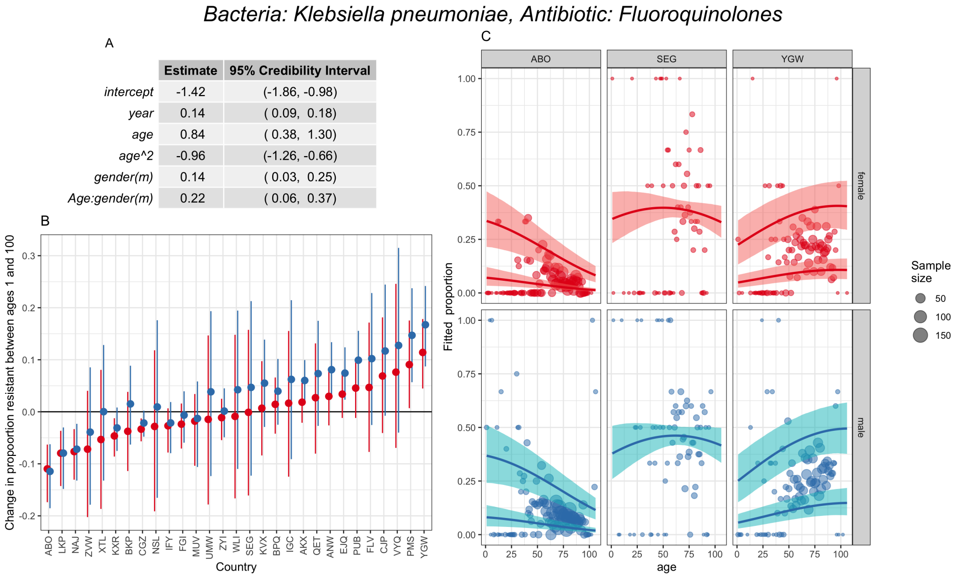

	Estimate	95% Credibility Interval
intercept	-1.42	(-1.86, -0.98)
year	0.14	(0.09, 0.18)
age	0.84	(0.38, 1.30)
age^2	-0.96	(-1.26, -0.66)
gender(m)	0.14	(0.03, 0.25)
Age:gender(m)	0.22	(0.06, 0.37)

Figure 21: *Klebsiella pneumoniae*, Fluoroquinolones A) Model parameters. B) Change in proportion resistant between ages 1 and 100 for each country and sex. C) Data (points) and model predictions (lines) with 95% CIs (ribbons) for the most extreme and the middle county from B. Each country has two lines, depicting the predictions for the most extreme laboratories in the country. Data is grouped across years and laboratories.

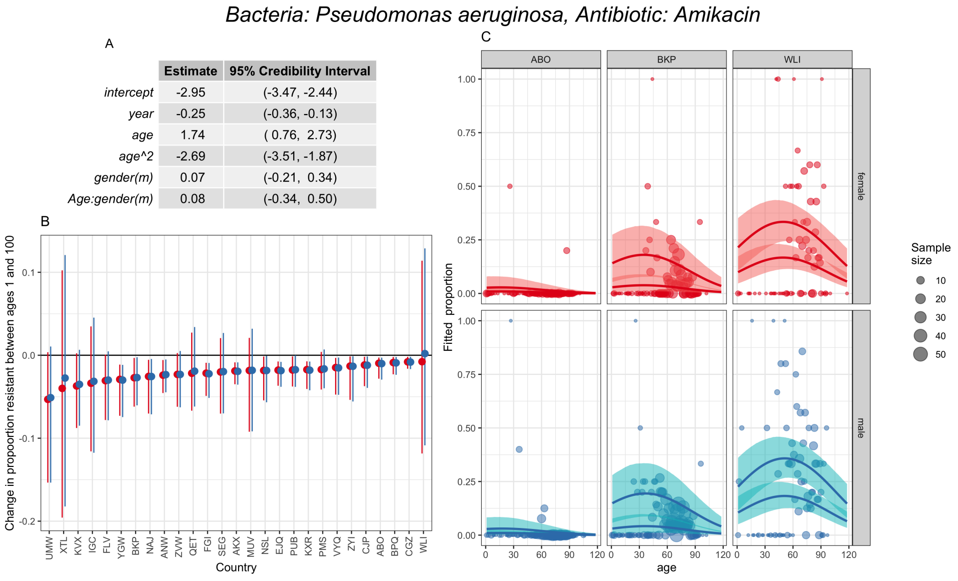

	Estimate	95% Credibility Interval
intercept	-2.95	(-3.47, -2.44)
year	-0.25	(-0.36, -0.13)
age	1.74	(0.76, 2.73)
age^2	-2.69	(-3.51, -1.87)
gender(m)	0.07	(-0.21, 0.34)
Age:gender(m)	0.08	(-0.34, 0.50)

Figure 22: *Pseudomonas aeruginosa*, Amikacin. A) Model parameters. B) Change in proportion resistant between ages 1 and 100 for each country and sex. C) Data (points) and model predictions (lines) with 95% CIs (ribbons) for the most extreme and the middle county from B. Each country has two lines, depicting the predictions for the most extreme laboratories in the country. Data is grouped across years and laboratories.

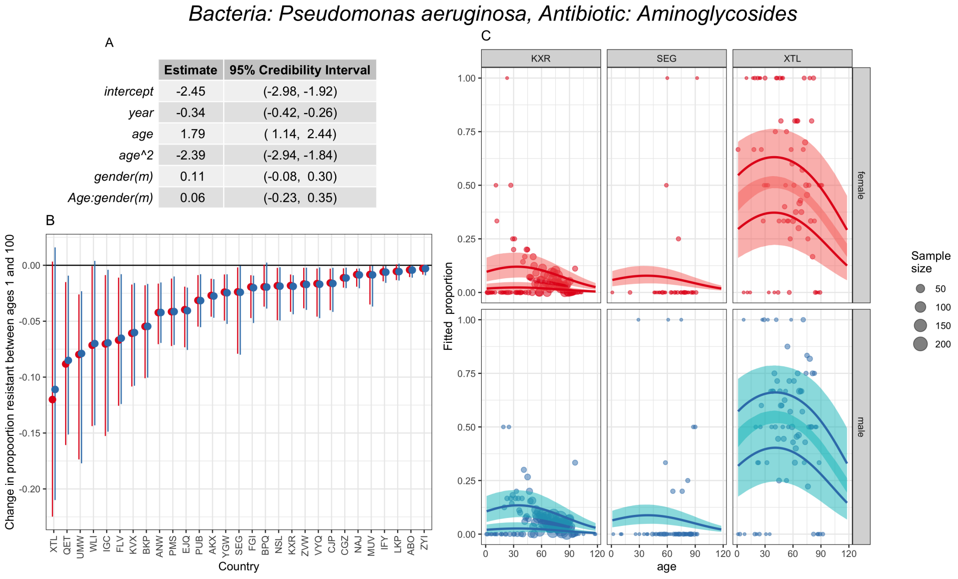

	Estimate	95% Credibility Interval
intercept	-2.45	(-2.98, -1.92)
year	-0.34	(-0.42, -0.26)
age	1.79	(1.14, 2.44)
age^2	-2.39	(-2.94, -1.84)
gender(m)	0.11	(-0.08, 0.30)
Age:gender(m)	0.06	(-0.23, 0.35)

Figure 23: *Pseudomonas aeruginosa*, Aminoglycosides A) Model parameters. B) Change in proportion resistant between ages 1 and 100 for each country and sex. C) Data (points) and model predictions (lines) with 95% CIs (ribbons) for the most extreme and the middle county from B. Each country has two lines, depicting the predictions for the most extreme laboratories in the country. Data is grouped across years and laboratories.

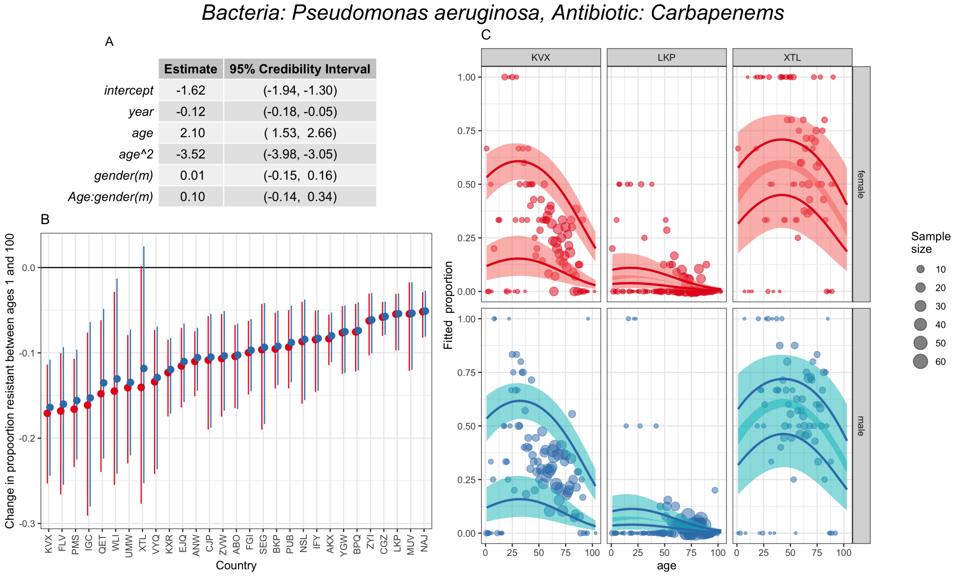

	Estimate	95% Credibility Interval
intercept	-1.62	(-1.94, -1.30)
year	-0.12	(-0.18, -0.05)
age	2.10	(1.53, 2.66)
age^2	-3.52	(-3.98, -3.05)
gender(m)	0.01	(-0.15, 0.16)
Age:gender(m)	0.10	(-0.14, 0.34)

Figure 24: *Pseudomonas aeruginosa*, Carbapenems. A) Model parameters. B) Change in proportion resistant between ages 1 and 100 for each country and sex. C) Data (points) and model predictions (lines) with 95% CIs (ribbons) for the most extreme and the middle county from B. Each country has two lines, depicting the predictions for the most extreme laboratories in the country. Data is grouped across years and laboratories.

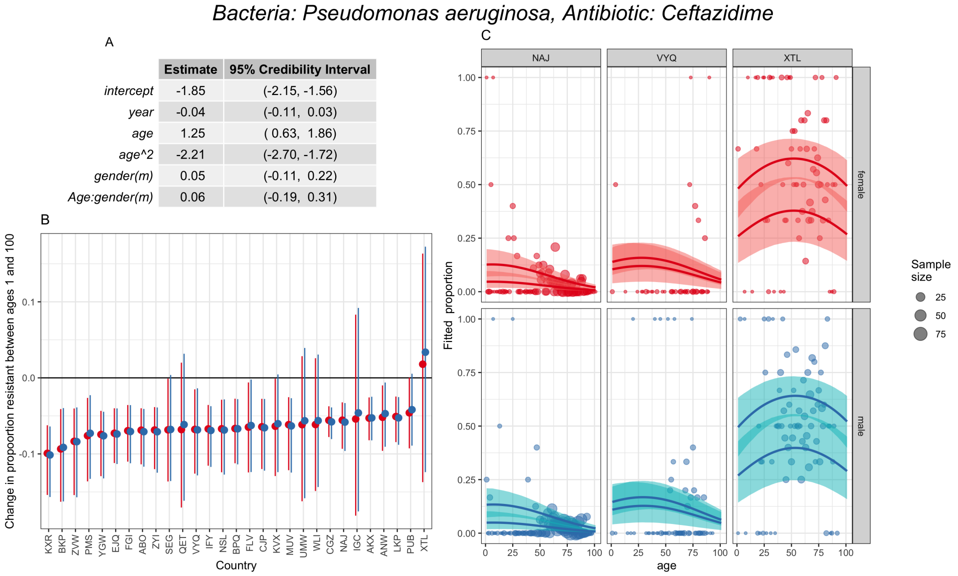

	Estimate	95% Credibility Interval
intercept	-1.85	(-2.15, -1.56)
year	-0.04	(-0.11, 0.03)
age	1.25	(0.63, 1.86)
age^2	-2.21	(-2.70, -1.72)
gender(m)	0.05	(-0.11, 0.22)
Age:gender(m)	0.06	(-0.19, 0.31)

Figure 25: *Pseudomonas aeruginosa*, Ceftazidime. A) Model parameters. B) Change in proportion resistant between ages 1 and 100 for each country and sex. C) Data (points) and model predictions (lines) with 95% CIs (ribbons) for the most extreme and the middle county from B. Each country has two lines, depicting the predictions for the most extreme laboratories in the country. Data is grouped across years and laboratories.

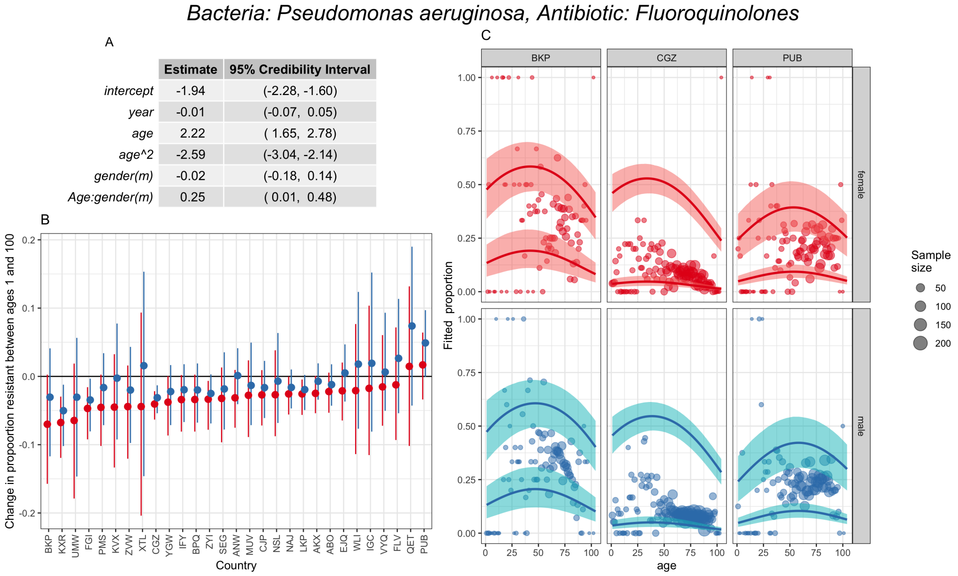

Figure 26: *Pseudomonas aeruginosa*, Fluoroquinolone. A) Model parameters. B) Change in proportion resistant between ages 1 and 100 for each country and sex. C) Data (points) and model predictions (lines) with 95% CIs (ribbons) for the most extreme and the middle county from B. Each country has two lines, depicting the predictions for the most extreme laboratories in the country. Data is grouped across years and laboratories.

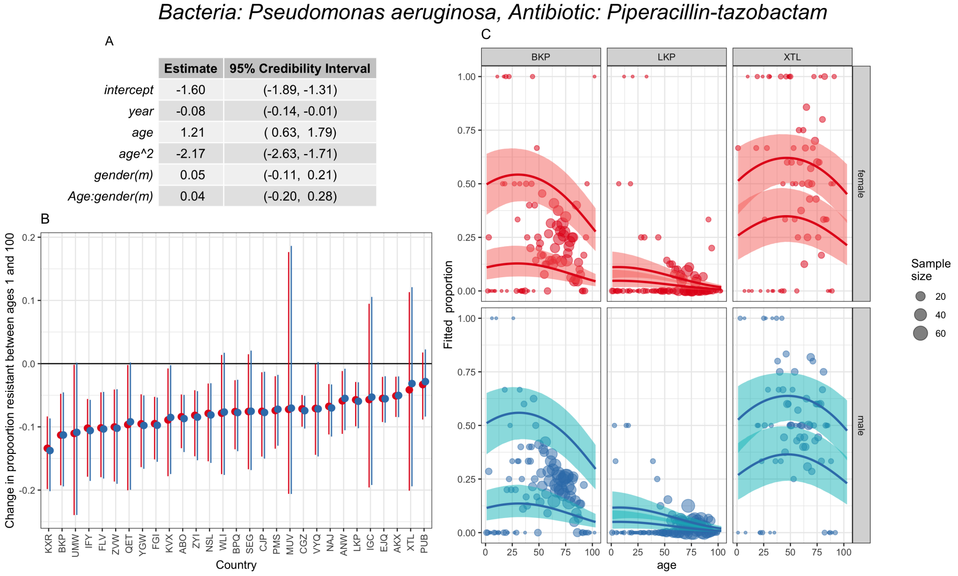

Figure 27: *Pseudomonas aeruginosa*, piperacillin-tazobactam. A) Model parameters. B) Change in proportion resistant between ages 1 and 100 for each country and sex. C) Data (points) and model predictions (lines) with 95% CIs (ribbons) for the most extreme and the middle county from B. Each country has two lines, depicting the predictions for the most extreme laboratories in the country. Data is grouped across years and laboratories.

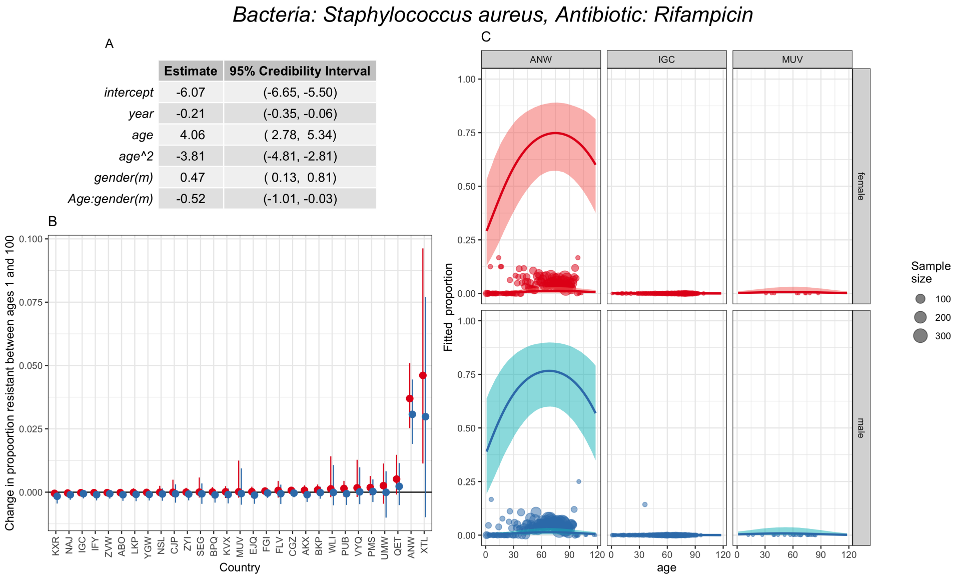

	Estimate	95% Credibility Interval
intercept	-6.07	(-6.65, -5.50)
year	-0.21	(-0.35, -0.06)
age	4.06	(2.78, 5.34)
age^2	-3.81	(-4.81, -2.81)
gender(m)	0.47	(0.13, 0.81)
Age:gender(m)	-0.52	(-1.01, -0.03)

Figure 28: *Staphylococcus aureus*, Rifampicin. A) Model parameters. B) Change in proportion resistant between ages 1 and 100 for each country and sex. C) Data (points) and model predictions (lines) with 95% CIs (ribbons) for the most extreme and the middle county from B. Each country has two lines, depicting the predictions for the most extreme laboratories in the country. Data is grouped across years and laboratories.

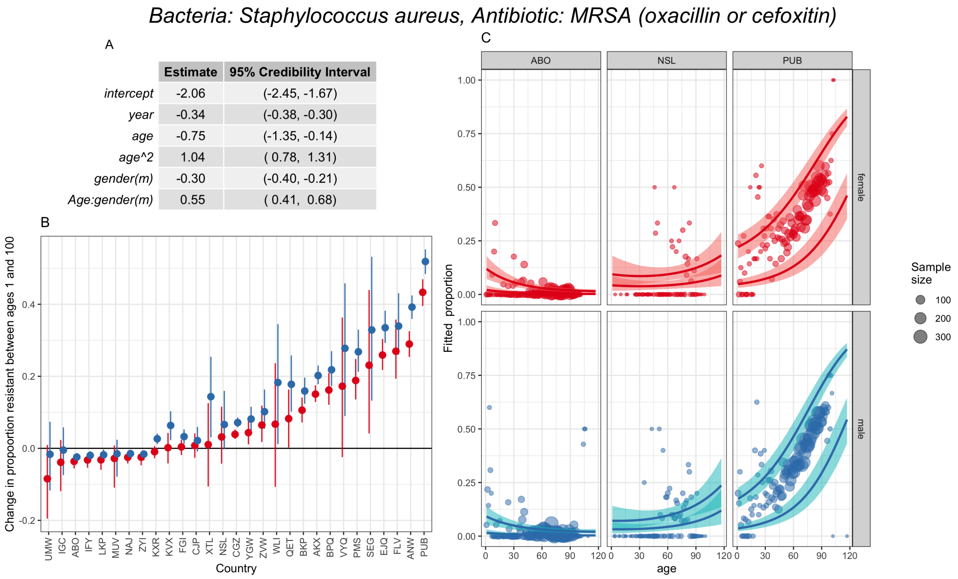

	Estimate	95% Credibility Interval
intercept	-2.06	(-2.45, -1.67)
year	-0.34	(-0.38, -0.30)
age	-0.75	(-1.35, -0.14)
age^2	1.04	(0.78, 1.31)
gender(m)	-0.30	(-0.40, -0.21)
Age:gender(m)	0.55	(0.41, 0.68)

Figure 29: *Staphylococcus aureus*, MRSA (oxacillin or cefoxitin). A) Model parameters. B) Change in proportion resistant between ages 1 and 100 for each country and sex. C) Data (points) and model predictions (lines) with 95% CIs (ribbons) for the most extreme and the middle county from B. Each country has two lines, depicting the predictions for the most extreme laboratories in the country. Data is grouped across years and laboratories.

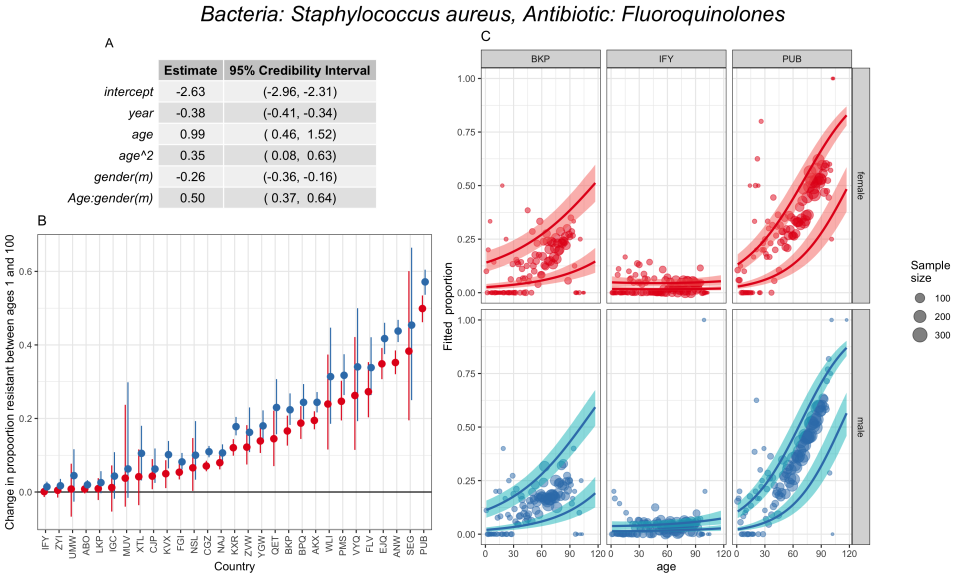

	Estimate	95% Credibility Interval
intercept	-2.63	(-2.96, -2.31)
year	-0.38	(-0.41, -0.34)
age	0.99	(0.46, 1.52)
age^2	0.35	(0.08, 0.63)
gender(m)	-0.26	(-0.36, -0.16)
Age:gender(m)	0.50	(0.37, 0.64)

Figure 30: *Staphylococcus aureus*, Fluoroquinolone. A) Model parameters. B) Change in proportion resistant between ages 1 and 100 for each country and sex. C) Data (points) and model predictions (lines) with 95% CIs (ribbons) for the most extreme and the middle county from B. Each country has two lines, depicting the predictions for the most extreme laboratories in the country. Data is grouped across years and laboratories.

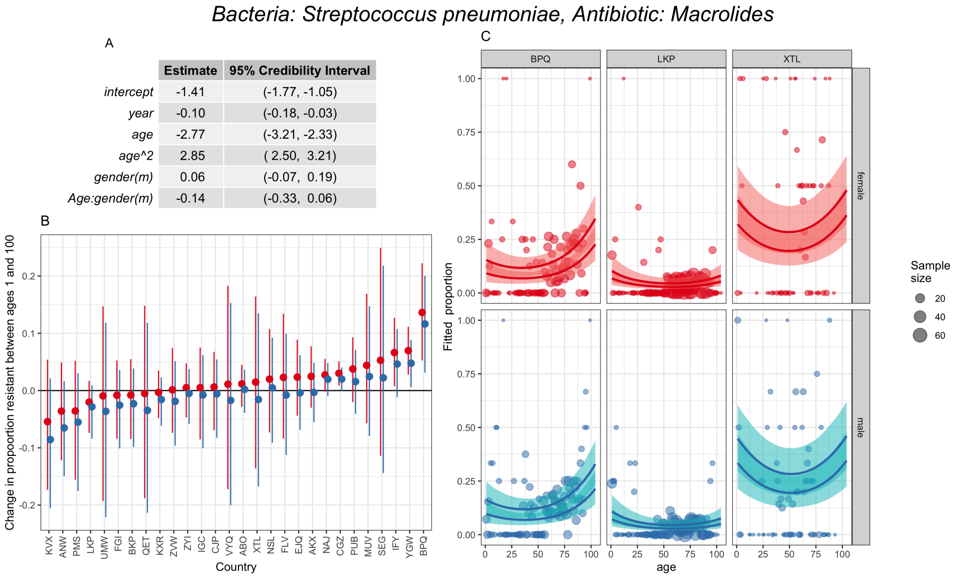

	Estimate	95% Credibility Interval
intercept	-1.41	(-1.77, -1.05)
year	-0.10	(-0.18, -0.03)
age	-2.77	(-3.21, -2.33)
age^2	2.85	(2.50, 3.21)
gender(m)	0.06	(-0.07, 0.19)
Age:gender(m)	-0.14	(-0.33, 0.06)

Figure 31: *Streptococcus pneumoniae*, Macrolide. A) Model parameters. B) Change in proportion resistant between ages 1 and 100 for each country and sex. C) Data (points) and model predictions (lines) with 95% CIs (ribbons) for the most extreme and the middle county from B. Each country has two lines, depicting the predictions for the most extreme laboratories in the country. Data is grouped across years and laboratories.

Bacteria: Streptococcus pneumoniae, Antibiotic: Penicillins

A

	Estimate	95% Credibility Interval
intercept	-1.49	(-1.80, -1.18)
year	0.10	(0.02, 0.18)
age	-3.34	(-3.82, -2.87)
age^2	2.93	(2.54, 3.31)
gender(m)	-0.04	(-0.18, 0.10)
Age:gender(m)	0.07	(-0.13, 0.28)

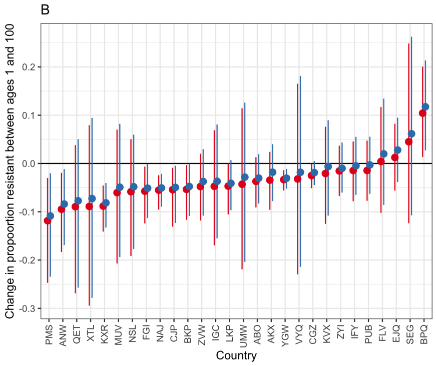

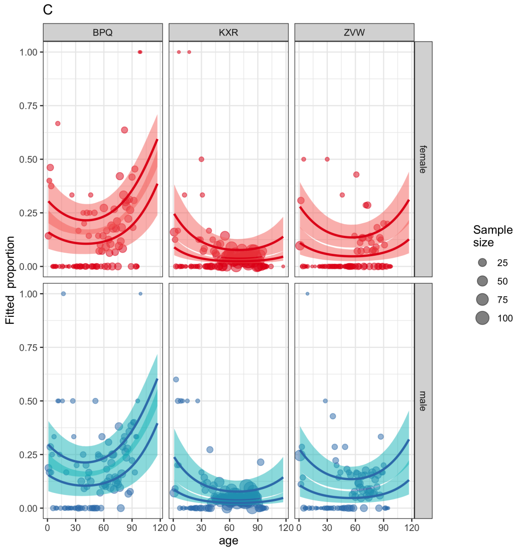

Figure 32: *Streptococcus pneumoniae*, Penicillins. A) Model parameters. B) Change in proportion resistant between ages 1 and 100 for each country and sex. C) Data (points) and model predictions (lines) with 95% CIs (ribbons) for the most extreme and the middle county from B. Each country has two lines, depicting the predictions for the most extreme laboratories in the country. Data is grouped across years and laboratories.

Bacteria: Streptococcus pneumoniae, Antibiotic: Fluoroquinolones

A

	Estimate	95% Credibility Interval
intercept	-7.24	(-8.14, -6.34)
year	2.20	(1.99, 2.41)
age	0.44	(-1.02, 1.89)
age^2	0.29	(-0.85, 1.44)
gender(m)	0.02	(-0.45, 0.49)
Age:gender(m)	0.15	(-0.53, 0.82)

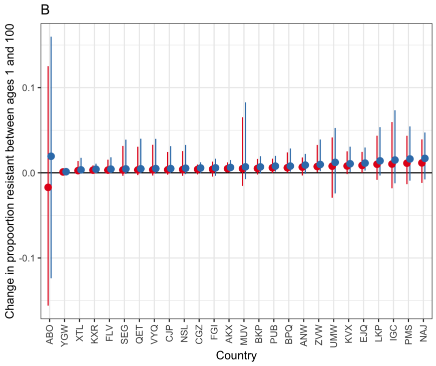

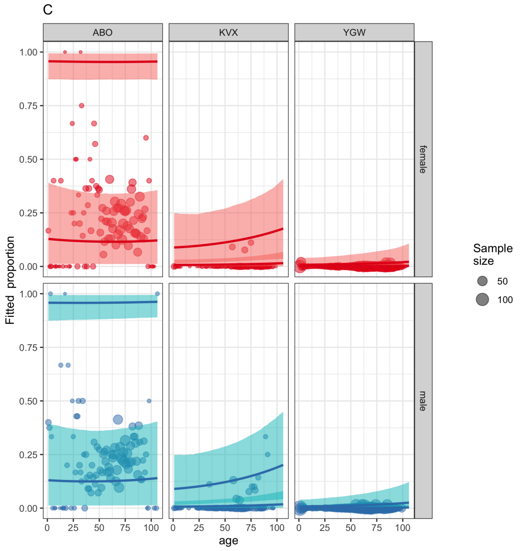

Figure 33: *Streptococcus pneumoniae*, Fluoroquinolones. A) Model parameters. B) Change in proportion resistant between ages 1 and 100 for each country and sex. C) Data (points) and model predictions (lines) with 95% CIs (ribbons) for the most extreme and the middle county from B. Each country has two lines, depicting the predictions for the most extreme laboratories in the country. Data is grouped across years and laboratories.
