# Supplementary material for: Antimicrobial resistance prevalence in bloodstream infection in 29 European countries by age and sex: An observational study
Source: PLoS Med. 2024 Mar 14;21(3):e1004301. doi: 10.1371/journal.pmed.1004301 (PMC10939247; doi:10.1371/journal.pmed.1004301)
Supplement: Attachment — Submitted filename: waterlow.pdf [file pmed.1004301.s004.pdf]

This paper describes the relationship between age and sex and antimicrobial resistance in Europe.

I was asked for a statistical report and I interpret that to include all aspects of the design and conduct of the study. The version I received did not have page numbers. My numbering has the abstract starting on page 2 although I have used the numbering provided within each Appendix.

Points of detail

Page 1 I might add 'in Europe' at the end of the title.

Page 5 I have not run the code (obviously I do not have the data) but it is extensively commented.

Page 5 Given the amount of missing data quoted in the Appendix S1 the decision not to use multiple imputation seems reasonable although it is a marginal decision.

Page 6 Have we already been told what BSI is? It is explained on page 15.

Page 7 The decision to estimate the difference between ages 1 and 100 strikes me as rather odd. Firstly it involves extrapolating beyond the range of data (since all 80+ are merged into one category). Secondly there are relatively few centenarians (although more than there used to be) so it is not a group of major public health interest. I could understand 1 to 80 better.

Page 7 I think there is a typo 'individual aged'. Should there be a zero here?

Page 8, Figure 1 This figure tries to convey a lot of information. Perhaps as a result it is in places quite hard to read. I am not an expert on microbiology but is it possible to split it into meaningful smaller figures? Even two might help. One thing which did not clearly stand out to me is which line is men and which women. I wondered whether using different line types might work better than colour but they might be hard to distinguish in the cloud of points unless the points can be made more transparent.

Page 10, Figure 2 It is hard to see the detail in Panel B as everything except *E coli* is squashed at the bottom. Would the authors consider plotting on a log scale? Less critical in panel A although some explanation of why this only covers up to age 50 might be helpful.

Page 11 I do not think the word effect is a good choice in the caption to Table 1. For me a positive effect on a harmful variable leads to it going down not up. There is also the issue that effect implies a causal relationship. Positive/negative coefficient might be better wording. Why not leave the current grey squares blank then the pattern of red and blue would be highlighted? That also avoids the issue of appearing to say there was evidence of no effect rather than no evidence of effect.

Page 15 I agree being able to use a finer classification of age is a positive advantage.

Page 17 I agree that using the same model throughout is a strength. Using some form of variable selection would make the models too data–dependent. I suspect that for the cases where the models failed to converge that simpler models would work but I think the principle of fixing on a model and standing by that decision is a good one.

Appendix S2, Page 2 This figure does not work I am afraid. The lines are almost invisible. If they are going to be provided then the points need to be made more transparent. The same applies to some extent to Figure 2 in this Appendix.

Appendix S2, Page 6 If you are going to say it is exponential growth then plotting on a log scale seems almost mandatory to me. This also applies to some of the later plots here.

Points of more substance

Country labels

Is the decision to obscure the identity of countries one imposed by the data providers? If not we need to know why it was thought necessary. Is the same code used for each country throughout? The captions to Figure 3 and Figure 4 do not clarify this. If it is necessary for whatever reason to de–identify the countries then why do various figures in Appendix S1 (eg F4 to F7) use the real names? Since we cannot cross–reference them to the other figures I am not sure what the point of those figures is.

Colour in graphs

I usually raise the issue of whether people with colour vision anomalies will have trouble with the graphs but in this case I think the avoidance of red–green as a distinguishing feature makes problem less likely. There is also

usually here a degree of redundancy which helps.

Summary

The degree of detail provided here and the clarity of description inspire confidence in what the authors present. My comments are mostly for clarification and suggestions for enhanced presentation.

Michael Dewey
