# Supplementary material for: Antimicrobial resistance prevalence in bloodstream infection in 29 European countries by age and sex: An observational study
Source: PLoS Med. 2024 Mar 14;21(3):e1004301. doi: 10.1371/journal.pmed.1004301 (PMC10939247; doi:10.1371/journal.pmed.1004301)
Supplement: Attachment — Submitted filename: 2312 Plos Med reviewers comments.docx [file pmed.1004301.s005.docx]

**Response to reviewers**

Thank you for the chance to edit and resubmit. In the below, the editor and reviewers comments are italicized.

We have added continuous line numbers and refer to those in the “Revised Article with Changes Highlighted” with track changes in “ALL MARKUP” mode (note there is a large jump in numbering at the end of the abstract).

Many thanks again,

Gwen Knight, on behalf of all co-authors

*COMMENTS FROM THE EDITORS*

*GENERAL*

*Please respond to all editor and reviewer comments detailed below in full. Please include line numbers starting at 1 on the title page and in continuous sequence throughout thereafter.*

*The editorial team agree that your study could be of significant value to the field. However, in its current form it does not flow well and is in places somewhat inaccessible to the reader which detracts from its impact. Please pay careful attention to the presentation of your manuscript, in particular the presentation of your methods and results when you revise. Specific comments are detailed below pertaining to each sub-section. We encourage you to review published articles on our website here* [*https://journals.plos.org/plosmedicine/*](https://protect-eu.mimecast.com/s/9e-UCA1pkcMrDjVt56ntq?domain=journals.plos.org) *and to refer to our guidelines on revising your manuscript which can be found here* [*https://journals.plos.org/plosmedicine/s/revising-your-manuscript*](https://protect-eu.mimecast.com/s/sO1CCB6q0U3PLvAi0CEyq?domain=journals.plos.org)

Many thanks for recognising the potential significance. We have worked through the manuscript aiming to improve the flow and addressed the reviewers specific comments. We hope the new version flows better. In particular we have focused on including more on the data that was analysed throughout.

*COMPETING INTERESTS*

*All authors must declare their relevant competing interests per the PLOS policy, which can be seen here:* [*https://journals.plos.org/plosmedicine/s/competing-interests*](https://protect-eu.mimecast.com/s/plDDCD8vnCQj8XMCpvA5C?domain=journals.plos.org)*. For authors with ties to industry, please indicate whether any of the interests has a financial stake in the results of the current study.*

We have no competing interests.

*TITLE: Please revise your title according to PLOS Medicine's style. Your title must be nondeclarative and not a question. It should begin with main concept if possible. "Effect of" should be used only if causality can be inferred, i.e., for an RCT. Please place the study design ("A randomized controlled trial," "A retrospective study," "A modelling study," etc.) in the subtitle (ie, after a colon).*

We have revised the title to: “Variation in antimicrobial resistance prevalence in bloodstream infection by age and sex: An analysis of European data”.

*ABSTRACT*

*Please structure your abstract using the PLOS Medicine headings (Background, Methods and Findings, Conclusions). Please combine the Methods and Findings sections into one section, “Methods and findings”.*

We have re-structured the abstract.

*Abstract Background: Please ensure that the background constitutes only one paragraph which includes context of the study importance. The final sentence should clearly state the study question.*

We have added a sentence on the aims of the study to the single background paragraph.

*Please remove details of the statistical approach/study design ad place in the methods and findings section instead.*

We have moved this to the methods & findings section.

*Abstract Methods and Findings:*

*Please clearly describe how you addressed your question/hypothesis. Please include details of the databases that you used to leverage data that you present/describe. Please include the study design, population(s) and setting(s), number of participants and/or samples included in your study, years during which the study took place, length of follow up, and main outcome measures.*

We have added information on the data used and number of samples / isolates used as well as the time period.

*Aggregate demographic details of the population studied would also be helpful (age range and mean and stratified according to sex) please include here instead of later in paragraph 2.*

We’re not sure where the information mentioned is in paragraph 2, but we have added age range and mean age stratified by sex into the abstract and now included all demographics in Table 1.

*Please ensure that you detail the full names of bacteria, Escherichia coli instead of E. Coli for example.*

We have edited this.

*Results paragraph 2: suggest instead, ‘aged between 1 and 100 years’ but as above, suggest including this information earlier along with the description of the study population. Please also see reviewer comments below regarding age cut-off.*

We have added a sentence about using this 1-100 comparison as a simplifying index as well as changing the wording. We have performed an additional robustness exploration for this index which shows that using different definitions of young vs older do not alter the main interpretations of these patterns (age and sex related trends).

*Results paragraph 2: ‘We explore our results in greater depths for two of the most clinically important bacteria–antibiotic combinations.’ would be better placed with the description of your methodological approach instead of the results section. Some additional clarification as to why these are ‘the most clinically important’ would be helpful to the non-expert reader.*

We have moved this sentence higher and include more in the main methods as to why we chose MRSA and *E. coli* (Line 593).

*Results paragraph 2: ‘At the country-level, the patterns are highly context specific with national and subnational differences accounting for a large amount of resistance variation…’ this is very vague – what, broadly, are the different contexts you refer to? Similarly, what are some examples of the differences you refer to? Please elaborate for clarity.*

We have removed the “highly context specific with national and” phrase as this really repeated the idea that there were national differences. The key piece of information is that there was a high level of subnational variation. We have also added “there are a range of functional forms for the associations between age and resistance prevalence” to clarify the differences we are referring to (line 57).

*The following sentence, ‘This diverges from the known, clear exponential increase in infection incidence rates by age…’ is this reference to data published elsewhere? If so, please remove. In the abstract, please refrain from including reference to data other than that generated by your study.*

­­ have edited this - to “For most bacterial species, there was a u-shaped pattern of infection incidence with age, which was higher in males. An important exception was *Escherichia coli,* for which there was an elevated incidence in females between the ages of 15 and 40. “ This is not a novel result but we wanted to include the analysis of incidence from this data to emphasise that the differences exist independently of this known increase in infections with age. We have also edited the text around Figure 2 to reflect this U-shape (line 668).

*Please ensure that all numbers presented in the abstract are present and identical to numbers presented in the main manuscript text.*

We have checked this.

*Please clearly define all statistical information for the reader. What does the numerical value, ‘…~0.46…’ represent? And, similarly what does the preceding symbol represent? Please clarify and define for the reader. Please define CI at first use for the reader.*

We have removed the “~” and have added in further information on the ranges: they are median with 95% quantiles so we have specified this.

*Please quantify the main results with 95% CIs and p values.*

As we aren’t testing a hypothesis, and as this is a Bayesian analysis we consider p values to be inappropriate in this case. Rather than confidence intervals we use credible intervals, appropriate for this analytical method. We have clarified what the “CIs” stand for (credible intervals) in the text.

*When reporting p values please report as p<0.001 and where higher as p=0.002, for example. If not reporting p values, to help facilitate transparent data reporting, please clearly state the reasons why not.*

We use a Bayesian approach and hence do not report *p* values.

*When reporting CIs please use commas as opposed to hyphens to separate upper and lower bounds as the latter can introduce confusion especially when reporting negative values.*

We have changed this throughout.

*Please include any important dependent variables that are adjusted for in the analyses. In the last sentence of the Abstract Methods and Findings section, please describe the main limitation(s) of the study's methodology.*

We have added in the levels for the multilevel regression model and added in the main limitations.

*Abstract Conclusions: As above, the conclusions could be slightly more nuanced regarding ‘context specific patterns’, please elaborate.*

We have removed this phrase.

*Please ensure that you address the study implications without overreaching what can be concluded from the data; the phrase "In this study, we observed ..." may be useful.*

We have included this phrase and linked directly to bloodstream infections in Europe.

*Please interpret the study based on the results presented in the abstract, emphasizing what is new without overstating your conclusions.Please avoid vague statements such as "these results have major implications for policy/clinical care". Mention only specific implications substantiated by the results.*

We have edited the conclusions to be more specific about interventions.

*Please avoid assertions of primacy ("We report for the first time....")*

We have removed “that have previously not been explored in detail” from the abstract. We have edited the novelty statement at the start of the Discussion (line 1287).

*AUTHOR SUMMARY*

*At this stage, we ask that you include a short, non-technical Author Summary of your research to make findings accessible to a wide audience that includes both scientists and non-scientists. The authors summary should consist of 2-3 succinct bullet points under each of the following headings:*

*• Why Was This Study Done? Authors should reflect on what was known about the topic before the research was published and why the research was needed.*

*• What Did the Researchers Do and Find? Authors should briefly describe the study design that was used and the study’s major findings. Do include the headline numbers from the study, such as the sample size and key findings.*

*• What Do These Findings Mean? Authors should reflect on the new knowledge generated by the research and the implications for practice, research, policy, or public health. Authors should also consider how the interpretation of the study’s findings may be affected by the study limitations. In the final bullet point of ‘What Do These Findings Mean?’, please describe the main limitations of the study in non-technical language.*

*Author Summary should immediately follow the Abstract in your revised manuscript. This text is subject to editorial change and should be distinct from the scientific abstract. Please see our author guidelines for more information:* [*https://journals.plos.org/plosmedicine/s/revising-your-manuscript#loc-author-summary*](https://protect-eu.mimecast.com/s/LCirCExwoHK1ZBnHRq-PH?domain=journals.plos.org)

We have now included a suggested author summary.

*INTRODUCTION: Please indicate whether your study is novel and how you determined that. If there has been a systematic review of the evidence related to your study (or you have conducted one), please refer to and reference that review and indicate whether it supports the need for your study.*

We did not do a systematic review of the evidence but instead would point to the many international agencies mentioned in paragraph 2 (line 318) that do not report AMR by age and sex for the novelty and lack of analysis in this area.

*Para 1: sentence beginning, ‘The higher burden…’ please provide a reference for this statement.*

We have included one (line 312).

*Para 2: ‘The recent WHO reports also have no presented analysis of how resistance prevalence varies by these demographic factors (8,8).’ Suggest ‘not’ instead of ‘no’. Please also check referencing/citations here, ‘…(8,8).’*

Thanks for highlighting this error - we have removed the double reference and changed to “not” (line 323).

*Penultimate paragraph: ‘…is key to understanding the complexity of AMR interventions.’ Not sure this accurately defines the point you are making. Perhaps instead, ‘…is key to understanding the nuanced interventions required to tackle AMR.’ or similar.*

We agree and have changed to the structure suggested (line 354).

*Final paragraph: opening sentence suggest, ‘Here, we use a large dataset of routinely collected information on bloodstream infections to explore trends in prevalence of antibiotic resistance and infection by age and sex across Europe.’*

We have changed to this sentence structure (line 363).

*Please remove the final sentences beginning with ‘It is vital…’. You could consider including these in the discussion section of your manuscript but, they are very subjective thus will require revision. We encourage you to include only objective statements substantiated by the data generated by your study.*

We have deleted these sentences and moved part of them to the final conclusion paragraph of the discussion.

*METHODS and RESULTS: A lot of nuanced information regarding what you did and why is missing from these sections. Please report the number of patients, samples, etc and dates of recruitment/inclusion, and account for all methods used in your study. Please revise in line with the specific comments detailed below.*

Thank you for highlighting this - we hope that we have revised the methods to include all needed details now in particular through the inclusion of Table 1.

*You state that ‘Countries were anonymized using a random 3-letter code, which is used throughout the paper’ why was this done? It seems unintuitive to present your results at country level and then to anonymize the countries. Please also see comments from the Academic Editor and the reviewer in relation to this point.*

In discussions with the curators of the EARS-NET data, the ECDC, we were told that we were unable to present the resistance prevalence in this level of detail by age and sex as it is not publicly available at the country level. In order to allow us to present it, and because our paper was about the overall trends in resistance, presenting and describing differences for the first time, rather than a detailed investigation as to the country-level reasons, we felt that presenting results by anonymised countries was still useful.

We have now included the following sentence as a justification in the methods section): “Countries were anonymised using a random 3-letter code, which is used throughout the paper as data at this level of detail by age and sex is not publicly available and we did not have permission to report it (8).“ (line 398).

*Methods opening paragraph: this would be better placed as the opening paragraph to your results section.*

We have moved this to the beginning of the results section.

*Sentence beginning: ‘All code is available…’ and ‘Patient level data is available…’ please remove these statements from the main manuscript and include only in the manuscript submission form. In the event of publication this information will be compiled as metadata.*

We have removed these sentences and will include them in the manuscript submission form.

*Methods paragraph 2: please delete. Please include only precise details of your methodological approach under appropriate sub-headings.*

We have deleted this paragraph. As this included information on the justification for using MRSA and aminopenicillin resistance in *E. coli* as our two key examples we have now added justification for these two later.

*Data paragraph 1: please define ‘ECDC’ at first use for the reader.*

We have included a definition in this paragraph now, as well as to ECDC in the Introduction and WHO earlier.

*In the opening paragraph please clearly state the years which data were extracted for analysis i.e. from when to when?*

We extracted data from 2015 to 2019. This is now included in line 393.

*In the appendix you present a vast amount of information regarding final participant inclusion as well as in respect to the isolates and stratified by country (albeit anonymized). But, I couldn’t see any detailed information either in the main manuscript or in the appendix regarding the population that the isolates of interest came from. The reader has to do a lot of jumping about to find nuanced information which should be readily available and accessible. Please revise accordingly.*

We apologise that in the main manuscript we did not focus on nor present the underlying data. We have now included a new Table 1 that has a summary of the distribution of susceptibility results. We hope this provides all the nuanced information that the reviewer requires in the main manuscript.

*Results opening paragraph: please include a paragraph which summarizes your study population including the total number, basic aggregate demographic details as well as a table of baseline characteristics of the total population and stratified by country in the main manuscript. You could also include relevant information pertaining to the isolates of the final population included.*

We have included a new table 1 which we hope includes all relevant information and commented on this in results (“Data” section).

*Resistance prevalence: please remove, ‘…supported clear benefits of including age in predictions of resistance in infection…’ which would be better place in the discussion as it is interpretive.*

Thank you for highlighting this - we have removed this and replaced it with “had” (line 784).

*‘Model analysis: examples’ and ‘Model analysis: general results’ – these are rather vague and uninformative. Please revise. What do you mean by ‘examples’ and ‘general results’? ‘Model analysis’ is poorly representative of what you present. What are you showing the reader? Please revise to include more informative sub-headings that clearly inform the reader of the content that follows.*

We thank the reviewer for highlighting this ambiguity and have changed the titles to “National and subnational variation in resistance prevalence” and “Variations in resistance prevalence with age by country, antibiotic and bacteria”. We hope that these better reflect the analysis shown.

*Sensitivity analyses: ‘Analysis…revealed large differences between countries across Europe’ please include the full data without anonymizing the countries. Please ensure that you provide detailed discussion of the potential reasons for the differences in observed outcomes in the discussion section.*

Unfortunately we cannot present the country level data in this manuscript as we do not have permission to reproduce this level of age / sex detail from the ECDC and the individual countries involved, hence the anonymisation. We have included a justification of this in the methods and more about the potential reasons in the discussion (lines 398, 1457).

*When reporting outcomes please quantify results with 95% CIs and p values. When reporting p values please report as p<0.001 and where higher, the exact p value as p=0.002, for example. If not reporting p values to help facilitate transparent data reporting please clearly state the reasons why not.*

We have used a Bayesian analysis here and so are not reporting *p* values. We have clarified that the CIs are credible intervals throughout.

FIGURES *Throughout, please consider avoiding the use of green and/or red to make your figures more accessible to those with colour blindness.*

We thank the reviewer for highlighting this - we have modified this to remove green from the figures (notably Figure 1 and Figure 5).

*Throughout you use the abbreviation ‘CI’ to refer to both confidence intervals and credible intervals. Please justify. Please ensure that CI is defined in all captions/footnotes for the reader.*

Thank you for highlighting this ambiguity we have adjusted this throughout and use “credible intervals” throughout.

*Throughout please indicate whether analyses are adjusted and if so, which factors are adjusted for. Where adjusted analyses are presented please also present unadjusted analyses for comparison.*

We perform no analyses using adjusted factors - we fit our multilevel model and present the results for this in all model output.

*Figure 1 – It isn’t at all obvious that the size of the points on the plots differ, largely due to the size of the individual graphs and, in addition no dots are black which makes the legend somewhat redundant and rather confusing please revise.*

We agree and have edited the colour of the dots to be more in line with what is shown on the graphs.

*The legend to depict male and female is also very confusing. The colours are obscured by the colours of the plots. Suggest using different lines to represent male and female and removing the colours as it is impossible to differentiate between the 2 groups in some instances.*

We thank the reviewer for this suggestion and have changed male/female to be differentiated by the linetype.

*When looking at the ‘columns of graphs’ what do the double headers refer to? Presumably the top graph is antibiotic class and the bottom the specific antibiotic example? Please clarify. And if, indeed if I am correct suggest either labelling the graphs separately or clearly defining this for the reader in the caption.*

Yes that was what the double headers referred to. However, we think this is a repeat of what is in the colour scheme and so have removed it in order to try and streamline this complex figure.

*The use of ‘rows’ and ‘columns’ to describe different graphs is somewhat unconventional, please revise.*

We have removed column and stated “panel” instead. We have kept “row” as the data for each bacteria is within a row but have added sub-figure legends to hopefully further distinguish the panels.

*Figure 2 – as above suggest using different black solid/dashed lines here also to represent male and female populations.*

We thanks the reviewer for this suggestion and have added a linetype difference here for the male-female distinction, as well as changing to a log scale.

*It is difficult to appreciate where upper and lower bounds of CIs end when using shading. Is there a better way to present these data? Please revise.*

The ranges presented here are the 95% confidence intervals from a LOESS fit (we have expanded the abbreviation in the legend now). These are not CIs from our model fit but a graphical smoothing function. We do not think that the upper and lower bounds of the CIs are useful to clearly distinguish - instead this is to show broad trends. Alternatives would be to show ranges on individual points (perhaps binomial confidence intervals) but we wanted to show lines with the confidence on the overall trend so have not edited this figure substantially.

*For ‘Pseudomonas aeruginosa’ the colour shading is too pale to be clearly visible, suggest revising your choice of colour palate.*

We agree and have edited this to make *P. aeruginosa* clearer.

*Figure 3 and 4 – please define ABO, NSL, PUB, KVX, BPQ in the footnote (if you type ‘country ABO’ into the Google search engine the headline is blood type by country…). In the footnote of figure 4 you state that these letters ‘are random and anonymized’ – why? This is not clear in your methods section. Please clarify and revise.*

We have had to anonymise the countries for data privacy issues and apologise that we did not state this in the methods section in the original version. We have now included a statement in our methods section (line 398).

*What does ‘CI’ refer to in the footnote? Please define for the reader. In part A, ‘upper 95%’ an ‘lower 95%’ what? Please define.*

We have expanded this now to emphasise that it is credible intervals.

*You could combine these into one column, separating with a comma, and completely define the data in the column header (confidence interval or credible interval) to improve accessibility.*

We agree and have simplified this table in the new Figure 3.

*Is the male and female legend required considering the labels on the graphs?*

We agree that it is not needed, and did not think that the presentation of both gender results was needed. We have removed the female panels in a simplified Figure 3.

*The grey shaded dots for sample size are misleading as there are no grey dots in these graphs. Please revise.*

We have kept the grey dots in as these refer to the sample size. We have included more information on these in the legend.

*Again, please refrain from referring to graphs as ‘columns’ you are not describing a table.*

We have edited this to be “panel”.

*Figure 5 – please clearly define the meaning of the dots and lines for the reader in the footnote/caption.*

We have added in the definition, thank you for pointing out this omission. We have also slightly altered the y axis groupings, which just alters the colours but shows the same results.

*TABLES: Please provide a table showing the baseline characteristics of the study population.*

We apologise for this omission and have now included details in Table 1.

*DISCUSSION: Please include a wider and more nuanced discussion of the differences that you observe, as per the reviewer and academic editor comments.*

We hope that we have now included more nuanced discussion as required. There is much that is unclear and little evidence to link to any of the possible drivers, but we hope we have clarified this more now.

*ACKNOWLEDGEMENTS: Please move the declaration to the competing interest section of the manuscript submission form.*

We are required by the ECDC to include this in the acknowledgement section as a condition of acquiring the data - please advise us on where to include this.

*Please define ECDC. Is an author affiliated to the ECDC? If so, please clearly state this by detailing their initials.*

We have expanded ECDC and no, no author is affiliated to the ECDC.

*REFERENCES: Please ensure that referencing follows our guidance which can be found here* [*https://journals.plos.org/plosmedicine/s/submission-guidelines#loc-references*](https://protect-eu.mimecast.com/s/18IZCG80qCoWzwqS6yvNl?domain=journals.plos.org)

*Please ensure that all journal name abbreviations are those found in the National Center for Biotechnology Information (NCBI) databases. For in-text reference callouts please place citations in square brackets and preceding punctuation as follows, ‘[1,3,].’ Please ensure all web references include an ‘Accessed [date]’ as opposed to ‘Cited [date]’.*

We have made these changes.

*SUPPORTING INFORMATION: Please cite your Supporting Information as outlined here:* [*https://journals.plos.org/plosmedicine/s/supporting-information*](https://protect-eu.mimecast.com/s/G7KiCJQBwUPpjxyFAq76u?domain=journals.plos.org) *In the published article, supporting information files are accessed only through a hyperlink attached to the captions. For this reason, you must list captions at the end of your manuscript file. You may include a caption within the supporting information file itself, as long as that caption is also provided in the manuscript file. Do not submit a separate caption file. Please ensure that all abbreviations used in figures are clearly defined for the reader including those used to depict statistical information.*

*APPENDIX 1: Supplementary figure 5 – it is impossible to differentiate the data pertaining to individual bacteria when grouped in this way. The bars are far too small. Please revise.*

We have made this a much bigger figure and included dashed lines to indicate key values on the x axis.

*Supplementary figure 6 – as above the graphs are too small to be easily accessible to the reader. Please revise. As for the main manuscript please refrain from describing the graphs as columns and rows.*

As this is not our data and the countries are anonymised in the main manuscript, we have removed this over-complicating figure which simply showed that trends vary between countries and leave the reference to the original data.

*Please follow the referencing format as outlined above for the main manuscript.*

We have made this change.

*APPENDIX 2: Figures - The same problems identified above (appendix 1) and in the main manuscript also apply here. Please revise in line with the previous comments to improve clarity and reader accessibility.*

We have edited the subregion figures to be clearer (Figure 1&2). The incidence figures are now on a log scale to address another comment.

*Table 1 – as for the main manuscript, please separate upper and lower bounds of CIs with commas as opposed to hyphens which introduce confusion when reporting negative values.*

We have changed to commas, thank you for this suggestion.

*COMMENTS FROM THE ACADEMIC EDITOR: This has potential to be a strong manuscript, and of high public health and clinical importance. However, I agree with you and the reviewers that major revisions are required. I would be happy to see a revised version resubmitted.*

*Specific comments that the authors should address:*

*1) Greater reflections and discussion on the public health importance of variation in incidence and prevalence by age, sex, and country. What do these findings imply needs to be done to reduce morbidity, mortality, and improve health? Right now, discussion of these issues is somewhat superficial, focusing on "context specific interventions" without mentioning what these could be, and what their likely impact would be. What do the authors recommend policymakers do with these results?*

We have removed any reference to context specific interventions (e.g. from abstract) and given clearer examples now on the age- and sex- targeting interventions that could be put in place. The aim of our analysis was to report patterns rather than determination of drivers for these patterns we are not convinced these findings can directly lead to recommendations on what policymakers should do, except that consideration of these results enable improved targeting for interventions; more analysis to uncover the drivers of these patterns would lead to further recommendations for intervention design and implementation. We have emphasised this further in the discussion (line 1363, “Only this level of data would enable determination of the local sources of AMR and hence optimal targeting of interventions.”).

*2) Needs a strong justification for why countries are anonymised. Right now, there is little value in the country-stratified estimates, as readers will not be able to interrogate the possible reasons for differences in trends between countries. So, recommend presenting country names, or if not possible (and I am not clear why it would not be possible), remove these analyses.*

As we’ve now outlined in the methods (line 398), we have to anonymise the countries as this level of data has not been published and we do not have permission from ECDC to publish it down to this level due to privacy concerns from the countries from which the data came. We have mentioned this in our limitations (line 1457) and the level of detail that is currently publicly available.

As the aim of this analysis was to provide a quantification of the potential associations between resistance prevalence, age and sex we believe it is important to show the national level variation even if we cannot link this directly to the country. We have added a sentence to the discussion now (line 1460): “However, reporting only the European level association would mask many patterns and hence we chose to show the results by anonymised country-level to emphasise the importance of continuing this analysis with country specific data in the future.”

*3) I don't understand why the analysis comparing people aged 0 and 100 years is included, or deemed to be interesting, particularly given the U-shaped distribution found for many antibiotics, and the very small number of 100 year olds. Recommend either providing a clear justification for why this is felt to be important (and is robust to extrapolation beyond the available data), or rework/remove this analysis.*

Given the inherent challenge in summarizing the variation in trends seen across all bacteria, antibiotics, countries, ages and the sexes, we explored use of different indices. We found that by using this extreme comparison of the difference between the model-predicted resistance prevalence in those 1 and 100 we could show clearly two aspects of the differences across countries: the difference in outputs by sex and the differences in age trends. In particular for our two example bacteria-antibiotic combinations we could extract nice examples of trends with heterogeneity.

The index uses model output and hence is not dependent on the number of people at each age, though to note we had similar numbers of sensitivity results for 1 and 100yrs as can be seen in the sample size circles in Figures 3&4 in the data.

We agree that this index does not capture the variation given U-shaped distributions. We explored developing multiple indices (such as age at peak resistance) but with countries being anonymous, we found that for this analysis they showed similar things: that there is substantial variation but that there are some broad sex / age trends (shown now in Figure 4).

We thank the reviewer for suggesting a robustness analysis: we found that changing from the definitions of younger / older from 100 or 1 actually made little difference to the ordering of the countries suggesting that we could use this to really say something about the order of the countries in terms of their differences in resistance prevalence in infections in the older vs younger populations.

To tackle this comment we have

- Included a new sentence in the methods (line 555): “This simplifying index was chosen to capture and illustrate one aspect of the differences seen (the change between young and old).”
- Separated out this analysis in a new arrangement of Figure 3 and Figure 4 to enable better comparisons to show the utility
- Performed an additional exploration of the robustness of this index by changing the comparator ages i.e. we compared model predicted resistance prevalence at (a) ages 50-100 to age 1 and (b) ages 1-20 to age 100
  - This results in a new analysis in the S2 appendix, section 12
  - And a new sentence in the results (line 1008): “The trends in this simplifying index by country were robust to comparing resistance prevalence at ages 50 to 100 to age 1, and at ages 1 to 20 to that at age 100 (S2 Appendix, section 5). ”
- Included a paragraph in the discussion about this index and the difficulty of exploring trends across this heterogeneity (line 1321).

*4) I agree with several reviewers that figures are overly complex, and difficult to interpret due to data overload and design choices. Please simplify and reduce the number of figures to convert selected important findings.*

We thank the reviewers for these comments and have hopefully simplified and streamlined the figures. We have decided to keep the same number of figures in the main manuscript (removing some in supplementary material) but have removed some information from the graphs (notably the sex-variation in Figure 3) and reordered the information in Figures 3&4 to tell a clearer story and enable easier comparisons.

*5) Greater discussion of the possible underlying causes of age-sex-country trends in prevalence and incidence is required. What do the authors think are the underlying causes of variation here? What evidence supports this? What specific research is now needed to better understand the drivers of these patterns?*

We have now included a paragraph on the country-level variation and some of the data that is available (lines 1434). However, we have little evidence as yet as to what could cause this variation - that is what this work aims to stimulate, the future work to pool and analyse age-sex disaggregated data to determine the drivers of AMR.

*6) Thanks for providing code. The "base" model is described as having "flat" priors. Looking at the code in the GitHub repo, I don't think that is correct. Models specify that the default `brms` priors are used, which are not flat. Given the large amount of data available, I am not clear why "flat" priors would be a useful modelling choice here, and this obviates the decision to use a Bayesian approach to analysis. Recommend the authors carefully review a) prior selection and b) terminology.*

We thank the reviewer for this comment. We have clarified that we used the default *brms* priors (line 549).

We did explore using weakly informative regularising priors in the sensitivity analysis and found that it had no effect on the posterior estimates for the main example of MRSA (S2 Appendix section 10). We think this is due to the fact that we used a lot of data and so the choice of priors should have had less of an effect.

We did discuss and debate adding in more informative priors but really had very little information on what to include for the priors. There really is very little data out there on how AMR varies by age, and what is there is often conflicting. We also used a broad modelling framework and so did not want to specify priors separately for each antibiotic-bacteria combination with little data, hence the use of the default priors.

The one area where we thought we would have good data was on the intercept - effectively the prevalence of resistance in BSI. However, many estimates we found for this were based or included the very data we used for the modelling and we could not separate this out as “prior” information.

*Comments from the reviewers:*

*Reviewer #1: See attachment*

We have checked the attachment and responded to the points raised there at the end of this response.

*Reviewer #2: This manuscript contains an analysis of the AMR prevalence and incidence patterns in Europe between 2015 and 2020, stratified by sex and age. It is clearly written and the exposition is of a high calibre. The importance of the work is also clear, as sex and age have not been systematically considered as risk factors for AMR in previous studies, except possibly the one that the authors cited. There are certain methodological concerns that should be addressed before it can be accepted, that except for the first two are mostly minor.*

We thank the reviewer for this overview and hope that we have addressed the concerns in the below responses and manuscript edits.

*The main concern has to do with the multiple hypothesis testing issue. Although this issue is less severe in the case of a multilevel Bayesian analysis, as was used in this manuscript, there is a need to explain why that is the case. A good, albeit somewhat dated, reference might be "The Statistical Crisis in Science" by Gelman and Loken, which highlights both pitfalls and ways around them. In particular, a specific concern is the inclusion of age^2 as an explanatory variable - the rationale for this seems unclear unless the authors believed from the start that the age dependency follows a quadratic curve (but their discussion of prevalence of infection increasing with age suggests otherwise). At a minimum, an additional sensitivity analysis without this variable would be recommended.*

We thank the reviewer for highlighting this and have aimed to address by both adding greater description of the reasons for including this term and completing the additional sensitivity analysis.

We have added a description of why we chose to include the age^2 term in the methods (line 538) - this is linked to the key driver of AMR, antibiotic use, having a quadratic relationship with age.

We performed a sensitivity analysis on model structure including a Model with no age terms (Model A) and a model with additional age effects (Model B). Comparing these to the outputs from the final model we used (Model D) showed that (using leave-one-out cross validation) Model D was always the best model for predicting the resistance prevalence within individuals.

We have now included the additional sensitivity analysis with a Model E, which is Model D without the age^2 term. The information criterion (S2 appendix section 12), shows that Model D would still be the most appropriate model, and shows that adding the age^2 term helped the model fit (line 1054).

*Secondarily, I am not entirely convinced that the uninformative priors used here are appropriate; the alternative of using a N(0,1) prior on the fixed-effect parameters could be described as a weakly regularising prior, but perhaps a strongly regularising prior could be used instead, given that nearly 10M datapoints are included in the study. There is a helpful discussion of the tradeoffs of regularisation via different priors at this link among several other places:* [*betanalpha.github.io/assets/case_studies/bayes_sparse_regression.html*](http://protect-eu.mimecast.com/s/VA7bCKQDxU08RL9SYb6Ut?domain=betanalpha.github.io)

We performed a sensitivity analysis using the weakly regularising priors of *normal(0,1)* for the MRSA example and found that it had no impact on the posterior estimates (S2 Appendix section 10). As there has been so little analysis of age- and sex-related trends in resistance, on data other than this European bloodstream data, we have little to better inform priors and hence have not included any further analysis.

*Other minor comments follow below.*

*1) In the main equation, if i is indeed the grouping level, then (according to the previous paragraph) it already includes country and lab, so are the indices c and l superfluous? Perhaps they could be omitted in the top equation and replaced by c(i) and l(i) in the bottom one when specific aspects of the grouping need to be referred to? Alternatively, if i refers to something else, please clarify its exact meaning in the equation.*

We thank the reviewer for this suggestion and have streamlined these equations.

*2) The residual error epsilon should be indexed by i, not t (unless the error is only time-dependent)*

We have indexed it by i.

*3) Please clarify that the interaction variable age * sex uses a 0-1 coding for sex (otherwise the product is meaningless), and specify which one is 0*

Apologies for missing this clarification we have included this now - yes it is 0 for females and 1 for males (line 537).

*4) The male-female colour distinction is hardly visible in Figure 1; either remove it altogether or enhance the contrast by zooming into the relevant parts of the plot*

Thank you for pointing this out - we have changed the male-female distinction to be line type.

*5) The fq_pseudo_R should be reserved for P. aeruginosa (Table 1), unless the exact same definition of fluoroquinolones was used for other species, in which case please state this explicitly;*

In the raw data, “fq_pseudo_R” was only used as an indicator for *P. aeruginosa* and *Acinetobacter.* We have added this into the supplementary around the new Table 2.

*additionally, it may be worthwhile combining tables 1 and 2*

Thank you - we think you are referring to the supplementary data and agree. We have combined these tables into one.

*6) In the introduction, the same citation is used twice, appearing as (8,8)*

We apologise and have corrected this.

*7) samples from individuals aged -> samples from individuals aged 0*

We have added the zero in, thank you for spotting this.

*8) of those there are many samples -> of those many samples*

We have changed, thank you for spotting this.

*9) The minimum of 1.4% untested is inconsistent with the maximum of 98.7% tested (likely due to rounding issues) in Table 1*

We agree and have checked the rounding in the combined S1 Appendix table 1: the maximum is 98.6% for carbapenem resistance in *P. aeruginosa.*

*10) and are also likely to have a BSI -> isolates do not have a BSI, patients do (also spell out the BSI acronym the first time it is used if you have not already done so)*

We have edited this and expanded BSI (page 14, S1 Appendix).

*11) Southern and Eastern generally -> Southern and Eastern Europe generally*

We have added in “Europe” on page 2 of S2 Appendix.

*12) "The patterns are still more similar" -> the first half of this sentence contradicts the second half*

We apologies for the ambiguity and have altered this to: “The patterns are still similar within a bacteria-antibiotic: i.e. the same across subregions within a bacteria-antibiotic and then different between bacteria-antibiotic combinations.” (page 2, S2 Appendix)

*13) Philosophical sensitivity analysis -> this sounds slightly unusual, although the intention is clear; structural (or methodological?) sensitivity analysis would probably be a better name*

We agree and have changed to “structural” sensitivity analysis.

*Reviewer #3: This is an important analysis and provides useful information. The work has been robustly conducted and is reported clearly and concisely. If published in PLOSmedicine it will be highly cited. I have a series of minor suggestions for the authors to consider.*

Many thanks for these comments. We hope we have changed sufficiently in line with your suggestions.

*Title : Quantification of the patterns and impact of variation in prevalence of resistance by age and sex: I am not sure the paper really addresses impact. The authors may want to consider removing this from the title*

We agree and have edited this. The title is now “Variation in antimicrobial resistance prevalence in bloodstream infection by age and sex: An analysis of European data”

*Introduction*

*Line 5 "rarely quantified" - no ref*

We have removed this statement.

*Para 2 line 7 (8,8) needs correcting*

Thank you for pointing this out, we have corrected this.

*Para 4 "Despite awareness of the importance of sex for many risk factors for infectious diseases (e.g. HIV) and bacterial infections such as urinary tract infections,…" I think this sentence is too general (sex links more widely to poor outcome from infection - and almost every other acute illness but specifically to risk of specific infections such as UTI) to be helpful and HIV is such a different disease. Suggest something like Despite sex being a well-established risk factor for specific bacterial infections such as UTI …*

We thank the reviewer for this suggestion and have changed to the text provided (line 339).

*Para 4 line 4 "studies of single bacteria" feels odd. Bacteria aren't really single. Suggest " infections caused by specific bacterial pathogens"*

We thank the reviewer for this suggestion and have changed to the text provided (line 341).

*Para 5 "by "factors such as governance and deprivation level" do you mean linked to or associated with? What is meant by governance level, this isn't clear to me at least.*

This comes from the large regression studies by Collignon et al and others, where they looked at things like “quality of governance” as measured by the World Bank. However, we agree that this is unnecessary detail and have removed “governance and” and just left it as a reference to deprivation level (line 348).

*Methods*

*These rely on EARs-NET data which are I think opportunistic, by which I mean labs submit data based on the clinical samples they receive. Differences in sampling and processing will therefore have the potential to create apparent differences between countries obscuring presence or absence of real differences.*

We agree and did mention this in our limitations, but have further emphasised it here with an additional emphasis on the routine surveillance basis for these data and the impact it may have (using the sentence suggested by the reviewer) in lines 46, 1466 and 1475.

*Para 3. In our main analysis we exclude individuals aged 0, due to their stark difference in immune dynamics and contact patterns, but run a sensitivity analysis including them.*

*This feels rather stark. Why 1 not 2? Or 10? Is there a better rationale or precident you can reference?*

Maternal antibodies wane by 12 months (now cited) and those children with bloodstream infections are those most critically ill and usually with increased healthcare contact. This raises their likelihood of carrying a resistant bacteria and hence making complex non-linear dynamics driven by a subset of ill children. We have altered this sentence to “In our main analysis we exclude individuals aged 0, due to their stark difference in immune dynamics, linked in part to waning maternal antibodies (1,2), and healthcare contact patterns of this subset of critically ill children, but run a sensitivity analysis including them.” (line 409)

*Results: "At the European level, there were clear non-linear differences in the prevalence of resistance in*

*infection by age and sex for different bacteria-antibiotic combinations (Figure 1)."*

*Figure 1 conveys a lot of information and could be made clearer: V hard to distinguish sex with colour - suggest you make one line dotted and the other continuous.*

We agree and have removed the colour distinction and replaced with a line type difference.

*Took me ages to note that the rows are gram negative and positive species respectively. Suggest you use these as headings rather than on the right y Axis so with a third top band to each of the 8 rows*

We have now added this information in by labelling each group of plots and adding this information in the legend. We hope that this makes it clearer. We have also removed the upper layer of antibiotic family as this is a repeat of the information in the colours.

*Why have you separated out amikacin from (other presumably?) amino glycosides*

Our groupings reflect the data that was provided. For Acinetobacter, *E. coli, K. pneumoniae* and *P. aeruginosa* we had both resistance data for amikacin and aminoglycosides and so present this data here. We have added a note on this in the supplementary material around Table 2.

*Ansamycin isn't a widely used term I think - rifamycins would be better?*

We have changed this to be rifamycin (a subset of ansamycins).

*I am not sure I agree with some of the text description of the trends shown in figure 1. There are more messages to draw out I think…The age-associated patterns varied more within drug-families than within certain bacteria (patterns within each colour are more different than within each row of Figure 1)*

*What about the penicillin and macrolide data for strep pneumo - you don't have data for these deugs for other organisms and they are v like eachother but very different from the other Strep pneumo graphs. These are the two first line drugs for CAP in most systems probably*

We agree that there are many messages and patterns to draw out. Thank you for highlighting the patterns in *S. pneumoniae.* We have added a sentence to emphasise the other pattern (similarity in subsets of drugs): “For some bacterial species, such as *S. pneumoniae*, resistance trends were similar for subsets of antibiotics” (line 640).

Linking to the potential impact of CAP treatment, we have added a sentence in the discussion on using these results to understand selective pressures: “Comparing and contrasting antibiotic combinations used for important syndromes, such as community acquired pneumonia, with the resistance patterns seen in common causative organisms could point to specific age-related selective pressures and hence targets for antibiotic stewardship.” (line 1319).

*"Sex has little impact on many of the age-related trends except for Acinetobacter species at younger ages, and E. coli and Klebsiella at higher ages"*

*Resistance among Acinetobacter peaks in young adult males across all classes and similar peaks are seen for klebsiella but not E coli. Pseudomonas resistance peaks in young adults of both sexes. There are no apparent gender differences for the gram positive species you have looked at. These data make me wonder about differences in healthcare contact (amount and nature)*

*I suspect that in Men and women have different quantitative and qualitative hospital contact at different points in their lives. In many healthcare systems women will have healthcare contact in relation to pregnancies. Young men may have different forms of contact - trauma for example. Chronic disease patterns will vary too. Are there high-level data e.g. on overnight hospital stays by age and gender and icu overnight stays?*

We thank the reviewer for these points - we mention trauma and the related potential for influence in the discussion (line 1421) and the potential pregnancy related influence of healthcare contact at the end of the introduction (line 353). We also cite the differences in sex that are available from the types of hospital admissions in the UK (ref 66). We have added a sentence to emphasize the importance of such analysis in future work: “Fundamental differences in healthcare contact by sex related to pregnancy and chronic disease patterns also need to be explored in relation to driving both selection and transmission differences in resistance prevalence.” (line 1500).

*If you hypothesize that biological sex rather than sex-related exposure is important in determining the differences you see then would you expect to see changes around the times of menarche and menopause? This is clear for E coli related presumably to UTI but otherwise?*

*Incidence: Figure 2 E coli - this is where you clearly see the impact of biological sex - increasing above males at menarche and converging again post menopause*

We have grouped these two comments together as we have addressed them in the same way by linking in hormone changes between menarche and menopause in the discussion section around the BSI changes for *E. coli* (line 1378). We have also called for future work into these differences - we believe that there is really little evidence on the link between sex, infection and AMR. GK is currently working with social scientists on a piece around what patterns would we *expect* to see in resistance given what we know about the potential direct and indirect impacts on use and transmission linked to gender. We have added the following sentence: “Future work is needed to quantify the contribution of biological sex versus sex-related exposure and societal influences on both the direct and indirect risks of infection and hence AMR.” (line 1378).

*Resistance prevalence*

*Why do age and age2 flip from being positive to negative? Understanding this paragraph requires quite a high level of statistical understanding. It would be worth a few lines of additional text to "unpack" the implications of this analysis.*

We thank the reviewer for this suggestion and have now included a plot in Appendix 2 that shows the decomposition of the model predictions into the separate components (Figure 5). We added to the results: “The non-linear trends in resistance prevalence by age were approximated in the model by a combination of linear and quadratic age effects, with variable combinations of associations leading to varying negative and positive coefficients (Table 2). The decomposition of these effects can be seen in Figure S5 (Appendix S2, section 4)..”

*Table 1 - why does high-level aminoglycoside appear twice for E. faecalis?*

Thank you for spotting this and apologies we left out *E. faecium* from the left hand column. This has now been corrected (in what is now Table 2) and in the appendix.

*Model analysis*

*Particularly for aminopenicillin resistance in E coli the dramatic differences between countries begs some explanation which is difficult with complete anonymity. Are there other analyses you could undertake to explore hypotheses eg grouping by European region? Population level data on use of aminopenicllins per capita or national policy information.*

We agree that it would be extremely useful to not anonymise the countries, but could not present this data here due to privacy concerns. We did look at subregion analysis (see Appendix 2) but many of the patterns were robust though affected (lowered / raised) by the North-West to South-East resistance prevalence gradient. We are attempting to get antibiotic use by age and sex across Europe but we have been told by ECDC that there is not robust data. It is something we are exploring but believe that for now it is future work.

We have added future work sentences (line 1497): “Future work is also needed to explore the national and subnational variation using age- and sex-disaggregated antibiotic usage data which is not currently widely available, as well as other national policy differences in infection control, antibiotic stewardship and demographic trends.”

Discussion

*"We find no universal trends, with variation in age and sex patterns across specific bacteria-antibiotic combinations and across national and sub-national contexts, suggesting that cultural factors dominate biological ones.*

*The exception being E. coli BSI in women? Worth highlighting this?*

Thank you for highlighting this ambiguity - we have now included “in resistance prevalence” as this is the point we wish to highlight here (line 1289). We discuss the *E. coli* BSI differences in women later (line 1374).

*Subnational is variably hyphenated as sub-national.*

Thank you for noting this - we have changed to subnational throughout the manuscript.

*"Transmission of MRSA often occurs in healthcare settings (52) and increased contact with such settings with age could explain our observed often positive trend in resistance proportions by age. "*

*This is a significant generalization and could explain why a small number of countries don't show the increase in MRSA with age. Are these areas with different epidemiology, less nosocomial and more community MRSA. You could explore this by looking at MRSA resistance to e.g. quinolones?*

We thank the reviewer for this comment - we are planning to explore combinations of resistance in future work. We could not look at hospital- vs community-associated infections with this data. We have added a further sentence at the end of this paragraph to touch on this (line 1302): “Exploring the contribution of community- vs hospital-associated infections (which we could not do with this data), as well as combinations of resistance could test this hypothesis and explain country-level variation.”

*"This may be particularly important in older adults, that often experience more severe consequences of bacterial infection (56)."They are also likely at greatest risk from AMR and I would argue that it may be more important in younger adults who may be at little risk of harms from AMR but in whom we avoid drugs out of concerns about AMR that could be beneficial. My example would be the avoidance of quinolone antibiotics driven largey by concerns about C difficile infection which is a disease which only affects older adults. So we treat younger adults with UTI using with agents like betalactams that are probably inferior in other ways.*

Thank you for this interesting angle on empiric prescribing stratification. We have added something on the potential improved options for younger adults (line 1349): “There could also be potential for improved treatment in younger adults through recommendations for the use of antibiotic which are currently limited across adults due to concerns about serious risks from resistant infections (driven by higher risk profiles for older populations) or other age-related concerns such as quinolone use linked *C. difficile* infections.”

*" When considering empiric prescribing guidelines, it is vital to not just consider the impact of prescribing the most appropriate drug, but also the impact of delaying prescription until more information is available, which may have catastrophic consequences in severe infections (Girard and Ely 2007)."*

*This is true but it doesn't seem very relevant - and the reference isn't cited correctly for some reason. Delaying is just one strategy so this feels like a fragment of unnecessary detail. Suggest you just delete it.*

We agree and have deleted this sentence.

Any attachments provided with reviews can be seen via the following link:

<https://www.editorialmanager.com/pmedicine/l.asp?i=867661&l=1TI1F7XD>

Comments from pdf in above link:

REVIEWER 1: *This paper describes the relationship between age and sex and antimicrobial resistance in Europe. I was asked for a statistical report and I interpret that to include all aspects of the design and conduct of the study. The version I received did not have page numbers. My numbering has the abstract starting on page 2 although I have used the numbering provided within each Appendix.*

*Points of detail Page 1 I might add ‘in Europe’ at the end of the title.*

We agree and have added European in the new title.

*Page 5 I have not run the code (obviously I do not have the data) but it is extensively commented.*

Thank you - we hope that it could be used by someone again if they have data access.

*Page 5 Given the amount of missing data quoted in the Appendix S1 the decision not to use multiple imputation seems reasonable although it is a marginal decision.*

Thank you.

*Page 6 Have we already been told what BSI is? It is explained on page 15.*

We thank the reviewer for highlighting this - we have expanded on BSI in the manuscript and two appendices on its first use.

*Page 7 The decision to estimate the difference between ages 1 and 100 strikes me as rather odd. Firstly it involves extrapolating beyond the range of data (since all 80+ are merged into one category). Secondly there are relatively few centenarians (although more than there used to be) so it is not a group of major public health interest. I could understand 1 to 80 better.*

We thank the reviewer for highlighting this - we have now performed comprehensive robustness analysis of this index finding that varying the upper limit from 100 down to even ~70 makes little difference. As this is comparison of model output it is not dependent on the number of individuals. We have included more information on this index in the methods (line 556) and in the results talk to the robustness (line 1010) shown in S2 Appendix new section 5.

*Page 7 I think there is a typo ‘individual aged’. Should there be a zero here?*

Apologies yes we have added in the number which should be 1 (linked to the above discussed index).

*Page 8, Figure 1 This figure tries to convey a lot of information. Perhaps as a result it is in places quite hard to read. I am not an expert on microbiology but is it possible to split it into meaningful smaller figures? Even two might help. One thing which did not clearly stand out to me is which line is men and which women. I wondered whether using different line types might work better than colour but they might be hard to distinguish in the cloud of points unless the points can be made more transparent.*

Based on comments from other reviewers we have edited this figure now and hope that it is more readable - we have removed some complexity on the panel labels, and made the gender difference a linetype.

*Page 10, Figure 2 It is hard to see the detail in Panel B as everything except E coli is squashed at the bottom. Would the authors consider plotting on a log scale? Less critical in panel A although some explanation of why this only covers up to age 50 might be helpful.*

We have added to the legend why we chose to focus on the first 50yrs of life (in order to get at the differences at earlier stages of life that are not clear from the lifelong trends and plotted the y axis as a log scale.

*Page 11 I do not think the word effect is a good choice in the caption to Table 1. For me a positive effect on a harmful variable leads to it going down not up. There is also the issue that effect implies a causal relationship. Positive/negative coefficient might be better wording.*

Thank you - we agree and have changed to coefficient.

*Why not leave the current grey squares blank then the pattern of red and blue would be highlighted? That also avoids the issue of appearing to say there was evidence of no effect rather than no evidence of effect.*

We have changed the grey squares to white.

*Page 15 I agree being able to use a finer classification of age is a positive advantage.*

*Page 17 I agree that using the same model throughout is a strength. Using some form of variable selection would make the models too data– dependent. I suspect that for the cases where the models failed to converge that simpler models would work but I think the principle of fixing on a model and standing by that decision is a good one.*

Thank you for these comments.

*Appendix S2, Page 2 This figure does not work I am afraid. The lines are almost invisible. If they are going to be provided then the points need to be made more transparent. The same applies to some extent to Figure 2 in this Appendix.*

We have edited this figure to match that in the main manuscript and hope this helps understanding.

*Appendix S2, Page 6 If you are going to say it is exponential growth then plotting on a log scale seems almost mandatory to me. This also applies to some of the later plots here.*

We have changed the incidence plots to be on a log scale throughout including Figure 2 in the main manuscript.

*Points of more substance*

*Country labels Is the decision to obscure the identity of countries one imposed by the data providers? If not we need to know why it was thought necessary. Is the same code used for each country throughout? The captions to Figure 3 and Figure 4 do not clarify this.*

Apologies for not including the reasons for the anonymisation - we have added this into the methods (line 398) and discussed this as a limitation in the discussion (line 1457).

Thank you for raising the consistency though - we have edited this in the captions (“*used for this study only but consistent across all analyses”)*

*If it is necessary for whatever reason to de–identify the countries then why do various figures in Appendix S1 (eg F4 to F7) use the real names? Since we cannot cross–reference them to the other figures I am not sure what the point of those figures is.*

The data presented in appendix S1 is from the previously published study Cassini et al. If we made the countries in these plots anonymised, using our code, then it could be possible to break our anonymisation code. We believe they are useful to include as they show the variation in coverage and support the methods we used in our incidence calculations. However we have removed the images linked to the World Bank data as that can be found in the World Bank publications and was not used to make methodological decisions here.

*Colour in graphs I usually raise the issue of whether people with colour vision anomalies will have trouble with the graphs but in this case I think the avoidance of red– green as a distinguishing feature makes problem less likely. There is also a degree of redundancy which helps.*

Thanks for this comment - we have actually changed our palettes in line with another reviewer’s comment and hope that we have further removed any red-green issues.

*Summary The degree of detail provided here and the clarity of description inspire confidence in what the authors present. My comments are mostly for clarification and suggestions for enhanced presentation. Michael Dewey*
