# Supplementary material for: Antimicrobial resistance prevalence in bloodstream infection in 29 European countries by age and sex: An observational study
Source: PLoS Med. 2024 Mar 14;21(3):e1004301. doi: 10.1371/journal.pmed.1004301 (PMC10939247; doi:10.1371/journal.pmed.1004301)
Supplement: Attachment — Submitted filename: response_to_reviewers.docx [file pmed.1004301.s006.docx]

RESPONSE TO REVIEWERS

*Thank you for these comments which have improved the clarity of manuscript again. We have responded in italics below to all comments, made minor grammatical edits and uploaded new versions of Figure 1 and 5.*

COMMENTS FROM THE ACADEMIC EDITOR

I have read through the manuscript this morning, and it is much improved. Particularly the much more detailed focus on public health and future research implications of their findings.

My remaining comments are mostly minor and relate to the Figures, which I think could still be simplified and improved for clarity. In principle, I would be happy to progress to acceptance.

*Many thanks for your comments. We have modified the figures in line with your suggestions.*

1) Figure 1. There is really no need to show the points here. As there is so much data, they obscure the fitted curves and uncertainty intervals for sex-specific trends. I would either a) removal the points altogether, and just show the sex-specific curves and uncertainty intervals, or b) make the points black, increase the alpha substantially, and vary the shape by sex.

*We believe that we do need to show the data in this figure as this is a data figure - the smoothed lines are not from our model output but a graphical simplification of the data. To address this we have followed route (b) - the points are now grey, the alpha is increased and the shape varies by sex. We hope this addresses the comments.*

For black panels, need to add to the footnote to state that "no data available" (rather than the current ambiguous phrasing).

*We have included this in the legend (presuming that “blank” not “black” panels are referred to).*

Footnote still states "confidence interval" - is this correct? Should it be "credible interval"?

*Yes, this is still confidence interval - this is calculated as the confidence of the fitted line not as an output of our models. We have included more information there.*

2) Figure 5.

I struggled with interpreting this figure. E.g. in Panel A, why so some bacterial have only two estimates, and some have more (e.g. up to 6). i.e. the points and uncertainty intervals clear map to something, but not clear what it is. Should be distinguished by e.g. a different shape of the point.

*Thanks for raising this confusion. The original graph had different numbers of estimates per bacteria due to different number of antibiotic resistance results per bacteria (e.g. only resistance to 3 drugs were reported for S. aureus but 7 for E. coli).*

*We have modified the figure now so that there is less repetition and the points / colours represent the same thing across the figure. Instead of using figure 5a to show the gram stain differences and figure 5b to show drug differences, we have split the figure to be gram negative top and gram positive bottom. The drug differences are still shown with colours, but the colours (the antibiotic families) now apply across the figure.*

*We hope this is a clearer figure now that allows the reader to make their own conclusions about the effect of age within bacteria, within gram stain grouping and within drug families.  We have altered the text to support this.*

*In exploring ways to improve this figure we realized that we had left out the results for fluoroquinolones in Streptococcus pneumoniae from this figure and from previous results. This has been modified in this figure (Fig5), Table 2 and corresponding supplementary figures.*

Change in proportion label in X-axis: should be clearly stated whether this is relative change in proportion, or percentage point change.

*The x axis values is the change in proportion - we hope that by adding in “model-predicted” it is clearer.*

3) It is disappointing that the ECDC would not give permission for identification of countries, as this does weaken the insights that can be drawn from the results. I am sure readers will pick up on this too. I think as currently presented, this is fine, but from a journal perspective, I wonder if getting written evidence that ECDC have indeed prohibited release of these aggregate data might protect against future questions from readers?

*I have emailed them to ask but not had a response. We could link to the dashboard page to show that the open data is not at this level of disaggregation or try a joint email from the journal to ECDC which might carry more weight?*

COMMENTS FROM THE EDITORS

GENERAL

Thank you for your considerate and detailed responses to previous editor and reviewer comments. Please see below for further comments which we require you address prior to publication.

In respect of the Academic Editor’s 3rd comment above, please include relevant supporting information.

*Thank you for strengthening the manuscript. I have emailed the ECDC but have yet to have a reply.*

TITLE

Thank you for revising your title, we suggest the following, “Prevalence and characteristics of antimicrobial resistance in bloodstream infections in 29 European countries: An observational study”

*Many thanks for this suggestion. Would it be possible to keep age and sex in the title? These are the key indices that we believe should be considered going forward in antimicrobial resistance research and hence would like to make the title less general?*

*Could we use*

*“Antimicrobial resistance prevalence in bloodstream infection in 29 European countries by age and sex: an observational study” ?*

ABSTRACT

Please detail that your data is representative of 29 countries.

*We have added this to the abstract.*

Line 33 – ‘6,862,577 susceptibility results’ please clarify how many participants this pertains to.

*In EARSNET, the first isolate per patient, year, and bacterial species is collected. Each isolate may be tested for resistance to different antibiotics. We have added this information into the text and into Table 1.*

Line 35 – ‘with a similar age distribution in both sexes’ – I couldn’t see the age range detailed, please include.

*This was included in Table 1 and is now included in the text.*

Line 39 – please detail the bacteria-antibiotic combinations you refer to here.

*We have added this in.*

Line 41 – please remove the limitations to the end of the methods and findings section.

*We have moved it to the end.*

Line 45 – would an example of a bacterial species you refer to also be helpful (perhaps one that is targeted by fluoroquinolones?)

*We have included Pseudomonas aeruginosa.*

Lines 52-54 – please quantify the percentages (as above, it is not mentioned in the abstract how many countries your study investigates).

*We have added in the number of countries (n=xx).*

AUTHOR SUMMARY

Thank you for including an author summary.

Line 79 – suggest beginning, ‘We fitted…’

*We have changed to this suggestion.*

Lines 81-83 – suggest combining as follows, ‘Distinct patterns in resistance prevalence by age were observed across Europe for different bacteria.

*We have simplified as suggested.*

Line 84 suggest – ‘[Female] sex was most strongly associated with E. coli and K. pneumoniae resistance [in older age groups], and at younger ages for Acinetobacter species.’ Which sex most strongly associated with which resistant bacteria (male or female)? Please include details as indicated. Please also detail the full names of the bacteria. Is resistance of E coli and K pneumoniae noted mostly in older people? If so, please detail as suggested above.

*We have modified this to “Sex was often only weakly associated with resistance, except in Escherichia coli and Klebsiella pneumoniae where across ages it was higher in males, and at younger ages for Acinetobacter species where it was higher in males.”*

Lines 87 onwards are a little repetitive and somewhat vague. And, although earlier you refer to sex and age differences here you comment only on the implications of age and as such these remarks seem incomplete. Please revise to provide a more nuanced and balanced interpretation of your findings.

*We have revised these bullet points.*

In the final bullet point of ‘What Do These Findings Mean?’, please describe the main limitations of the study in non-technical language.

*We have added in a limitation bullet point.*

METHODS and RESULTS

As for the abstract please clarify and provide details of the number of participants the susceptibility results pertained to.

TABLES

Table 1 – PLOS Medicine requests that means and SDs are reported, please include. If there a specific reason that you choose to report the median then please give details.

*We have changed all values to the mean (age and number of results) in Table 1.*

As above, please clarify and provide details of the number of participants the susceptibility results pertained to.

*We have added a row in the table for total, females and males.*

FIGURES

We agree with the Academic Editor regarding the presentation of your figures which we think could be improved. Please revise.

*We have edited these figures in line with the comments - see response above.*

DISCUSSION

Please revise the opening paragraph of you discussion such that it begins with a short, clear summary of the article's findings; followed by what the study adds to existing research and where and why the results may differ from previous research; strengths and limitations of the study; implications and next steps for research, clinical practice, and/or public policy; one-paragraph conclusion.

*We have modified the beginning of the Discussion.*

Line 672 – please place a header which reads, ‘Disclaimer’.

*We have added this.*

SOCIAL MEDIA

To help us extend the reach of your research, please detail any X (formerly Twitter) handles you wish to be included when we tweet this paper (including your own, your coauthors’, your institution, funder, or lab) in the manuscript submission form when you re-submit the manuscript.

@N_R_Waterlow

@gmknght

@JVRobotham

@bugwonk

COMMENTS FROM THE REVIEWERS:

Reviewer #1: The authors have addressed all my points

Michael Dewey

Reviewer #3: Thank you for your thorough approach to the reviewers' comments. The manuscript is considerably strengthened. I have no further comments.

*Thanks to both reviewers for their help with the manuscript.*
